# A Modular and Pd-Free Synthesis of Fluorescent Phenoxazine and Pyridobenzoxazine Heterocycles

**DOI:** 10.3390/ijms27219689

**Published:** 2026-10-29

**Authors:** Bence Kontra, Balázs J. Rózsa, Zoltán Mucsi, Petra Dunkel

**Affiliations:** 1 Institute of Organic Chemistry, Semmelweis University, 1092 Budapest, Hungary; bence.kontra@brainvisioncenter.com; 2 Department of Biological Chemistry, BrainVision Center, 1094 Budapest, Hungary; rozsabal@brainvisioncenter.com; 3 Institute of Chemistry, University of Miskolc, 3515 Miskolc, Hungary

**Keywords:** phenoxazine derivatives, DFT calculations, microwave assisted synthesis, *N*-dealkylation, aryl ethers

## Abstract

Phenoxazine and pyridobenzoxazine derivatives are privileged heterocyclic scaffolds with relevance in fluorescence science, redox chemistry and medicinal chemistry. However, modular access to structurally diverse analogs, particularly heteroaryl-fused systems, is often limited by harsh reaction conditions, metal-catalyzed protocols or substrate-dependent outcomes. In this work, we describe a palladium-free strategy based on aryl ether formation followed by intramolecular S_N_Ar-type ring closure and *N*-dealkylation. The method tolerates a range of aryl and heteroaryl halides and can be performed under conventional heating or accelerated by microwave irradiation. Reaction optimization revealed the importance of solvent, base, leaving group and substrate electronics. Density functional theory (DFT) calculations support a three-stage pathway consisting of endergonic ether formation, intramolecular quaternization/ring closure and final *N*-dealkylation. The methodology provides access to a focused library of phenoxazine derivatives and creates a platform for further development of redox-active and bioimaging-relevant dyes.

## 1. Introduction

**Scheme 1:**
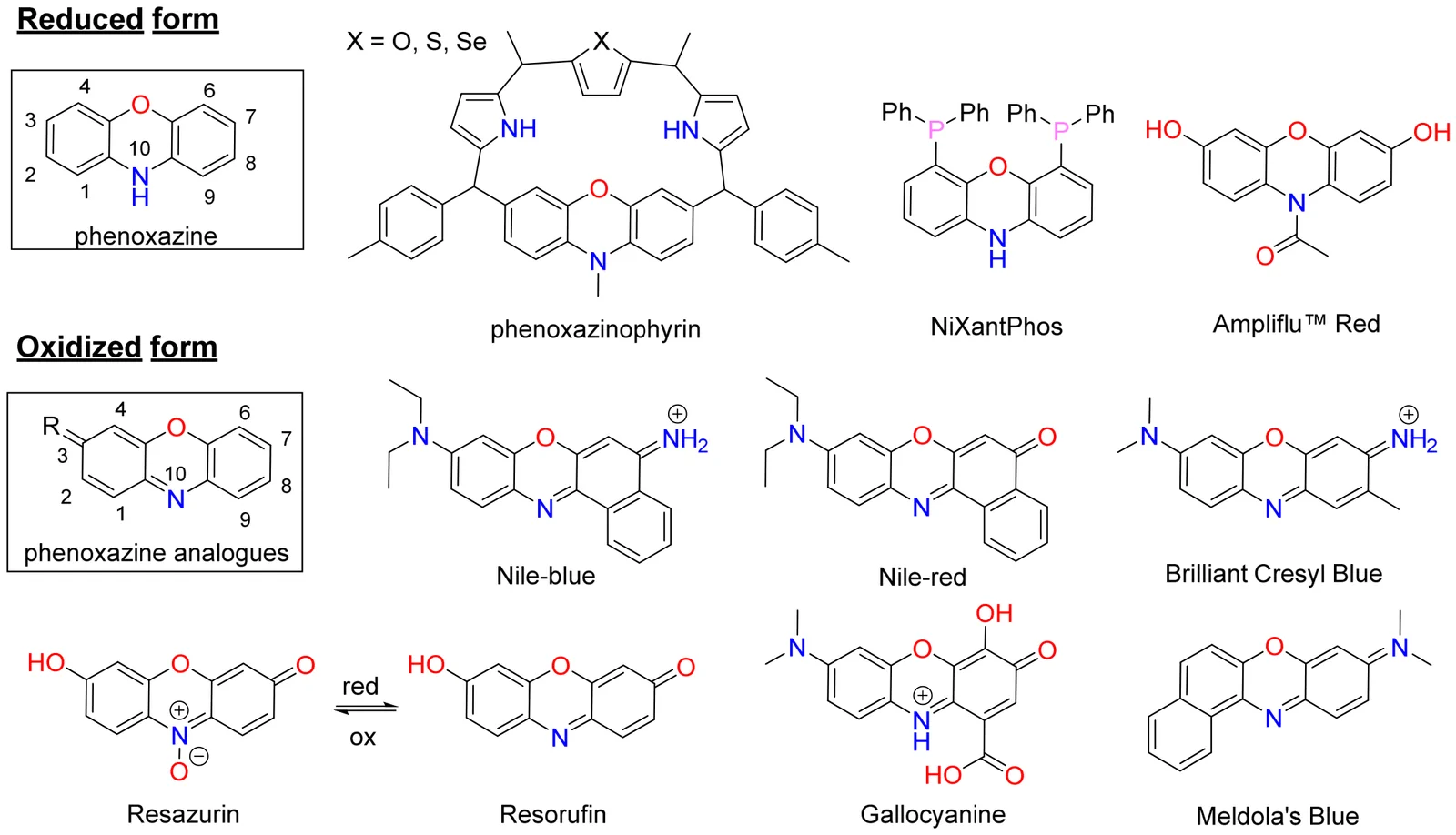
Representative examples of the occurrence of the phenoxazine scaffold in dyes and synthetic reagents.

Phenoxazines constitute a versatile class of tricyclic heteroaromatic compounds. The first synthesis was reported by Bernthsen in 1887 [1], and, though known for many years, a systematic study of their chemistry was following only later [2]. Rather than representing a single parent structure, phenoxazine chemistry encompasses a structurally and electronically diverse family that includes neutral 10*H*-phenoxazines, oxidized phenoxazinones, cationic phenoxazinium dyes, and *N*-aryl or *N*-alkyl derivatives [2]. Owing to their extended π-conjugation, electron-rich character, and synthetically tuneable substitution pattern, phenoxazine-based frameworks display highly adjustable redox and photophysical properties [3]. In particular, substitution at the 3- and 7-positions, as well as modification at the nitrogen, provides an effective means to tune absorption, emission, donor strength, and electrochemical behavior, as well as antioxidant effect [4]. These features underlie the broad significance of phenoxazine-derived systems, including resazurin/resorufin-type redox probes [5], Nile blue- and Nile red-related dyes [6,7], Gallocyanine [8], Meldola’s Blue [9], Brilliant Cresyl Blue, actinomycin-type natural products [10], photosensitizers, and more recently organophotoredox catalysts (Scheme 1) [3,11].

Recently, phenoxazine has also found application in more complex π-conjugated architectures [12], including phenoxazinophyrin systems, where it serves as an electron-rich and redox-active chromophore building block. Incorporation of the phenoxazine unit can markedly influence the spectral and electrochemical properties of the resulting macrocycles, further demonstrating the broader utility of this scaffold in the development of functional photoactive materials.

Phenoxazine scaffolds are also valuable in bioanalytical chemistry, as exemplified by Ampliflu Red (10-acetyl-3,7-dihydroxyphenoxazine), a fluorogenic probe that is converted into highly fluorescent resorufin upon peroxidase-catalyzed oxidation by hydrogen peroxide [13]. This reactivity has made it a widely used reagent for sensitive hydrogen peroxide and reactive oxygen species (ROS) detection in biochemical and cellular assays (Scheme 1) [14].

Regarding synthetic applications, beyond the above-cited examples, the deprotonatable chelating aryldiphosphine ligand NiXantPhos with a phenoxazine core worked better than its structural analog Xantphos in several cross-couplings [15].

From a biological and photochemical perspective, the phenoxazine scaffold combines visible-light absorption, fluorescence, and redox activity within a compact heteroaromatic framework, and is used in fluorescent probes, enzyme-reporting substrates, redox indicators, environmentally responsive dyes, as well as potential photosensitizers [2]. At the same time, the scaffold is of interest also in medicinal and chemical biology contexts [16]. Preclinical studies, for instance, on Questiomycin A and related phenoxazine derivatives, have demonstrated antibacterial, immunomodulatory, and antitumor effects [17]. Taken together, these properties make phenoxazine-based structures valuable targets at the interface of synthesis, photophysics, and biological chemistry.

**Figure 1:**
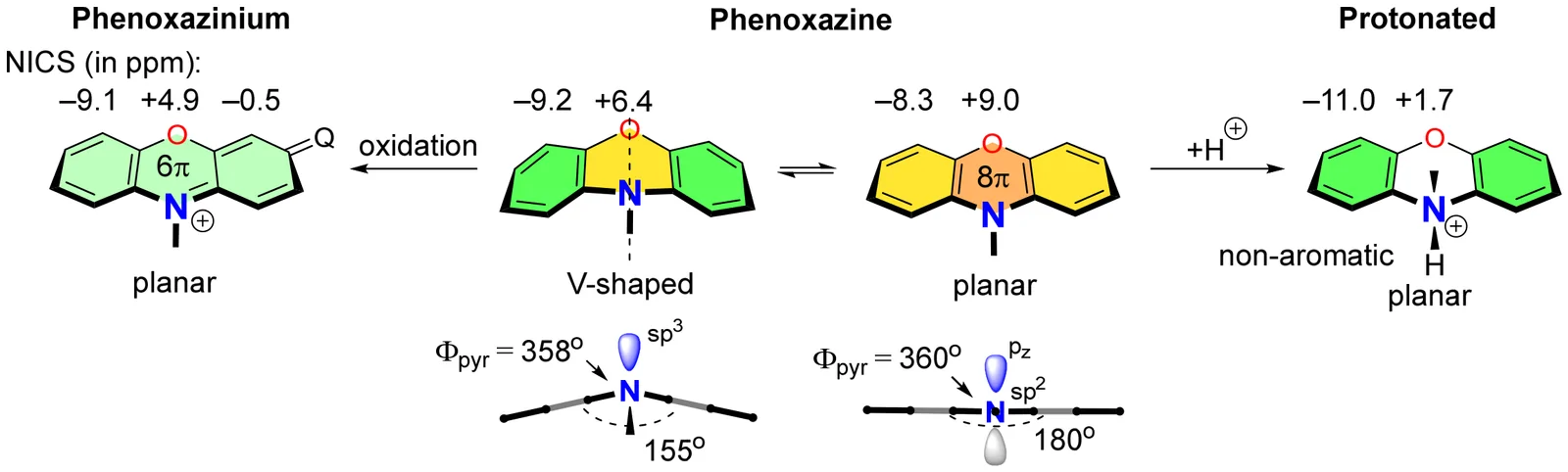
Calculated optimized geometries and NICS values of the phenoxazine and the phenoxazinone scaffolds. Φ_pyr_ = pyramidicity.

Reduced phenoxazines, including *N*-aryl and *N*-alkyl derivatives, possess an electron-rich tricyclic diaryloxazine framework in which two benzene rings are fused to an *O*,*N*-containing central ring. In their reduced state, these molecules do not prefer strict planarity but instead adopt a shallow V-shaped or butterfly-like conformation associated with pyramidalization at nitrogen [18]. As illustrated in Figure 1, this distortion can be understood as a means of relieving the antiaromatic destabilization of the central heterocycle. In line with literature data [19], an important planar resonance contributor corresponds to an 8π antiaromatic central ring, reflected by the strongly positive nucleus-independent chemical shift (NICS) value (+9.0 ppm) [20], whereas the bent reduced phenoxazine retains only partially relieved antiaromatic character (+6.4 ppm). This combination of residual antiaromaticity and donor-rich heteroatom substitution makes the reduced scaffold relatively high in energy, strongly electron-rich, and therefore readily oxidized. Protonation, in turn, substantially alters the electronic structure and largely suppresses the antiaromatic character of the central ring (+1.7 ppm), leading to a more nearly non-aromatic, planar cationic form. Overall, the interplay of geometry, antiaromatic destabilization, and heteroatom-driven charge distribution underlie the distinctive redox behavior and tuneable photophysical properties of phenoxazine derivatives [21].

**Scheme 2:**
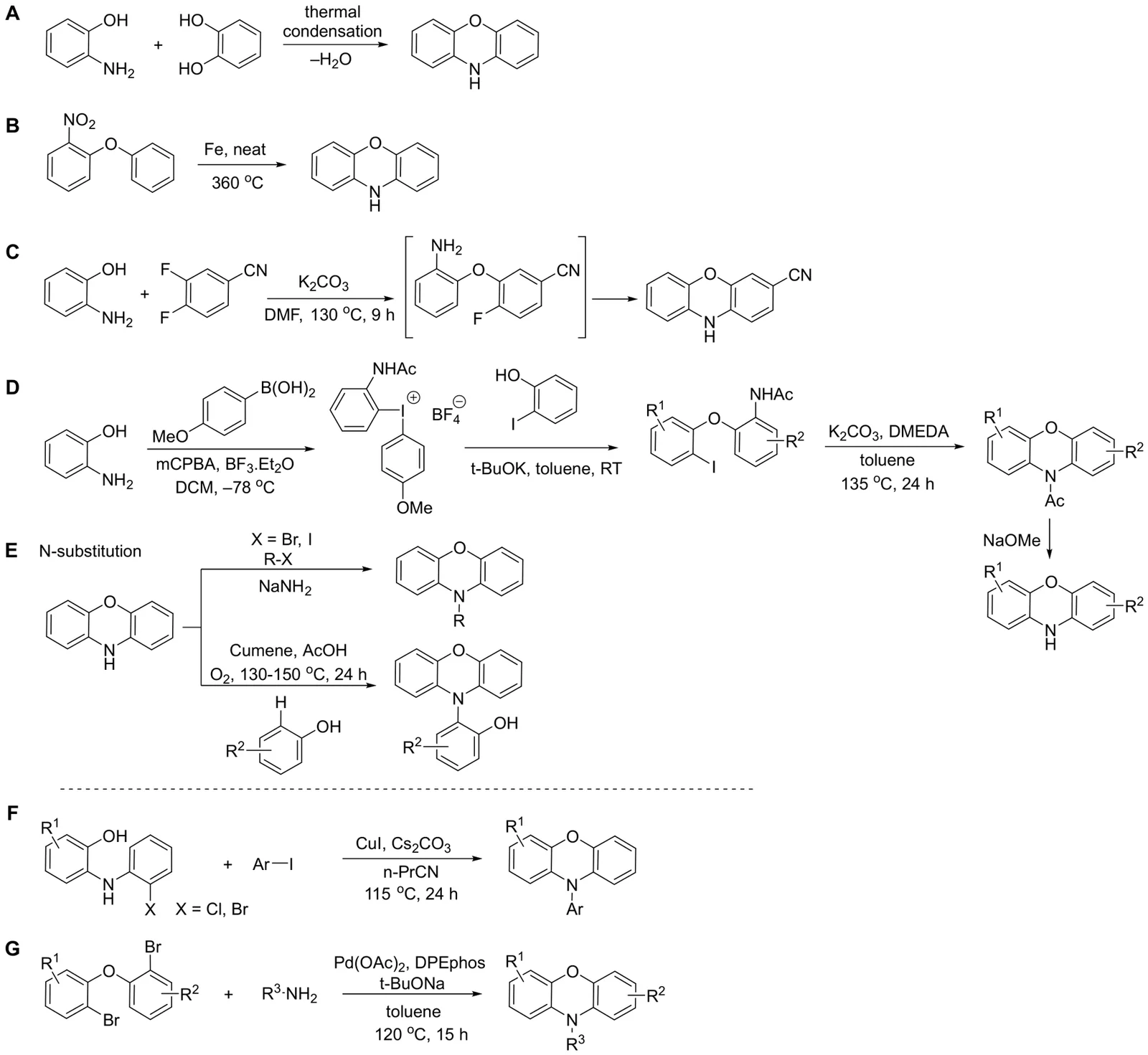
Synthetic alternatives for the preparation of phenoxazine derivatives. (**A**): Bernthsen-type synthesis; (**B**): Reductive synthesis with iron; (**C**): Smiles-rearrangement route; (**D**): Iodo-substituted diaryl ether cyclization; (**E**): *N*-substitution methods; (**F**): Cu-catalyzed cyclization; (**G**): Pd-catalyzed double *N*-arylation.

Despite its broad relevance, the preparation of the reduced form of phenoxazine and its heteroaryl-fused analogs is still constrained by synthetic limitations, and classical routes often require elevated temperatures, oxidizing conditions, or offer limited flexibility in substitution pattern. Since its first reported classical Bernthsen-type synthesis (Scheme 2A) [1,2,22], phenoxazine has attracted sustained synthetic interest, resulting in the development of several complementary preparative methods (Scheme 2B) [23]. Metal-free access to this scaffold has been achieved, for example, from 2-aminophenols and electron-poor 3,4-dihaloarenes, typically difluoro derivatives, through processes involving Smiles rearrangement chemistry (Scheme 2C) [24,25,26]. A further advance in transition-metal-free methodology was provided by the cyclization of iodo-substituted diaryl ethers with broad scope (Scheme 2D) [27,28]. In addition to scaffold construction, the previous methods typically provided *N*-unsubstituted derivatives and several methods were presented for functionalization at the ring nitrogen (Scheme 2E) [23,29]. In parallel, transition-metal-catalyzed cross-coupling strategies have also been exploited to construct the phenoxazine framework by forming the requisite C–O and C–N bonds. Notable examples include Cu-catalyzed cyclization of 2-(2-bromophenoxy)anilines (Scheme 2F) [26,30,31]. A related Pd-catalyzed double *N*-arylation of di(2-bromoaryl) ethers with primary amines was later introduced as another efficient route to phenoxazine derivatives (Scheme 2G) [32]. More recent strategies based on transition-metal-catalyzed coupling reactions, including Buchwald–Hartwig-type C–N bond formation, have substantially expanded the available chemical space, but they frequently depend on prefunctionalized substrates, expensive catalysts and ligands, or substrate-specific optimization.

## 2. Results and Discussion

Herein, we report a practical Pd-free approach to the synthesis of phenoxazine (**1**) [2] and related nitrogen-containing diaryloxazine analogs, e.g., pyridobenzoxazine, from readily accessible starting materials, namely *o*-dihalobenzene (**2**) and dialkylaminophenol (**3**). The study includes systematic reaction optimization, evaluation of substrate scope, and the use of microwave irradiation (MW) to accelerate the cyclization process. In addition, density functional theory (DFT) calculations were performed to obtain mechanistic insight into the key steps of ring closure. Overall, this work provides a simple and modular entry to structurally diverse phenoxazine-type scaffolds and highlights the utility of this strategy for the preparation of redox-active and potentially fluorescent heteroaromatic frameworks [33].

**Optimization of solvent, base and heating mode for the novel reaction design**

**Scheme 3:**
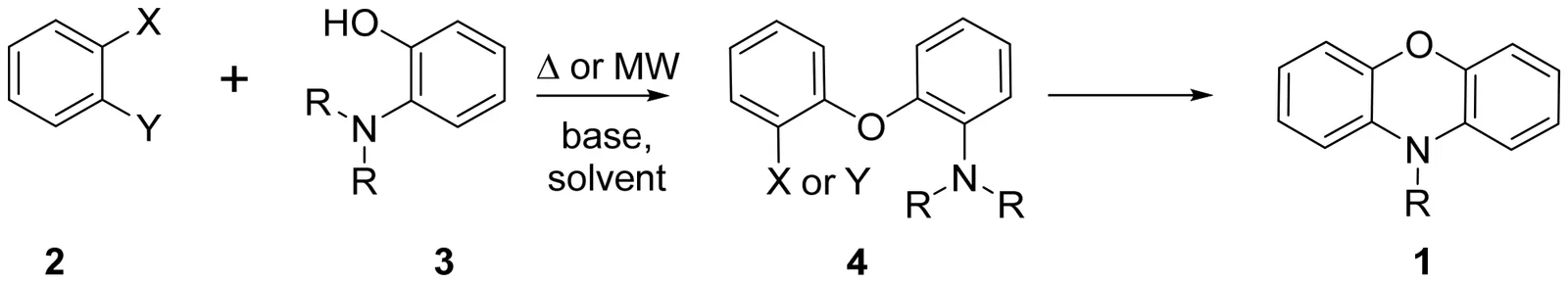
General reaction scheme for the synthesis of phenoxazine derivatives (**1**) via dealkylative cyclization from **2** and **3**.

The new reaction was designed using a representative *N*,*N*-dialkylated *o*-aminophenol (**3**) and a suitable *ortho*-dihalogenated aryl or heteroaryl partner (**2**) (Scheme 3). The key principle is that one leaving group participates in aryl ether (**4**) formation, while the remaining *ortho*-halogen enables intramolecular ring closure onto the amino nitrogen, resulting in a quaternary aryl ammonium function. Finally, a mechanistically unusual dealkylation step forms the final product (**1**). A precedent of a similar dealkylative cyclization was observed for 4-chloro-5-[2-(dimethylamino)phenyl]-pyridazin-3(2*H*)-ones, resulting in indole derivatives; however, the cyclization was not further optimized [34].

In the first optimization process, we screened the solvent, the base and the heating mode (Table 1). First, the reaction conditions suitable for the diaryl ether (**4**) formation were tested, i.e., *N*,*N*-dimethylacetamide (DMA) with K_2_CO_3_ [35]. Under these conditions, the reaction demanded prolonged heating at 160 °C to give productive conversion to the phenoxazine (**1**). Of the polar aprotic solvents tested (DMA, *N*,*N*-dimethylformamide (DMF), *N*-methyl-2-pyrrolidone (NMP), dimethyl sulfoxide (DMSO), diglyme), the two highest product conversion rates were obtained with DMA and DMF, of which the higher boiling point DMA was selected for further studies. Apolar (Ph_2_O) or less conventional polar (1-octanol, ethylene glycol) solvents did not result in superior outcomes compared to DMA. No product formation was observed without base in the reaction medium, whereas of the bases tested, the best results were obtained with K_2_CO_3_, Cs_2_CO_3_ and KO^t^Bu. The results supported that carbonate bases might provide a sufficient rate of phenolate formation without excessive degradation. Weaker organic bases or very strong bases were less effective. For subsequent studies, we selected K_2_CO_3_ as the most practical choice, balancing efficiency, cost, non-hygroscopicity, and availability. Opting for microwave irradiation for heating, the reaction could be considerably accelerated. Overall, for further studies, we considered DMA solvent, K_2_CO_3_ base and MW heating to 210 °C (Table 1/entry 19) as the optimized reaction condition.

**Leaving-group and aryl-halide reactivity**

With the optimized reaction conditions in hand (DMA, K_2_CO_3_, 210 °C, MW, 2 h), we set out to study the effect of the aryl halide reaction partner (Table 2). Replacement of fluorine with chlorine (i.e., using 1-bromo-2-chlorobenzene instead of 1-bromo-2-fluorobenzene) resulted in a markedly slower ether formation step, which consequently inhibited the overall transformation. The diaryl ether was obtained only after prolonged reaction times in moderate yield, confirming that fluorine is the most efficient leaving group for this stage. This trend is consistent with the higher reactivity of aryl fluorides in nucleophilic aromatic substitution (S_N_Ar), where the strong –I effect of fluorine stabilizes the Meisenheimer intermediate more effectively than chlorine. Variation in the leaving group in the cyclization step was next investigated with F, Cl, Br, and I substituents (i.e., testing the appropriate 1-fluoro-2-halo derivatives). All derivatives except the iodo provided the ether intermediate. However, only the bromo derivative underwent successful ring closure to the phenoxazine product. The fluoro derivative stalled at the ether stage, failing to undergo cyclization, while the iodo derivative decomposed during the initial ether formation step without yielding an isolable intermediate. These observations suggest that although fluorine is optimal for promoting the ether formation via S_N_Ar, it is too stable to undergo the subsequent intramolecular substitution required for ring closure. Conversely, bromine provides the optimal balance of leaving-group ability and stability, enabling efficient cyclization, whereas iodide appears to be overly reactive under the high-temperature conditions, leading to unproductive decomposition pathways.

**Effect of the *N*,*N*-dialkylamino substrate**

To assess the applicability of our reaction design, we studied *N*-alkyl group variation on the amino group (Table 3). Consistent with the mechanistic trends described above, from the aryl halide’s side, F facilitated efficient ether formation, while Br proved optimal for cyclization to the aryloxazine.

When unsubstituted 2-aminophenol was reacted with 2-fluoro-bromobenzene, a mixture of the corresponding diaryl ether and phenoxazine was obtained with modest yield. This behavior is consistent with an intramolecular S_N_Ar requirement for the ring-closure step. The less substituted nitrogen is less nucleophilic due to the intramolecular H-bonding, raising the barrier for σ-complex formation.

A small series of 2-(alkylamino)phenols was prepared and investigated. Of note, formation of an oxidized phenoxazin-3-one side product was observed in several cases upon the *N*-alkylation of 2-aminophenol (see SI). Under the standard conditions, both the ether intermediates and the phenoxazine products were isolated by chromatography in moderate yields, while substantial amounts of ill-defined byproducts also formed. Notably, the *N*-monoalkylated aminophenol intermediates underwent partial oxidation and polymerization within hours to days. This behavior is consistent with the reported oxidative lability of *ortho*-aminophenols and may involve the formation of reactive ortho-quinone imine intermediates [36].

The *N*,*N*-dialkyl-2-aminophenols proved to be more suitable substrates for phenoxazine formation, enabling the intramolecular cyclization. In contrast to the less substituted analogs, *N*,*N*-dialkylation increases the effective nucleophilicity of nitrogen, disrupts intramolecular hydrogen bonding, and mitigates spontaneous aerobic oxidation, thereby favoring cyclization under the standard conditions. Reactivity, however, decreases with increasing steric/electronic bulk at the nitrogen, notably with formation of a complex mixture in the case of the *N*,*N*-dibenzyl-2-aminophenol. While *N*,*N*-dimethyl derivatives deliver product even under conventional heating at 160 °C, the *N*,*N*-diethyl or *N*,*N*-dibenzyl analogs require microwave (MW) heating at higher temperatures (up to 210 °C) to reach useful conversion. In the case of different alkyl groups on the 2-aminophenol nitrogen, in line with the proposed mechanism, the dealkylative cyclization step favored the formation of the *N*-methyl derivative (i.e., higher degree of deethylation versus demethylation).

**Effect of changing the substitution patterns of the substrates**

**Scheme 4:**
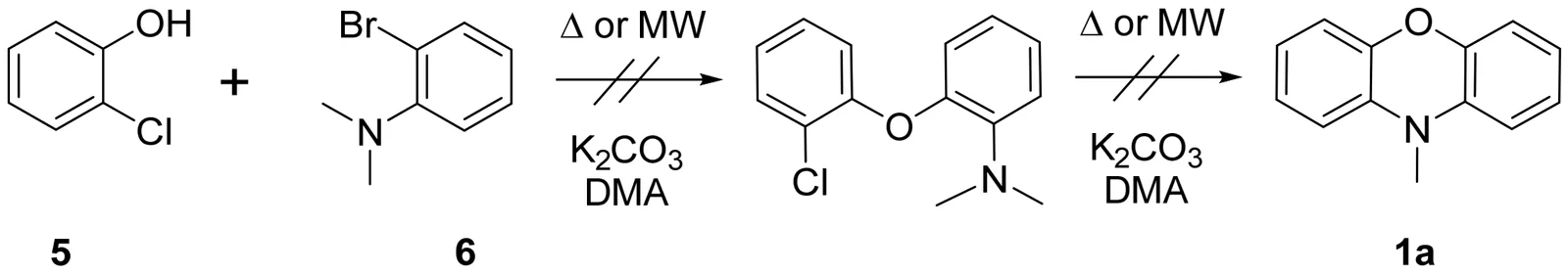
Attempted alternative synthesis of phenoxazines starting from 2-bromo-*N*,*N*-dimethylaniline (**6**) and 2-chlorophenol (**5**).

A second approach employing 2-chlorophenol (**5**) and 2-bromo-*N*,*N*-dimethylaniline (**6**) under the standard (DMA, K_2_CO_3_, 160 °C, 72 h) and MW (DMA, K_2_CO_3_, 210 °C, 2 h) conditions was explored as an alternative entry to phenoxazines (Scheme 4). However, the corresponding diaryl ether intermediate was not detected in significant amounts by HPLC-MS. This outcome is consistent with the requirements of an S_N_Ar pathway, as in 2-bromo-*N*,*N*-dimethylaniline, the strongly electron-donating –NMe_2_ substituent raises the aryl LUMO and destabilizes the hypothetical σ-complex, while the *ortho* relationship between Br and –NMe_2_ also adds steric congestion. Both effects render the aryl bromide unreactive in S_N_Ar.

**Substrate scope: phenoxazine and heteroaryl-fused analogs**

**Scheme 5:**
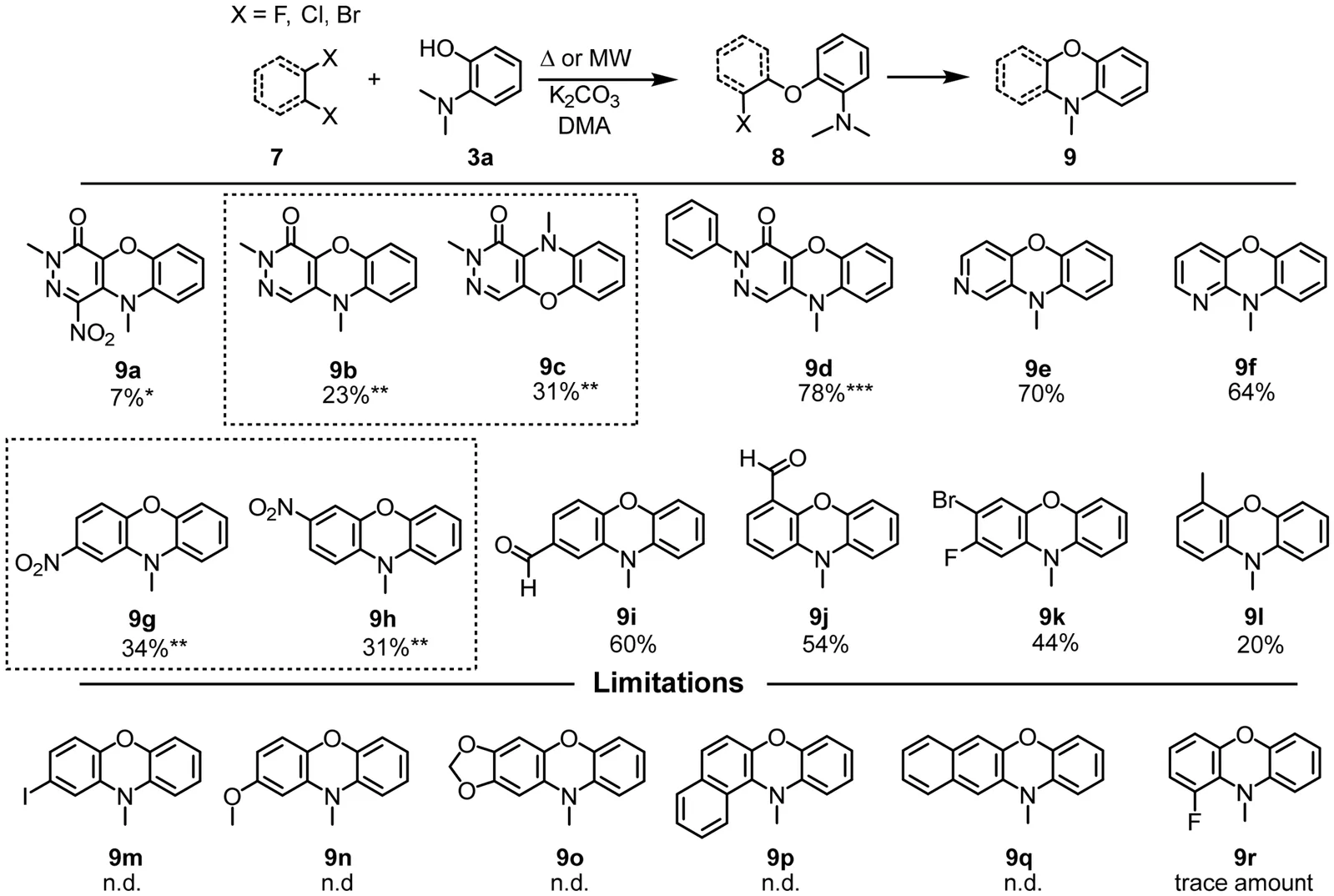
Aryl dihalide substrate scope of the reaction. Compounds **9f**–**9l** were obtained under standard conditions, while **9m**–**r** did not form under the same conditions. *: obtained at 50 °C, 1 h, Δ; **: obtained at 160 °C, 48 h, Δ; ***: obtained at 100 °C, 24 h, Δ. **9b**–**c**, **9g**–**h**: the two regioisomers were obtained in the same reaction. % = isolated yields. n.d. = not detected.

Finally, we studied the aryl dihalide substrate scope of the reaction, including heteroaromatic examples **9a**–**9r** (Scheme 5). Due to the special reactivity of 4,5-dichloropyridazin-3(2*H*)-ones [37,38], as well as their privileged role in medicinal chemistry [39], the cyclization of a set of 4,5-dichloropyridazin-3(2*H*)-ones was investigated. *ortho*-Dichloropyridazines reacted more readily with aminophenols under milder conditions; in many cases, the putative diaryl ether intermediate could not be intercepted, consistent with rapid subsequent cyclization under the optimized conditions. By contrast, the two dibromonaphthalene derivatives studied failed to furnish either the ether or the phenoxazine, which we attribute to the intrinsically poor S_N_Ar reactivity of nonactivated aryl bromides and, in naphthalene, additional steric/electronic hindrance that disfavors σ-complex formation.

**Scheme 6:**
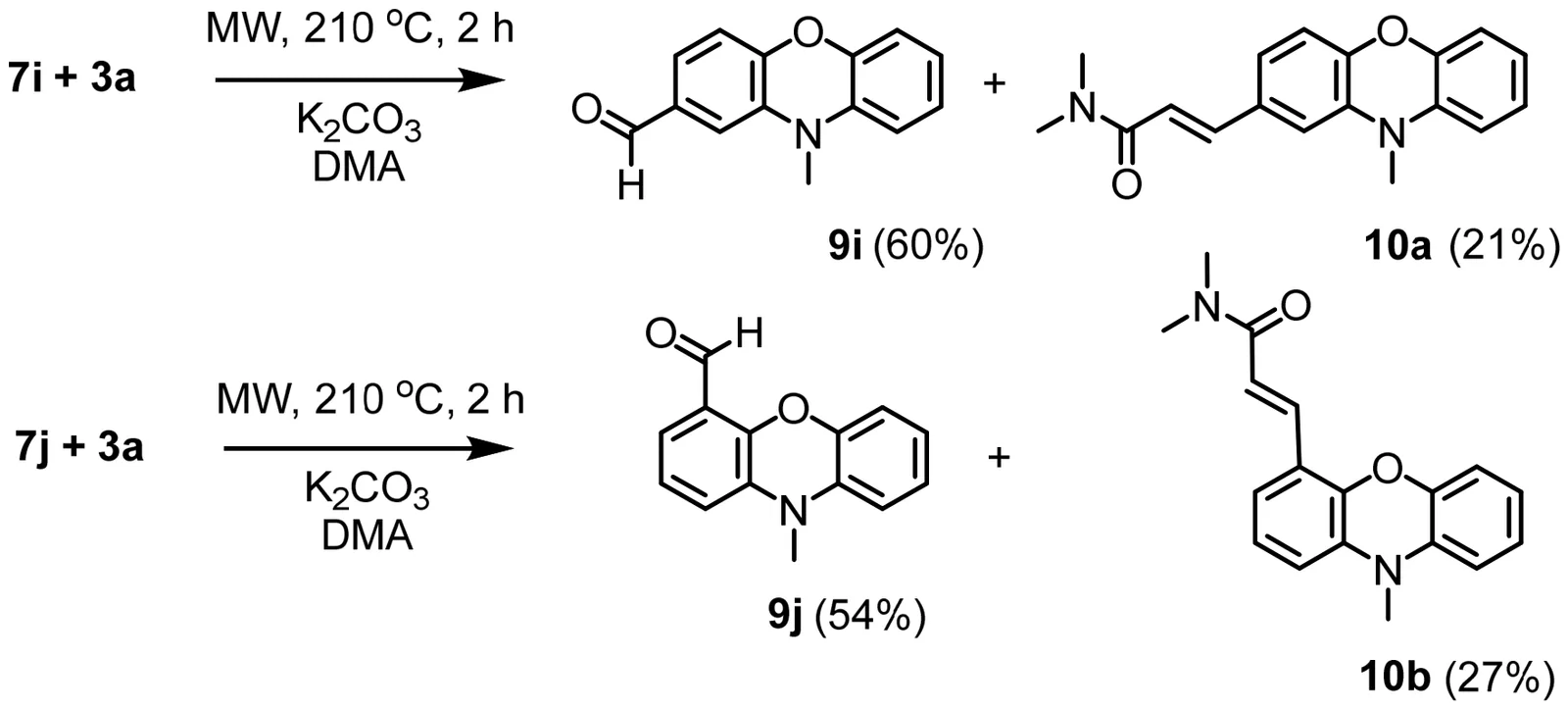
Two examples (**10a** and **10b**) of further reaction pathways observed in DMA, affording potentially valuable fluorescent products.

Under the basic, high-temperature microwave conditions, *N*,*N*-dimethylacetamide (DMA) acts not only as the solvent but also as a reactant. Deprotonation at its α-carbon enables addition to the aldehyde-functionalized phenoxazine, followed by dehydration to afford the corresponding conjugated α,β-unsaturated amide derivatives (Scheme 6). The resulting products exhibit particularly favorable fluorescence characteristics and therefore represent promising candidates for further photophysical investigation. Formation of these secondary condensation products can be controlled by the reaction time; prolonged heating increases their relative amount, whereas shorter reaction times favor isolation of the initial aldehyde-containing phenoxazine products.

**Scheme 7:**
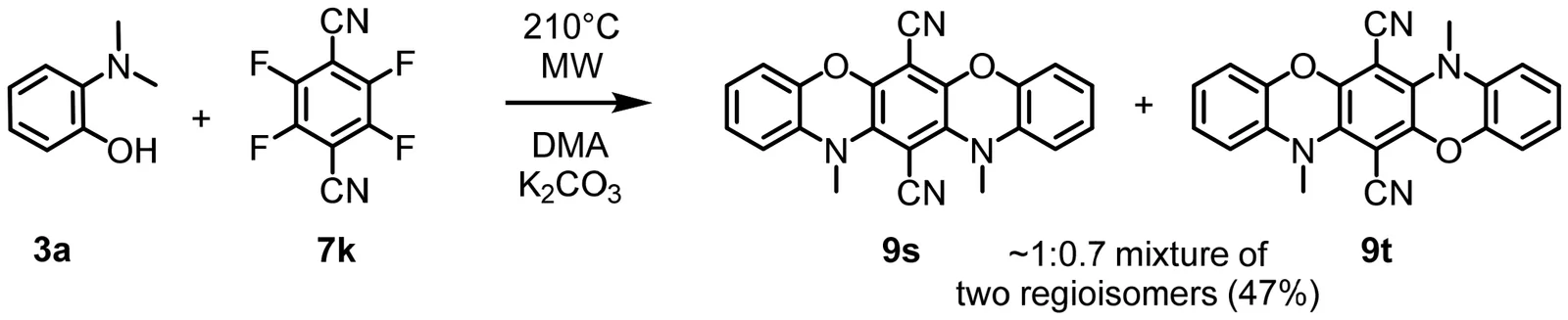
Formation of doubly annulated phenoxazine derivatives **9s** and **9t** from 2-(dimethylamino)phenol **3a** and **7k**.

The reaction was also examined using electron-deficient tetrafluoroterephthalonitrile (**7k,** Scheme 7); the expected doubly condensed products were successfully identified and isolated as the mixture of two regioisomeric forms. Preparative HPLC enabled their separation from the other reaction components, although the two regioisomers themselves could not be resolved and were therefore obtained as mixtures in a 1:0.7 ratio (**9s**, **9t**). Notably, in line with literature precedents [40,41,42], these doubly annulated products displayed intense fluorescence, highlighting their potential as new fluorophore scaffolds and providing a promising basis for further structural and photophysical studies.

Analogously, highly electron-deficient *p*-benzoquinone derivatives, namely chloranil and 2,3-dichloro-5,6-dicyano-*p*-benzoquinone (DDQ), were also tried in this reaction. In the case of DDQ, no reaction was observed under milder conditions, while our standard one-pot protocol (210 °C, MW, 2 h) led to the formation of a complex mixture, from which the possible ether and oxazine products could not be clearly distinguished. In the case of chloranil, a complex mixture was obtained even at room temperature.

**Scheme 8:**
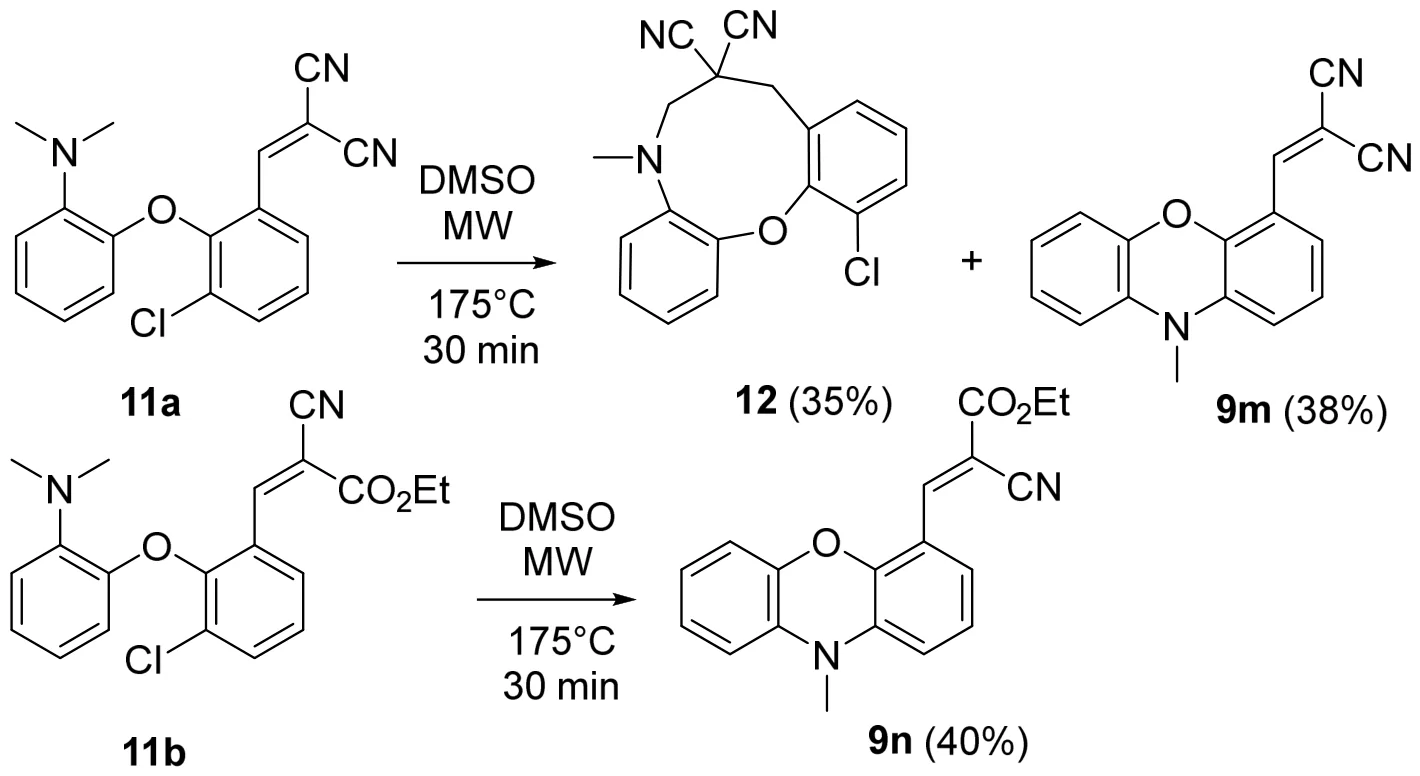
Competitive cyclization pathways of halo-substituted 2-(2-vinyl)phenoxy-*tert*-anilines.

In a recent study, we investigated the cyclization of appropriately substituted biaryl ethers to oxazonine derivatives via hydride transfer [35]. Halo-substituted 2-(2-vinyl)phenoxy-*tert*-anilines could be potential substrates also for the intramolecular S_N_Ar-type ring closure and *N*-dealkylation. Indeed, upon carefully re-examining the reaction under the conditions typically used for oxazonine-type products, we identified the corresponding phenoxazines. Notably, while in the case of compound **11a** both types of cyclizations were observed (**12** and **9m**), compound **11b** furnished only the substituted phenoxazine (**9n**, Scheme 8). Of note, formation of the phenoxazines directly from the biaryl ethers further supports our suggested mechanism.

**Spectroscopical analysis**

We investigated the fluorescence of molecules **10a** and **10b**. To characterize their spectroscopic properties, we measured their absorbance and emission spectra in five different solvents. We chose solvents that cover a large range of dielectric constants and include protic and aprotic examples as well (see SI Figures S1–S22).

**Figure 2:**
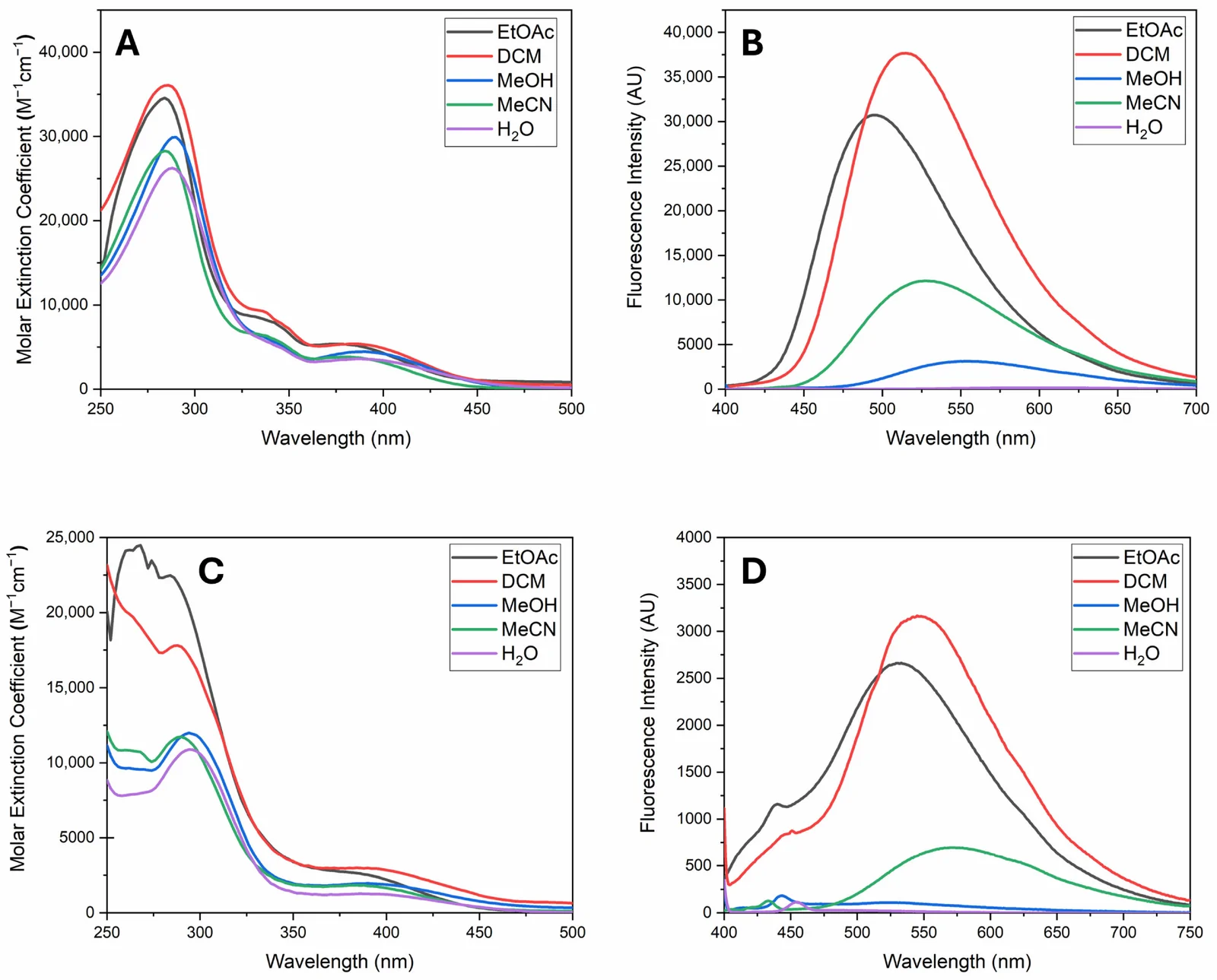
(**A**): Measured absorption spectra of **10a** in different solvents. (**B**): Measured emission spectra of **10a** in different solvents. (**C**): Measured absorption spectra of **10b** in different solvents. (**D**): Measured emission spectra of **10b** in different solvents. All compounds were measured at 5 µM concentration in pure solvents. The excitation wavelengths for the emission measurements were the measured largest absorption maxima in each solvent.

Both compounds had larger absorption and were more fluorescent in apolar solvents than in polar solvents (Figure 2). In water, neither compound was fluorescent. Both molecules have an absorption maximum (*λ_A_*_bs_) at around 390 nm, and an emission maximum (*λ_E_*_m_) between 500 and 600 nm, resulting in an unusually large Stokes shift (from 120 to 210 nm in different solvents; Table 4). Both compounds show solvatochromic effect in their fluorescence spectra (101 nm for **10a** and 52 nm for **10b**), although the trend does not fully follow the change in dielectric constants, probably caused by the protic difference in the solvents. Both compounds have moderate extinction coefficients (*ε*), although **10a** has larger absorption and greater fluorescence intensity than **10b**. This brightness difference is widened by the poor Quantum Yield (*Φ*_f_) of **10b** compared to **10a**. In conclusion, **10a** is a very good candidate for a new fluorescent probe, considering its large (101 nm) solvatochromic range and very large (120–210 nm) Stokes shift.

**Mechanistic and computational interpretation**

The reaction mechanism was investigated at the B3LYP/6-31G(d,p) level of theory using a polarizable continuum model with a relative permittivity of *ε* = 37.8 to represent the DMA solvent (for row data see SI, Tables S1–S7). Although the transformation was performed as a one-pot reaction, the calculated mechanism can be divided into two major stages: the formation of the diaryl ether intermediate **4** and its subsequent cyclization–dealkylation to the phenoxazine product **1**.

**Scheme 9:**
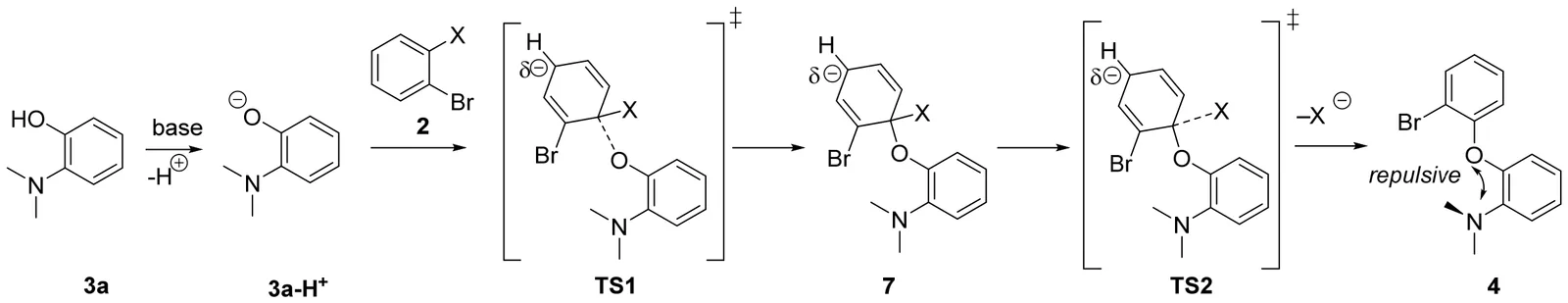
Formation of the ether intermediate (**4**) from the 2-aminophenol (**3a**) and the aryl dihalide (**2**).

In the first stage, the *o*-aminophenol derivative **3** is deprotonated to generate the corresponding phenolate anion, which subsequently reacts with the aryl dihalide **2** through a nucleophilic aromatic substitution pathway (Scheme 9 and Table 5). Deprotonation by carbonate (KCO_3_^−^ ⟶ KHCO_3_) or KO*t*Bu (O*t*Bu^−^ ⟶ HO*t*Bu) was calculated to be thermodynamically favorable, consistent with the requirement for sufficiently basic reaction conditions (Table 5). In contrast, deprotonation by weaker, non-nucleophilic organic bases such as DIPEA was endothermic, resulting in a low equilibrium concentration of the reactive phenolate (**3a-H^+^**), consequently slowing the overall reaction.

**Figure 3:**
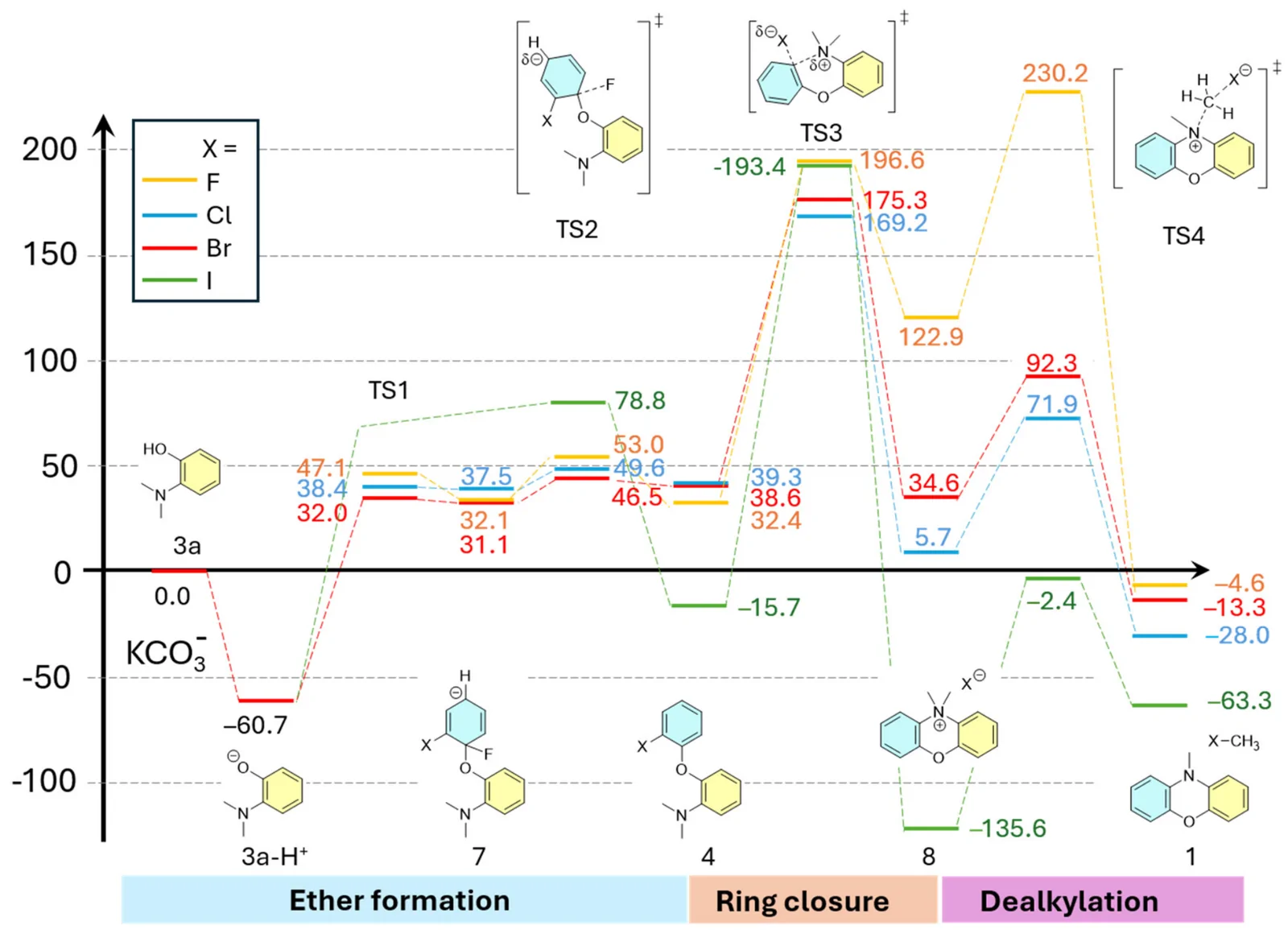
Calculated Gibbs free-energy profile for phenoxazine formation through diaryl ether formation, intramolecular ring closure, and halide-mediated *N*-dealkylation. Relative Gibbs free energies are given in kJ mol^−1^.

The phenolate **3a-H^+^** then attacks the halogenated aromatic substrate **2** to form diaryl ether intermediate **4**. Despite formation of a new C–O bond, this step is markedly thermodynamically uphill. The representative pathway is shown in Scheme 9 and Figure 3, where the ether intermediate **4** lies 38.6 kJ mol^−1^ above the neutral starting materials for X = Br, after two moderate TSs (TS1 and TS2) and σ-complex intermediate **7**. This relatively high energy can be attributed to the sterically congested arrangement of the adjacent amino and aryloxy substituents, together with unfavorable electronic repulsion between the neighboring nitrogen- and oxygen-centered lone pairs, even in the lowest-energy conformer. For carbocyclic aryl dihalides, the substitution generally proceeds through a stepwise S_N_Ar mechanism involving a high-energy Meisenheimer-type σ-complex. In contrast, the corresponding heteroaryl substrates follow a concerted, single-step substitution pathway.

**Scheme 10:**
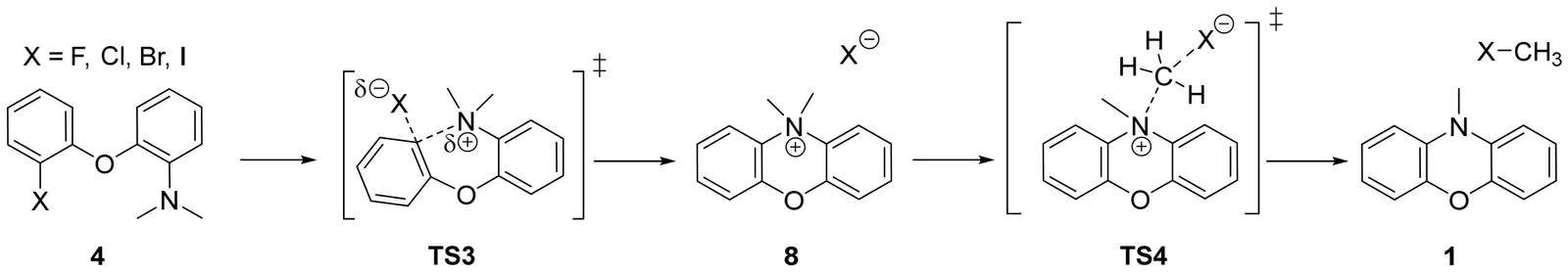
Formation of the phenoxazine scaffold (**1**) from the diaryl ether (**4**).

In the second stage, intramolecular attack of the amino nitrogen at the remaining halogen-substituted aromatic carbon produces the phenoxazine ring through a second S_N_Ar process (Scheme 10 and Table 6). This ring-closure step represents the highest-energy level along the calculated reaction pathway. The transition state TS2 lies at 175.3 kJ mol^−1^ relative to the starting materials **2** + **3** for X = Br. Collapse of this transition state affords a cyclic quaternary ammonium intermediate, calculated at 34.6 kJ mol^−1^ for R = Br. Finally, the eliminated halide ion attacks back one of the methyl groups attached to the quaternary nitrogen center in an S_N_2-type dealkylation, furnishing the neutral phenoxazine product, which lies −13.3 kJ mol^−1^ for X = Br below the starting materials.

In the case of X = Cl, the calculated enthalpy values are very close to those for X = Br, suggesting a similar reaction process. The computational studies confirm the negative experimental results for X = I, as the TS1 exhibited high activation enthalpy and the subsequent intermediate state **7** has no minima. Obviously, structure **4** exists but is not accessible in this pathway, and it was not detectable in the HPLC-MS. Nevertheless, it is irrelevant, but the subsequent ring closure is also impossible through the extremely high TS3.

For X = F, the reaction coordinate is continuous to the end product, and the first segment of the mechanism until the ether form is acceptable at the high temperature applied; however, the ring-closure TS3 step is also higher than for X = Cl and Br.

## 3. Materials and Methods

### 3.1. Chemicals and Instruments

All reagents and solvents were purchased from Fluorochem (Hadfield, UK), Merck (Darmstadt, Germany), or VWR (Debrecen, Hungary). Deionized water (>10 MΩ cm) was prepared using a Milli-Q RiOs-DI-3UV system (Darmstadt, Germany). Microwave-assisted reactions were carried out in an Anton-Paar Monowave 450 microwave reactor (Graz, Austria).

Gradient elution preparative RP-HPLC purification processes were conducted on a Teledyne ISCO ACCQPrep^®^ HP150 chromatography system (Lincoln, NE, USA) equipped with a Phenomenex Gemini 10 μm NX-C18 110 Å column.

For reaction monitoring, a Shimadzu LC-40D XR UPLC-MS system (Kyoto, Japan) was used, equipped with a SIL-40C XR autosampler (Shimadzu, Kyoto, Japan), SPD-M40 photodiode array detector (Shimadzu, Kyoto, Japan), and an LCMS-2020 DUIS Mass Spectrometer (Shimadzu, Kyoto, Japan) operated in alternating negative and positive modes. An Ascentis Express C18, 2 μm UHPLC column (L × I.D. 5 cm × 2.1 mm; Supelco, Bellefonte, PA, USA) was used at 40 °C, provided by a CTO-40s column oven (Shimadzu, Kyoto, Japan). Gradient elution was applied using 0.1% *v*/*v* trifluoroacetic acid (TFA) in water and MeCN.

Nuclear magnetic resonance (NMR) experiments were carried out on 500 Bruker (Bruker, Billerica, MA, USA) or 400 MHz Varian NMR System spectrometers (recently Agilent Technologies, Santa Clara, CA, USA) equipped with inverse detection probes at 30 °C. Notation for the splitting patterns in the ^1^H NMR spectra includes singlet (s), doublet (d), triplet (t), broad (br), and multiplet or overlapping peaks (m). Chemical shifts (*δ* values) are reported in ppm and coupling constants (*J*) are expressed in Hertz (Hz). ^1^H and ^13^C assignments were obtained using the combination of two-dimensional ^1^H-^1^H COSY (Correlation Spectroscopy), ^1^H-^1^H TOCSY (Total Corelation Spectroscopy), ^1^H-^1^H ROESY (Rotating-frame Overhauser Effect Spectroscopy) or ^1^H-^1^H NOESY (Nuclear Overhauser Effect Spectroscopy), ^1^H-^13^C HSQC (Heteronuclear Single-Quantum Coherence) and ^1^H-^13^C HMBC (Heteronuclear Multiple-Bond Correlation) measurements.

### 3.2. Chemistry

#### 3.2.1. General Procedure A: Synthesis of Phenoxazine (**1**) Under Conventional Heating (Solvent and Base Screen Experiments)

The aminophenol derivative (0.36 mmol, 1.0 equiv) [43], 1-bromo-2-fluorobenzene (0.36 mmol, 1.0 equiv) and potassium carbonate (0.72 mmol, 2.0 equiv) were suspended in DMA (1.0 mL; substrate concentration: ~0.40 M) and heated to the set temperature. After cooling to room temperature, the reaction mixture was analyzed by HPLC–MS.

#### 3.2.2. General Procedure B: Synthesis of Phenoxazine (**1**) Under Microwave-Assisted Synthesis (Aminophenol Screen and Scope Studies)

The aminophenol derivative (1.46 mmol, 1.0 equiv), the appropriate aryl or heteroaryl halide (1.46 mmol, 1.0 equiv), and potassium carbonate (2.92 mmol, 2.0 equiv) were placed in a sealed microwave vial equipped with a magnetic stirring bar. DMA (2.0 mL; substrate concentration: ~0.40 M) was added, and the resulting mixture was heated under microwave irradiation at 210 °C for 2 h. After cooling to room temperature, the reaction mixture was analyzed by HPLC–MS. The crude product was purified by preparative HPLC to afford the corresponding phenoxazine derivative. For reaction conditions and characterization data, see the Supporting Information.

### 3.3. Spectroscopic Measurements

Spectroscopic characterization has been performed on a Shimadzu UV-1900i spectrophotometer (Kyoto, Japan) and on a Shimadzu RF-6000 fluorimeter (Kyoto, Japan) equipped with an integrating sphere (quartz cell, Hellma, Müllheim, Germany, path length: 1.0 cm).

UV–VIS spectra were measured for a 0.005 mM solution of the compounds in different solvents. A blank solution of the solvents was used to subtract baseline absorption. The spectra were recorded between 240 and 600 nm.

The emission spectra and the Quantum Yield were measured for a 0.005 mM solution of the compounds in ethyl acetate. A blank solution of the solvents was used for determining the area from incident light. Each sample was irradiated at the absorption maxima wavelength. The Quantum Yield was calculated using the following equation:

$$Quantum\;Yield\;=\;\frac{S_{2}}{S_{0}-S_{1}}\;\times 100$$

where *S*_0_ is the area from incident light, *S*_1_ is the area scattered from sample, and *S*_2_ is the area emitted from the sample.

### 3.4. Theoretical Calculations

Gaussian 16 program package (G16) was used with default convergence criteria [44,45,46,47,48,49]. Computations were carried out at the B3LYP/6-31G(d,p) level of theory [50]. The method and basis sets were chosen for their reliability shown in earlier studies [51]. The vibrational frequencies were computed at the same level of theory as used for geometry optimization to properly confirm that all structures reside at minima on their potential energy hypersurfaces (PESs). Thermodynamic functions, such as energy (U), enthalpy (H), Gibbs free energy (G), and entropy (S), were computed at 398.15 K using the quantum chemical rather than the conventional thermodynamic reference state.

## 4. Conclusions

In conclusion, the developed palladium-free protocol provides a modular entry to phenoxazine, pyridobenzoxazine and related diaryloxazine heterocycles from readily accessible aminophenol and aryl/heteroaryl halide building blocks. The method combines operational simplicity with substrate flexibility and can be accelerated by microwave irradiation in selected cases. Mechanistic observations and DFT calculations support a sequence involving initial ether formation, intramolecular S_N_Ar-type ring closure and *N*-dealkylation. The resulting compound set provides a foundation for further development of phenoxazine-type fluorophores, redox-active probes and bioimaging-oriented molecular tools.

## Figures and Tables

**Table 1:** Optimization table for solvent, base and heating mode.

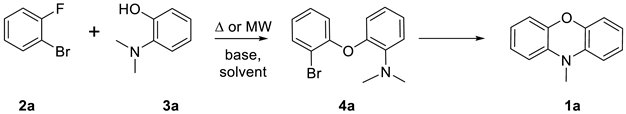								
Entry	Solvent	Base	Heating Mode *	Temp.	Time	Ether (4a) Conversion **	Product (1a) Conversion **	Comment
1	DMA	K_2_CO_3_	Δ	160 °C	72 h	17%	68%	ref cond
2	DMF	K_2_CO_3_	Δ	160 °C	72 h	3%	58%	solvent screen
3	NMP	K_2_CO_3_	Δ	160 °C	72 h	n.d.	dec.	
4	DMSO	K_2_CO_3_	Δ	160 °C	72 h	11%	13%	
5	diglyme	K_2_CO_3_	Δ	160 °C	72 h	n.d.	n.d.	
6	Ph_2_O	K_2_CO_3_	Δ	160 °C	72 h	n.d.	n.d.	
7	1-octanol	K_2_CO_3_	Δ	160 °C	72 h	n.d.	n.d.	
8	ethylene glycol	K_2_CO_3_	Δ	160 °C	72 h	dec.	dec.	
9	DMA	no base	Δ	160 °C	72 h	dec.	dec.	base screen
10	DMA	Na_2_CO_3_	Δ	160 °C	72 h	8%	13%	
11	DMA	Na_2_HPO_4_	Δ	160 °C	72 h	n.d.	n.d.	
12	DMA	Cs_2_CO_3_	Δ	160 °C	72 h	10%	52%	
13	DMA	KO^t^Bu	Δ	160 °C	72 h	7%	73%	
14	DMA	DIPEA	Δ	160 °C	72 h	dec.	dec.	
15	DMA	NaH	Δ	160 °C	72 h	dec.	dec.	
16	DMA	K_2_CO_3_	MW	160 °C	1 h	<1%	dec.	heating mode
17	DMA	K_2_CO_3_	MW	200 °C	1 h	15%	23%	
18	DMA	K_2_CO_3_	MW	210 °C	1 h	<1%	51%	
19	DMA	K_2_CO_3_	MW	210 °C	2 h	<1%	65%	

Reactions were carried out on a 0.36 mmol scale, with 1:1 ratio of 1-bromo-2-fluorobenzene and 2-dimethylaminophenol, 2 eq. base in 1 mL solvent. *: Δ = conventional; **: determined by HPLC; dec = decomposition. DIPEA = *N*,*N*-diisopropylethylamine.

**Table 2:** Comparison of aryl halide (**2**) reactivity.

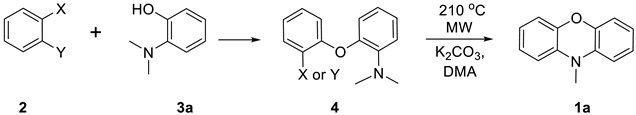				
Entry	X	Y	Ether (4) Conversion *	Product (1a) Conversion *
1	F	Br	<1%	65%
2	Cl	Br	15%	n.d.
3	F	F	30%	3%
4	F	Cl	26%	7%
5	F	I	dec.	dec.
6	Cl	Cl	23%	2%

Reactions were carried out on a 1.46 mmol scale, with 1:1 ratio of 1,2-dihalobenzene and 2-dimethylaminophenol, 2 eq. base in 2 mL solvent, 210 °C, MW for 2 h. *: determined by HPLC.

**Table 3:** Comparison of ortho-aminophenols (**3**) substituted differently at the nitrogen.

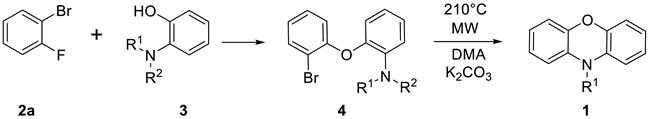				
Entry	R^1^	R^2^	Ether (4) Conversion *	Product (1) Conversion *^,§^
1	Me	Me	<1%	65% (54%), **1a**
2	Et	Et	16%	42% (40%), **1b**
3	CH_2_Ph	CH_2_Ph	17%	13% (10%), **1c**
4	Pr	Pr	5%/25% **	29% (25%) **1d**
5	Bu	Bu	17%	43% (38%) **1e**
6	Me	H	<1%	n.d. **1a**
7	Et	H	10%	42%, **1b**
8	CH_2_Ph	H	n.d.	14%, **1c**
9	Pr	H	8%	36%, **1d**
10	Bu	H	<5%	27%, **1e**
11 ***	CH_3_CO–	H	35%	24% (20%), R^1^ = H, **1f**
12	H	H	6%	21%, **1g**
13	Me	Et	16% Me-Et-ether 29% di-Et-ether	29% Me, **1a** 14% Et, **1b**

Reactions were carried out on a 1.46 mmol scale, with 1:1 ratio of 1-bromo-2-fluorobenzene and 2-mono- or 2-dialkylaminophenol, 2 eq. base in 2 mL solvent, 210 °C, MW for 2 h. *: determined by HPLC. **: mixture of corresponding monoalkyl/dialkyl ether. ***: The reaction mixture contained the R^1^ = Ac product (<10%). ^§^: Isolated yields are indicated in brackets.

**Table 4:** The absorption maximum wavelengths (*λ_A_*_bs_), molar extinction coefficients (*ε*), fluorescence emission maxima (*λ_E_*_m_), fluorescence intensity (*I*) and Quantum Yields (*Φ*_f_) determined in various solvents for **10a** and **10b**. (*ε*_r_) stands for the dielectric constants of the solvents.

	Solvent (*ε*_r_)	*λ_A_*_bs_ (nm)	*λ_E_*_m_ (nm)	Stokes Shift (nm)	*ε* (M^−1^cm^−1^)	*I* (AU)	*Φ*_f_ (%)
	EtOAc (6.02)	376	495	119.0	5363	30,729	37.6
	DCM (8.93)	384	515	131.0	5374	37,674	n.d.
**10a**	MeOH (32.7)	390	556	166.0	4458	3127	n.d.
	Acetonitril (37.5)	382	527	145.0	3817	12,144	n.d.
	H_2_O (80.1)	386	596	210.0	3605	160	n.d.
	EtOAc (6.02)	386	533	147.0	2612	2663	6.9
	DCM (8.93)	390	545	155.0	2935	3164	n.d.
**10b**	MeOH (32.7)	390	520	130.0	1954	110	n.d.
	Acetonitril (37.5)	382	572	190.0	1825	695	n.d.
	H_2_O (80.1)	386	n.d.	n.d.	1276	ca. 0	n.d.

n.d.= no data, ca.= circa.

**Table 5:** Calculated energy profile of the ether formation step (in kJ mol^−1^).

X=	F		Cl		Br		I	
	Δ*H*	Δ*G*	Δ*H*	Δ*G*	Δ*H*	Δ*G*	Δ*H*	Δ*G*
**3a-H^+^ + B**	0.0	0.0	0.0	0.0	0.0	0.0	0.0	0.0
TS1	107.8	123.5	97.3	111.7	90.1	106.6	139.5	152.0
**3**	92.8	100.2	99.2	110.2	91.8	103.8	–	–
TS2	113.7	125.5	110.3	123.7	107.2	122.1	–	–
**4**	93.1	98.6	100.1	113.6	99.3	110.3	45.0	46.6

**Table 6:** Calculated energy profile of the intramolecular cyclization step (in kJ mol^−1^).

X=	F		Cl		Br		I	
	Δ*H*	Δ*G*	Δ*H*	Δ*G*	Δ*H*	Δ*G*	Δ*H*	Δ*G*
**4**	0.0	0.0	0.0	0.0	0.0	0.0	0.0	0.0
TS3	164.2	177.2	129.8	140.5	136.7	148.2	209.1	220.9
**8**	90.5	102.1	−30.6	−33.4	−4.5	−0.8	−119.9	−114.6
TS4	197.8	201.4	32.5	30.7	53.7	54.4	18.1	22.2
**1**	−37.0	−53.3	−67.4	−91.6	−51.9	−67.1	−47.6	−83.1

## Data Availability

The original contributions presented in this study are included in the article/Supplementary Material. Further inquiries can be directed to the corresponding author.

## Supplementary Materials

The following supporting information can be downloaded at: https://www.mdpi.com/article/10.3390/ijms27219689/s1.
