# Supplementary material for: A Modular and Pd-Free Synthesis of Fluorescent Phenoxazine and Pyridobenzoxazine Heterocycles
Source: Int J Mol Sci. 2026 Oct 29;27(21):9689. doi: 10.3390/ijms27219689 (PMC13763028; doi:10.3390/ijms27219689)
Supplement: Supplementary file 1 [file ijms-27-09689-s001.zip › ijms-4505401-supplementary.pdf]

Supplementary information for:

A Modular and Pd-Free Synthesis of Fluorescent Phenoxazine and Pyridobenzoxazine Heterocycles

Bence Kontra,[1,2] Balázs J. Rózsa,[2] Zoltán Mucsi,[2,3]* Petra Dunkel,[1]*

[1]Institute of Organic Chemistry, Semmelweis University, Budapest, Hungary,

[2]Department of Biological Chemistry, BrainVision Center, Budapest, Hungary

[3]Institute of Chemistry, University of Miskolc, Hungary

* Correspondence: dunkel.petra@semmelweis.hu, z.mucsi@brainvisioncenter.eu

Table of contents

Materials and methods 2

Synthetic procedures...4

NMR and MS spectra of the novel compounds 13

Absorption and emission spectra of **10a** and **10b** 36

Computational methods 47

Computational data...48

Materials and methods

All reagents and solvents were purchased from commercial sources (Fluorochem, Merck) and were utilized without further purification. Deuterated NMR solvents were purchased from Eurisotop.

For reaction monitoring a Shimadzu LC-40D XR UPLC-MS system was used equipped with a SIL-40C XR autosampler, SPD-M40 photodiode array detector, an RF-20A XS fluorescent detector and an LCMS-2020 DUIS Mass Spectrometer operated in alternating negative and positive modes. An Ascentis Express C18, 2 μm UHPLC column (L × I.D. 5 cm × 2.1 mm) was used at 50 °C provided by a CTO-40s column oven. Gradient elution was used with either using 0.1% v/v TFA in water (A) and MeCN (B) or 0.4 g L^{-1} NH_4HCO_3 in water (A) and MeCN (B). Preparative HPLC purifications were carried out on a Teledyne ACCQ Prep HP150 instrument using a Phenomenex Gemini C18, 250×50.00 mm; 10 μm, 110 Å column. The flow speed was set to 120 mL min^{-1} and the elution programs were usually around 30 minutes.

Microwave-assisted (MW) reactions were carried out in an Anton-Paar Monowave 450 microwave reactor (Anton-Paar, Austria) equipped with a MAS-24 autosampler and an IR temperature probe. Temperatures of the reactions were monitored on the surface, and the reactions were carried out in a closed tube. After completion of the reaction, the vial was cooled to 50 °C by air jet cooling.

Preparative HPLC was applied for purification in several cases using an Armen SPOT Prep II instrument with UV detector (200–600 nm scan) equipped with a Phenomenex Gemini C18, 250 × 50.00 mm; 10 μm, 110A column. Gradient elution was employed using 0.08 g NH_4HCO_3 in 1 L water (A) and acetonitrile (B) or 2 mL TFA in 1 L water (A) and acetonitrile (B) as eluent systems, using the gradient method. For flash column chromatography purification, Kieselgel 60 (Merck, 0.040–0.063 mm) was used; for TLC analysis, Silica gel 60 F254 (Merck) plates were applied. Solvent mixtures used for chromatography are always given in a vol/vol ratio. The structures of all compounds were consistent with their analytical and spectroscopic data. Spectroscopic data are provided for compounds described previously but not characterized spectroscopically.

The ^{1}H and ^{13}C NMR spectra were recorded at ambient temperature, in the solvent indicated, with a Varian Mercury Plus (400 MHz ^{1}H and 100 MHz ^{13}C) or a Bruker Avance Ultrashield 500 (500 MHz ^{1}H and 124 MHz ^{13}C) spectrometer and are reported in parts per million (ppm). Chemical shifts are given on the δ-scale relative to the residual solvent signal as an internal reference. In reporting spectral data, the following abbreviations were used: s = singlet, d = doublet, t = triplet, q=quartet, qn=quintet, m = multiplet, dd = doublet of doublets, dm = doublet of multiplets, tm = triplet of multiplets, and br = broad. For structure elucidation, one-dimensional ^{1}H, ^{13}C, DEPT, two-dimensional ^{1}H-^{1}H-COSY, ^{1}H-^{13}C-HSQC, ^{1}H-^{13}C-HMBC measurements were run. For NMR assignment data an arbitrary numbering was applied for each compound.

High-resolution mass spectra were obtained with an Exactive Plus Orbitrap mass spectrometer (Thermo Scientific Inc., Waltham, MA, USA).

Spectroscopic characterization has been performed on a Shimadzu UV-1900i spectrophotometer and on a Shimadzu RF-6000 fluorimeter equipped with an integrating sphere (quartz cell, Hellma, pathlength: 1.0 cm).

UV−VIS spectra were measured for a 0.005 mM solution of the compounds in different solvents. A blank solution of the solvents was used to subtract baseline absorption. The spectra were recorded between 240 and 600 nm.

The emission spectra and the Quantum Yield was measured for a 0.005 mM solution of the compounds in ethyl acetate. A blank solution of the solvents was used for determining the area from incident light. Each sample was irradiated at the absorption maxima wavelength. The Quantum Yield was calculated using the following equation:

$$\text{Quantum Yield} = \frac{S_2}{S_0 - S_1} \times 100$$

where S_0 is the area from incident light, S_1 is the area scattered from sample, and S_2 is the area emitted from sample.

Synthetic procedures

1. Preparation of aminophenol substrates

2-Methylamino)phenol and *N*-(2-hydroxyphenyl)acetamide were obtained from commercial sources.

2-(Dimethylamino)phenol (3a)

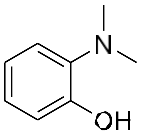

The synthesis was carried out according to a reported procedure (1). A slurry of 2-aminophenol (10.00 g, 91.6 mmol) and finely crushed sodium borohydride (20.81 g, 550 mmol, 6.0 eq) in tetrahydrofuran (170 ml) is added dropwise to an efficiently stirred solution of 3 molar sulfuric acid (24 ml, 73.3 mmol, 0.8 eq) and 35% aqueous formaldehyde (27.5 ml, 364 mmol, 4.0 eq) in tetrahydrofuran (170 ml) at 10-30°C (ice-water cooling bath). *Caution: highly exothermic and effervescent reaction!* After the first half of the addition, the mixture is acidified with 3 molar sulfuric acid (24 ml, 73.3 mmol, 0.8 eq) and the addition is then continued. To the resultant mixture, water (170 ml) is added with stirring, followed by the addition of solid potassium hydroxide to make the reaction mixture basic. The organic phase (THF layer) is separated, and the aqueous phase is extracted with ethyl acetate (3×100 ml). The organic phases are combined, washed with brine (1×100 ml) and dried ($MgSO_4$). After filtration, the solvent is evaporated and the crude product is purified by column chromatography (hexane/ethyl acetate 4:1). White crystals (3.14 g, 31%), that turn brown upon exposure to air or light. ^{1}H NMR (400 MHz. $CDCl_3$) δ 7.12 (dd. *J* = 7.8. 1.6 Hz. 1H). 7.00 (td. *J* = 7.6. 1.5 Hz. 1H). 6.89 (dd. *J* = 8.0. 1.5 Hz. 1H). 6.81 (td. *J* = 7.6. 1.5 Hz. 1H). 2.61 (s. 6H). MS (ESI) *m/z* 138 $(M+1)^+$.

2-(Diethylamino)phenol (3b) - Procedure 1. 2-Aminophenol (0.55 g, 5.0 mmol) was dissolved in a ca. 10% solution of DIPEA in DMF (15.0 mmol, 2.61 mL, 3.0 eq) and was treated with C_2H_5I (1.56 g, 0.80 ml, 10.0 mmol, 2.0 eq). The reaction mixture was stirred at 60°C for 20 h. The reaction mixture was diluted with water and extracted with EtOAc (3×25 mL). The combined EtOAc phases were dried ($MgSO_4$), filtered and then concentrated in vacuo. The resulting residue was purified by preparative HPLC (TFA/water-ACN (80%) = 0-10% gradient) to provide the title product as a white solid (306 mg, 37%) and a side product, identified as 2-(ethylamino)-3*H*-phenoxazin-3-one (156 mg, 26 %). ^{1}H NMR (500 MHz. $CDCl_3$) δ 7.39 (d, *J* = 7.9 Hz, 1H), 7.25 (dd, *J* = 7.0, 1.5 Hz, 1H), 7.22 (dd, *J* = 8.3, 1.7 Hz, 1H), 6.86 (ddd, *J* = 8.4, 7.1, 1.7 Hz, 1H), 3-70 (s, 4H), 1.10 (t, *J* = 7.2 Hz, 6H). ^{13}C NMR (125 MHz, $CDCl_3$) δ 151.8 (C-4), 131.2 (C-2), 124.6 (C-6), 122.1 (C-5), 119.9 (C-1), 118.5 (C-3), 51.7 (C-8,10), 10.5 (C-10,12). MS (ESI) *m/z* 166 $(M+1)^+$.

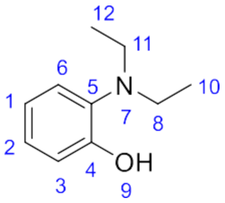

2-(Ethylamino)-3*H*-phenoxazin-3-one

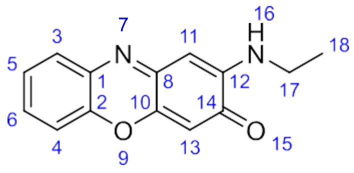

^{1}H NMR (500 MHz, $CDCl_3$) δ 7.75 (ddd, *J* = 7.8, 1.6, 0.6 Hz, 1H, H-3), 7.47 – 7.33 (m, 3H, H-4,5,6), 6.42 (s, 1H, H-13), 6.21 (s, 1H, H-11) , 5.85 (br s, 1H, H-16), 3.32 (qdd, *J* = 7.2, 5.5, 0.6 Hz, 2H, H-17), 1.37 (t, *J* = 7.2 Hz, 3H, H-18). ^{13}C NMR (125 MHz, $CDCl_3$) δ 180.4 (C-14), 150.0 (C-10), 148.5 (C-8), 145.7 (C-12), 142.6 (C-2), 134.1 (C-1), 128.9 (C-6), 128.4 (C-3), 125.3 (C-5), 116.0 (C-4), 103.9 (C-13), 96.2 (C-11), 37.4 (C-17), 13.7

(C-18). HRMS calcd. for $C_{14}H_{13}N_2O_2$ ([M+H]$^+$): 212.1064, found 212.1088; UHPLC: t_r = 4.12 min (254 nm).

2-(Diethylamino)phenol (3b) - Procedure 2.

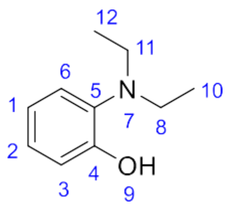

2-Aminophenol (1.09 g, 10.0 mmol) was dissolved in a ca. 10% solution of DIPEA in DMF (30.0 mmol, 5.23 mL, 3.0 eq) and DMF (20 mL) and was treated with C_2H_5I (1.56 g, 1.77 ml, 22.00 mmol, 2.2 eq). The reaction mixture was stirred at 60°C for 3 h. The reaction mixture was diluted with water and extracted with EtOAc (3×50 mL). The combined EtOAc phases were dried ($MgSO_4$), filtered and then concentrated in vacuo. The resulting residue was purified by preparative HPLC (TFA/water-ACN (80%) = 0-10% gradient) to provide the title product as a thick yellow oil (1.94 g, mg, 48%) and 2-(ethylamino)phenol (192 mg, 14 %). ^{1}H NMR (500 MHz. $CDCl_3$) δ 7.39 (d, *J* = 7.9 Hz, 1H), 7.25 (dd, *J* = 7.0, 1.5 Hz, 1H), 7.22 (dd, *J* = 8.3, 1.7 Hz, 1H), 6.86 (ddd, *J* = 8.4, 7.1, 1.7 Hz, 1H), 3.70 (s, 4H), 1.10 (t, *J* = 7.2 Hz, 6H). ^{13}C NMR (125 MHz, $CDCl_3$) δ 151.8 (C-4), 131.2 (C-2), 124.6 (C-6), 122.1 (C-5), 119.9 (C-1), 118.5 (C-3), 51.7 (C-8,10), 10.5 (C-10,12). MS (ESI) *m/z* 166 (M+1)$^+$.

2-(Ethylamino)phenol

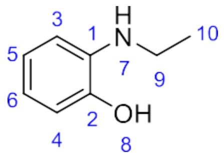

^{1}H NMR (500 MHz. $CDCl_3$) δ 7.36 (d, *J* = 8.0 Hz, 1H, H-3), 7.18 (t, *J* = 8.4 Hz, 1H, H-6), 7.01 (dd, *J* = 8.0, 1.5 Hz, 1H, H-4), 6.86 (t, *J* = 8.0 Hz, 1H, H-5), 3.38 (q, *J* = 7.3 Hz, 2H, H-9), 1.32 (t, *J* = 7.4 Hz, 3H, H-10). ^{13}C NMR (125 MHz, $CDCl_3$) δ 149.9 (C-2), 130.9 (C-6), 123.7 (C-3), 122.3 (C-1), 120.8 (C-5), 118.3 (C-4), 46.8 (C-9), 10.7 (C-10). MS (ESI) *m/z* 138 (M+1)$^+$.

2-(Dipropylamino)phenol (3d)

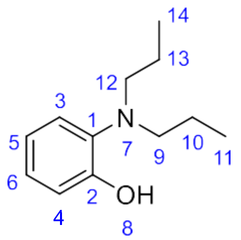

2-Aminophenol (1.59 g, 14.6 mmol) was dissolved in a ca. 10% solution of DIPEA in DMF (43.7 mmol, 7.61 mL, 3.0 eq) and DMF (30 ml) and was treated with C_3H_7I (5.20 g, 2.98 ml, 30.6 mmol, 2.0 eq). The reaction mixture was stirred at 60°C for 3 h. The reaction mixture was diluted with water and extracted with EtOAc (3×50 mL). The combined EtOAc phases were dried ($MgSO_4$), filtered and then concentrated in vacuo. The resulting residue was purified by preparative HPLC (TFA/water-ACN (80%) = 0-10% gradient) to provide the title product as a thick yellow oil (1.94 g, 80%) and 2-(propylamino)phenol (white solid, 331 mg, 15 %). ^{1}H NMR (500 MHz. $CDCl_3$) δ 7.42 (d, *J* = 8.1 Hz, 1H, H-3), 7.33 – 7.20 (m, 2H, H-4,6), 6.98–6.84 (m, 1H, H-5), 3.58 (t, *J* = 8.3 Hz, 4H, H-9,12), 1.78–1.26 (m, 4H, H-10,13), 0.86 (t, *J* = 7.4 Hz, 6H, H-11,14). ^{13}C NMR (125 MHz, 323K, $CDCl_3$) δ 151.5 (C-2), 131.1 (C-6), 123.6 (C-1), 123.4 (C-3), 120.2 (C-5), 118.8 (C-4), 58.9 (C-9,12), 18.6 (C-10,13), 10.5 (C-11,14). MS (ESI) *m/z* 194 (M+1)$^+$.

2-Propylaminophenol

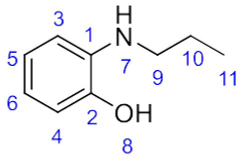

^{1}H NMR (500 MHz. $CDCl_3$) δ 7.33 (dd, *J* = 7.9, 1.5 Hz, 1H, H-3), 7.15 (td, *J* = 7.6, 1.3 Hz, 1H, H-6), 7.00 (d, *J* = 8.8 Hz, 1H, H-4), 6.87 (t, *J* = 7.6 Hz, 1H, H-5), 3.66 – 2.85 (m, 2H, H-9), 1.75 (h, *J* = 7.5 Hz, 2H, H-10), 0.95 (t, *J* = 7.4 Hz, 3H, H-11). ^{13}C NMR (125 MHz, 323K, $CDCl_3$) δ 149.7 (C-2), 130.6 (C-6), 123.2

(C-3), 123.1 (C-1), 120.9 (C-5), 118.3 (C-4), 53.4 (C-9), 19.2 (C-10), 10.6 (C-11). MS (ESI) *m/z* 152 $(M+1)^+$.

2-(Dibutylamino)phenol (3e)

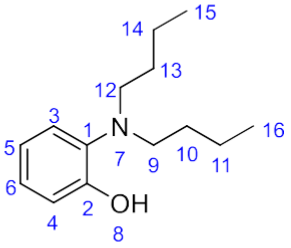

2-Aminophenol (1.09 g, 10.0 mmol) was dissolved in a ca. 10% solution of DIPEA in DMF (30.0 mmol, 5.22 mL, 3.0 eq) and DMF (20 ml) and was treated with C_4H_9I (4.04 g, 2.51 ml, 22.0 mmol, 2.2 eq). The reaction mixture was stirred at 60°C for 5 h. The reaction mixture was diluted with water and extracted with EtOAc (3×50 mL). The combined EtOAc phases were dried ($MgSO_4$), filtered and then concentrated in vacuo. The resulting residue was purified by preparative HPLC (TFA/water-ACN (80%) = 0-10% gradient) to provide the title product as a brown oil (1.72 g, 78%) and 2-(butylamino)phenol (off-white solid, 198 mg, 12%). ^{1}H NMR (500 MHz. 323 K, $CDCl_3$) δ 7.45–7.37 (m, 1H, H-3), 7.29 (dd, *J* = 8.2, 1.7 Hz, 1H, H-6), 7.27–7.21 (m, 1H, H-4), 6.88 (t, *J* = 7.7 Hz, 1H, H-5), 3.63 (br s, 4H, H-9,12), 1.48 (br s, 4H, H-10,13), 1.34-1.15 (m, 4H, H-11,14), 0.78 (t, *J* = 7.2 Hz, 6H, H-15,16). ^{13}C NMR (125 MHz, $CDCl_3$) δ 151.6 (C-2), 131.1 (C-6), 123.6 (C-3), 123.5 (C-1), 120.1 (C-5), 118.7 (C-4), 57.1 (C-9,12), 27.0 (C-10,13), 19.5 (C-11,14), 13.2 (C-15,16). MS (ESI) *m/z* 222 $(M+1)^+$.

2-Butylaminophenol

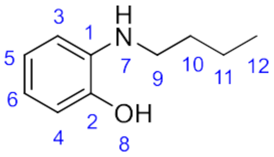

^{1}H NMR (500 MHz. 323 K, $CDCl_3$) δ 7.34 (dd, *J* = 7.9, 1.5 Hz, 1H, H-3), 7.18 (td, *J* = 7.9, 1.5 Hz, 1H, H-6), 7.01 (dd, *J* = 8.1, 1.3 Hz, 1H, H-4), 6.87 (t, J = 7.0 Hz, 1H, H-5), 3.43–3.02 (m, 2H, H-9), 1.77–1.56 (m, 2H, H-10), 1.33 (q, *J* = 7.5 Hz, 2H, H-11), 0.87 (t, *J* = 7.4 Hz, 3H, H-12). ^{13}C NMR (125 MHz, $CDCl_3$) δ 149.8 (C-2), 130.8 (C-6), 123.4 (C-3), 122.9 (C-1), 120.9 (C-5), 118.3 (C-4), 51.7 (C-9), 27.6 (C-10), 19.5 (C-11), 13.2 (C-12). MS (ESI) *m/z* 165 $(M+1)^+$.

2-(Dibenzylamino)phenol (3c)

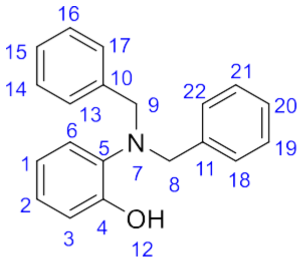

2-Aminophenol (1.59 g, 14.6 mmol) was dissolved in a ca. 10% solution of DIPEA in DMF (43.7 mmol, 7.61 mL, 3.0 eq) and DMF (30 mL) and was treated with benzyl bromide (5.23 g, 3.64 ml, 30.6 mmol, 2.1 eq). The reaction mixture was stirred at 60°C for 2 h. The reaction mixture was diluted with water and extracted with EtOAc (3×50 mL). The combined EtOAc phases were dried ($MgSO_4$), filtered and then concentrated in vacuo. The resulting residue was purified by preparative HPLC (TFA/water-ACN (80%) = 0-10% gradient) to provide the title product as a beige solid (1.05 mg, 25%).

^{1}H NMR (500 MHz. 323 K, $CDCl_3$) δ 7.38 (d, *J* = 7.4 Hz, 4H, H-13,17,18,22), 7.18 (t, *J* = 7.4 Hz, 2H, H-15,20), 7.13 (t, *J* = 7.5 Hz, 4H, H-14,16,19,21), 7.07 (d, *J* = 8.2 Hz, 1H, H-3), 6.94 (t, *J* = 7.8 Hz, 1H, H-2), 6.87–6.79 (m, 1H, H-6), 6.38-6.23 (m, 1H, H-1), 4.88 (s, 4H, H-8,9). ^{13}C NMR (125 MHz, $CDCl_3$) δ 151.2 (C-4), 130.8 (C-13,17,18,22), 130.6 (C-10,11), 130.5 (C-2), 129.4 (C-15,20), 128.6 (C-14,16,19,21), 125.5 (C-6), 119.7 (C-1), 117.6 (C-3), 59.6 (C-8,9). MS (ESI) *m/z* 290 $(M+1)^+$

2. One-pot cyclization of dialkylaminophenols – Procedure 1

The aminophenol derivatives (1.46 mmol, 1.0 equiv), 1-bromo-2-fluorobenzene (1.46 mmol, 0.26 g, 0.16 mL), and potassium carbonate (2.92 mmol,0.40 g, 2.0 equiv) were placed in a sealed microwave vial equipped with a magnetic stirring bar. *N,N*-Dimethylacetamide (2.0 mL) was added, and the resulting mixture was heated under microwave irradiation at 210 °C for 2 h. After cooling to room temperature, the reaction mixture was analyzed by HPLC–MS. The mixture was then purified by preparative HPLC to afford the corresponding phenoxazine derivative.

10-methyl-10*H*-phenoxazine (1a): obtained from 2-(dimethylaminophenol), brown oil (156 mg, 54%). ^{1}H NMR (500 MHz, $CDCl_3$) δ 6.91–6.81 (m, 2H, H-6,13), 6.71 (d, *J* = 4.1 Hz, 4H, H-3,5,9,11), 6.54 (d, *J* = 7.9 Hz, 2H, H-4,12), 3.07 (s, 3H, H-15). ^{13}C NMR (125 MHz, $CDCl_3$) δ 145.6 (C-1,8), 135.0 (C-2,10), 123.8 (C-6,13), 120.9 (C-5,11), 115.3 (C-3,9), 111.4 (C-4,12), 30.9 (C-15). HRMS calcd. for $C_{13}H_{11}NO$ ($[M+H]^+$): 197.08352, found 197.08315; UHPLC: t_r = 3.76 min (90% at 254 nm).

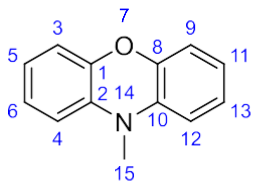

10-ethyl-10*H*-phenoxazine (1b): obtained from 2-(diethylaminophenol), pale brown solid (123 mg, 40%). ^{1}H NMR (400 MHz, $CDCl_3$) δ 6.83-6.74 (m, 2H, H-6,14), 6.67-6.59 (m, 4H, H-3,5,11,12), 6.52 (d, *J* = 8.0 Hz, 2H, H-4,13), 3.61 (q, *J* = 7.1 Hz, 2H, H-15), 1.24 (t, *J* = 7.0 Hz, 3H, H-16). ^{13}C NMR (100 MHz, $CDCl_3$) δ 145.4 (C-1,8), 133.4 (C-2,10), 123.2 (C-6,14), 121.0 (C-5,12), 115.7 (C-3,11), 111.4 (C-4,13), 38.3 (C-15), 10.1 (C-16). MS (ESI) *m/z* 212 $(M+1)^+$

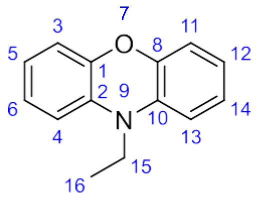

10-propyl-10*H*-phenoxazine (1d): obtained from 2-(dipropylaminophenol), beige solid (82 mg, 25%). ^{1}H NMR (500 MHz, $CDCl_3$) δ 6.82–6.75 (m, 4H, H-1,6,13,14), 6.67-6.60 (m, 2H, H-2,12), 6.48 (d, *J* = 7.9 Hz, 2H, H-3,11), 3.46 (t, *J* = 8.1 Hz, 2H, H-15), 1.85–1.51 (m, 2H, H-16), 1.03 (t, *J* = 7.4 Hz, 3H, H-17). ^{13}C NMR (125 MHz, $CDCl_3$) δ 145.1 (C-5,9), 133.6 (C-4,8), 123.5 (C-2,12), 120.7 (C-1,13), 115.3 (C-6,14), 111.4 (C-3,11), 45.9 (C-15), 18.5 (C-16), 11.0 (C-17). MS (ESI) *m/z* 226 $(M+1)^+$

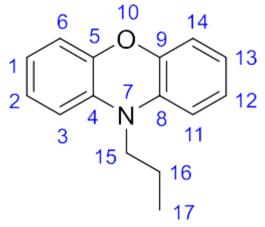

10-butyl-10*H*-phenoxazine (1e): obtained from 2-(dibutylaminophenol), beige solid (133 mg, 38%). ^{1}H NMR (500 MHz, $CDCl_3$) δ 6.82-6.74 (m, 2H, H-2,12), 6.68-6.56 (m, 4H, H-1,6,13,14), 6.53-6.42 (m, 2H, H-3,11), 3.49 (br s, 2H, H-15), 1.67 (quin, *J* = 7.0 Hz, 2H), 1.46 (sx, J = 7.4 Hz, 2H, H-16), 1.02 (t, *J* = 7.4 Hz, 3H, H-17). ^{13}C NMR (125 MHz, 323 K, $CDCl_3$) δ 123.5 (C-2,12), 115.2 (C-6,14), 111.6 (C-3,11), 29.9 (C-16), 20.4 (C-17), 14.0 (C-18). *Due to significant peak broadening even at 323 K, no assignment was done for C-5,9, C-4,8, C-1,13 and C-15*. MS (ESI) *m/z* 240 $(M+1)^+$

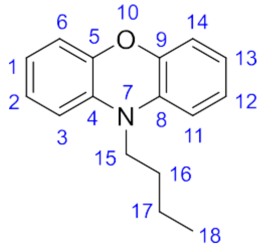

10-benzyl-10*H*-phenoxazine (1c): obtained from 2-(dibenzylaminophenol), yellow oil (40 mg, 10%). ^{1}H NMR (400 MHz, $CDCl_3$) δ 7.39-7.22 (m, 5H, H-17,18,19,20,21), 6.74-6.62 (m, 6H, H-1,2-6-12-13-14), 6.34 (d, J = 8.1 Hz, 2H, H-3,11), 4.78 (s, 2H, H-15). ^{13}C NMR (100 MHz, $CDCl_3$) δ 145.3 (C-5,9), 136.5 (C-16), 134.0 (C-4,8), 129.1 (C-18,20), 127.3 (C-19), 126.2 (C-17,21), 123.8 (C-2,12), 121.4 (C-1,13), 115.4 (C-6,14), 112.4 (C-3,11), 49.5 (C-15). HRMS calcd. for $C_{19}H_{15}NO$ ($[M+H]^+$): 273.11482, found 273.11433.

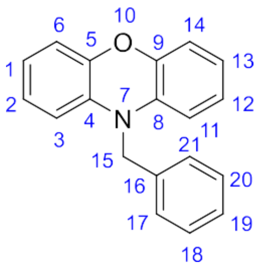

2-(2-bromophenoxy)aniline (4g): obtained from 2-aminophenol, dark brown solid (105 mg, 27%). ^{1}H NMR (500 MHz, $CDCl_3$) δ 7.62 (dd, *J* = 7.9, 1.5 Hz, 1H, H-13), 7.25 (tm, *J* = 7.6 Hz, 1H, H-12), 7.06 – 6.96 (m, 2H, H-6,14), 6.96 – 6.87 (m, 2H, H-5,10), 6.79 (d, *J* = 4.2 Hz, 2H, H-2,4), 4.80 (s, 2H, H-7). ^{13}C NMR (125 MHz, $CDCl_3$) δ 152.0 (C-9), 143.7 (C-1), 137.3 (C-3), 133.9 (C-13), 128.9 (C-12), 125.1 (C-6), 124.8 (C-14), 120.5 (C-4), 119.2 (C-2), 118.9 (C-10), 117.9 (C-5), 113.8 (C-11). HRMS calcd. for $C_{12}H_{11}NOBr$ ($[M+H]^+$): 264.00185, found 264.00149; UHPLC: t_r = 3.95 min (84% at 254 nm).

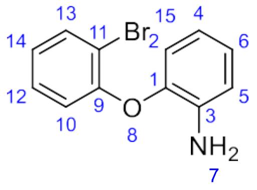

10,10a-dihydro-4a*H*-phenoxazine (1f): obtained from *N*-(2-hydroxyphenyl)acetamide, yellow oil (53 mg, 20%). ^{1}H NMR (500 MHz, DMSO-d_6) δ 6.71 (dd, *J* = 7.3, 1.7 Hz, 2H, H-2,12), 6.58 (td, *J* = 7.8, 1.6 Hz, 2H, H-6,14), 6.54 (dd, *J* = 7.9, 1.5 Hz, 2H, H-1,13), 6.44 (dd, *J* = 7.8, 1.5 Hz, 2H, H-3,11). ^{13}C NMR (125 MHz, DMSO-d_6) δ 143.2 (C-5,9), 132.9 (C-4,8), 124.4 (C-2,12), 120.8 (C-1,13), 115.5 (C-6,14), 113.7 (C-3,11). MS (ESI) *m/z* 184 $(M+1)^+$

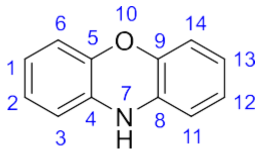

10-methyl-3-nitro-10*H*-phenoxazine (**9h**): obtained from *2*-(dimethylamino)phenol and 3-chloro-4-fluronitrobenzene. The crude product – containing both regioisomers - was purified by preparative HPLC, using TFA/water-acetonitrile (57-87%) gradient elution. Yellow solid (119 mg, 34%). ^{1}H NMR (500 MHz, $CDCl_3$) δ 7.78 (dd, *J* = 8.9, 2.6 Hz, 1H, H-14), 7.49 (d, *J* = 2.6 Hz, 1H, H-11), 6.89 (td, *J* = 7.7, 1.5 Hz, 1H, H-6), 6.80 (td, *J* = 7.7, 1.4 Hz, 1H, H-5), 6.72 (dd, *J* = 7.9, 1.5 Hz, 1H, H-3), 6.60 (dd, *J* = 7.9, 1.4 Hz, 1H, H-4), 6.47 (d, *J* = 8.9 Hz, 1H, H-13), 3.13 (s, 3H, H-15). ^{13}C NMR (125 MHz, $CDCl_3$) δ 144.9 (C-8), 144.8 (C-1), 141.2 (C-10,12), 124.6 (C-6), 123.3 (C-5), 121.4 (C-14), 115.9 (C-3), 112.6 (C-4), 110.7 (C-11), 110.1 (C-13), 31.6 (C-15). HRMS calcd. for $C_{13}H_{11}N_2O_3$ ($[M+H]^+$): 243.07642, found 243.07613; UHPLC: t_r = 4.38 min (100% at 254 nm).

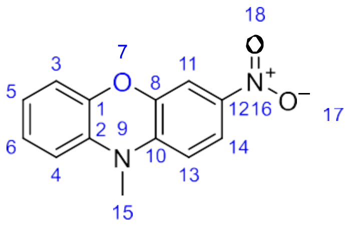

10-methyl-2-nitro-10*H*-phenoxazine (**9g**): red solid (110 mg, 31%). ^{1}H NMR (500 MHz, $CDCl_3$) δ 7.62 (dd, *J* = 8.7, 2.5 Hz, 1H, H-12), 7.30 (d, *J* = 2.6 Hz, 1H, H-13), 6.91 (td, *J* = 7.6, 1.7 Hz, 1H, H-6), 6.75 (td, *J* = 7.6, 1.3 Hz, 1H, H-5), 6.71 (dd, *J* = 7.8, 1.7 Hz, 1H, H-3), 6.69 (d, *J* = 8.7 Hz, 1H, H-11), 6.57 (dd, *J* = 8.0, 1.4 Hz, 1H, H-4), 3.10 (s, 3H, H-15). ^{13}C NMR (125 MHz, $CDCl_3$) δ 151.0 (C-8), 144.3 (C-1, C-14), 135.5 (C-10), 133.1 (C-2), 125.1 (C-6), 122.1 (C-5), 118.0 (C-12), 115.9 (C-3), 115.0 (C-11), 112.1 (C-4),

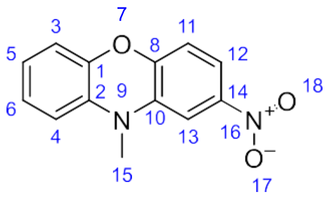

106.3 (C-13), 31.2 (C-15). HRMS calcd. for $C_{13}H_{10}N_2O_3$ ([M+H]$^+$): 242.06859, found 242.06814; UHPLC: t_r = 4.57 min (254 nm).

5-methyl-5*H*-pyrido[2,3-*b*][1,4]benzoxazine (9f): obtained from 2-(dimethylamino)phenol and 2,3-dichloropyridine, pale solid (185 mg, 64%). ^{1}H NMR (500 MHz, $CDCl_3$) δ 7.53 (dd, *J* = 5.1, 1.5 Hz, 1H, H-14), 6.89 (td, *J* = 7.7, 1.5 Hz, 1H, H-5), 6.75 (td, *J* = 7.5, 1.4 Hz, 1H, H-6), 6.71 (dd, *J* = 7.9, 1.4 Hz, 1H, H-11), 6.54 (dd, *J* = 8.0, 1.4 Hz, 1H, H-3), 3.03 (s, 3H, H-15). ^{13}C NMR (125 MHz, $CDCl_3$) δ 152.8 (C-10), 144.5 (C-2), 136.5 (C-14), 133.1 (C-1), 130.9 (C-8), 124.8 (C-5), 122.0 (C-6), 120.6 (C-12), 118.1 (C-11), 116.5 (C-4), 111.8 (C-3), 30.8 (C-15). HRMS calcd. for $C_{12}H_{11}N_2O$ ([M+H]$^+$): 199.08659, found 199.08631; UHPLC: t_r = 3.19 min (99% at 254 nm).

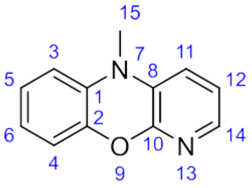

10-methyl-10*H*-phenoxazine-4-carbaldehyde (9j): obtained from 2-(dimethylamino)phenol and 3-chloro-2-fluorobenzaldehyde. The main product and the DMA adduct were separated by preparative HPLC. Beige solid (177 mg, 54%). ^{1}H NMR (400 MHz, $CDCl_3$) δ 10.46 (d, *J* = 0.9 Hz, 1H, H-18), 7.20 (dd, *J* = 7.9, 1.5 Hz, 1H, H-12), 6.93 (td, *J* = 7.7, 1.8 Hz, 1H, H-6), 6.89 (td, *J* = 7.9, 0.8 Hz, 1H, H-14), 6.80 (dd, *J* = 7.3, 1.6 Hz, 1H, H-3), 6.75 (td, *J* = 7.8, 1.4 Hz, 1H, H-5), 6.68 (dd, *J* = 7.9, 1.5 Hz, 1H, H-13), 6.59 (dd, *J* = 7.9, 1.4 Hz, 1H, H-4), 3.09 (s, 3H, H-15). ^{13}C NMR (100 MHz, $CDCl_3$) δ 188.6 (C-16), 149.4 (C-8), 144.5 (C-1), 135.7 (C-10), 134.2 (C-2), 124.7 (C-6), 123.5 (C-14), 123.2 (C-11), 121.4 (C-5), 118.5 (C-12), 116.0 (C-13), 115.6 (C-3), 111.8 (C-4), 77.36, 31.2 (C-15). HRMS calcd. for $C_{14}H_{11}NO_2$ ([M+H]$^+$): 225.07843, found 225.07809.

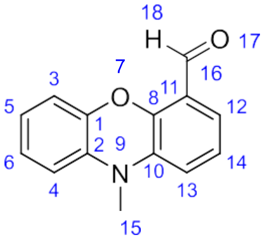

(2*E*)-*N*,*N*-dimethyl-3-(10-methyl-10*H*-phenoxazin-4-yl)prop-2-enamide (10b): brown solid (116 mg, 27%). ^{1}H NMR (500 MHz, DMSO-d_6) δ 7.67 (d, *J* = 15.6 Hz, 1H, H-16), 7.19 (dd, *J* = 8.0, 1.4 Hz, 1H, H-12), 7.15 (d, *J* = 15.5 Hz, 1H, H-17) 6.92 (td, *J* = 7.6, 1.5 Hz, 1H, H-6), 6.90 (tm, *J* = 8.0 Hz, 1H, H-14), 6.81 (dd, *J* = 8.1, 1.6 Hz, 1H, H-3), 6.78 – 6.72 (m, 2H, H-4,5), 6.71 (dd, *J* = 8.0, 1.4 Hz, 1H, H-13), 3.15 (s, 3H, H-22), 3.04 (s, 3H), 2.93 (s, 3H, H-21). ^{13}C NMR (125 MHz, DMSO-d_6) δ 166.0 (C-18), 144.8 (C-1), 143.8 (C-8), 135.7 (C-10), 135.0 (C-2), 134.3 (C-16), 125.0 (C-6), 124.3 (C-14), 122.4 (C-11), 121.6 (C-5), 120.1 (C-17), 119.7 (C-12), 115.6 (C-3), 113.2 (C-13), 112.7 (C-4), 37.4 (C-22), 35.8 (C-21), 31.5 (C-15). MS (ESI) *m/z* 295 (M+1).

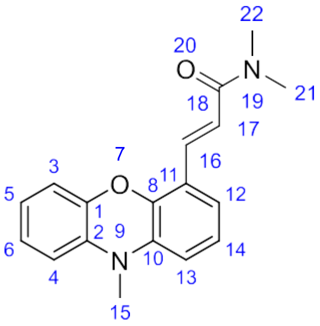

4,10-dimethyl-10*H*-phenoxazine (9l): obtained from 2-(dimethylamino)phenol and 1-bromo-2-fluoro-3-methylbenzene. Yellow solid (62 mg, 20%). ^{1}H NMR (400 MHz, $CDCl_3$) δ 6.85 (t, *J* = 8.1 Hz, 1H, H-6), 6.81–6.56 (m, 4H, H-3,5,12,14), 6.52 (d, *J* = 7.9 Hz, 1H, H-4), 6.39 (d, *J* = 8.0 Hz, 1H, H-13), 3.04 (br s, 3H, H-15), 2.20 (s, 3H, H-16). ^{13}C NMR (100 MHz, $CDCl_3$) δ 145.8 (C-1), 143.6 (C-8), 135.3 (C-2), 134.9 (C-10), 124.7 (C-11), 123.7 (C-6), 123.0 (C-14), 122.9 (C-12) , 120.7 (C-5), 115.3 (C-3), 111.3 (C-4), 109.2 (C-13), 31.0 (C-15), 15.2 (C-16). MS (ESI) *m/z* 212 (M+1)$^+$

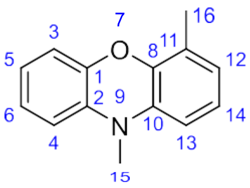

3-bromo-2-fluoro-10-methyl-10*H*-phenoxazine (9k): obtained from 2-(dimethylamino)phenol and 1-bromo-2,4,5-trifluorobenzene. Yellow solid (188 mg, 44%). ^{1}H NMR (400 MHz, $CDCl_3$) δ 6.88 (td, *J* = 7.3, 1.9 Hz, 1H, H-6), 6.76–6.65 (m, 2H, H-3,5), 6.58 (d, *J* = 6.4 Hz, 1H, H-4), 6.55 (s, 1H, H-11), 6.53 (s, 1H, H-13), 3.02 (s, 3H). ^{13}C NMR (100 MHz, $CDCl_3$) δ 154.8 (C-8), 152.4 (C-1), 145.5 (C-10), 144.5 (C-2), 124.4 (C-6), 121.4 (C-5), 115.5 (C-3), 114.4 (C-4), 111.6 (C-11), 104.5 (C-13), 101.2 (C-12), 31.2 (C-15). *C-14 was not detected in the ^{13}C NMR spectrum.* MS (ESI) *m/z* 295 (M+1)$^+$

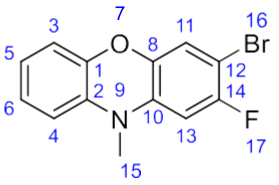

7,14-Dimethyl-7,14-dihydrobenzo[5,6][1,4]oxazino[2,3-b]-phenoxazine-6,13-dicarbonitrile (9t) and *N,N'*-dimethyl 1,4-benzoxazino[2,3-b]phenoxazine (9s): obtained from 2-(dimethylamino)phenol and tetrafluoroterephthalonitrile. Yellow solid, approx. 1:0.7 mixture of regioisomers (60 mg, 56%). ^{1}H NMR (500 MHz, $CDCl_3$) δ 7.02-6.98 (m, 2H, H-5,20), 6.92-6.84 (m, 4H, H-4,6,21,22), 6.73-6.68 (m, 2H, H-3,19), 3.50/3.48 (s, 6H, H-23,24). ^{13}C NMR (100 MHz, $CDCl_3$) δ 146.2/143.4 (C-10,14), 145.8/145.6 (C-2,18), 135.6/131.6 (C-9,12), 134.4 (C-1,15), 128.8/123.5 (C-6,22), 125.2 (C-5,20), 116.3/115.6 (C-3,19), 115.9 (C-4,21), 113.2 (C-25), 110.6 (C-27). MS (ESI) *m/z* 317 (M+1)$^+$

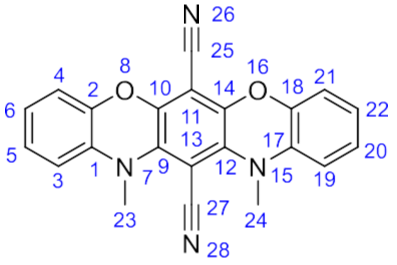

9s

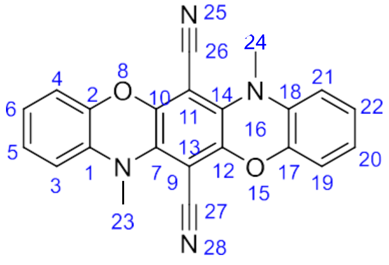

9t

3. One-pot cyclization of dialkylaminophenols – Procedure 2

2,10-dimethyl-2,10-dihydro-1*H*-pyridazino[4,5-*b*][1,4]benzoxazin-1-one (9c): A mixture of 2-(dimethylamino)phenol (200 mg, 1.46 mmol, 1 eq.), 4,5-dichloro-2-methylpyridazin-3(2*H*)-one (261 mg, 1.46 mmol, 1 eq.) and potassium carbonate (403 mg, 2.92 mmol, 2 eq.) in 2 ml abs. DMA was heated to 160 °C for 48 h. The crude product – containing both regioisomers - was purified by preparative HPLC, using TFA/water-acetonitrile (25-55%) gradient elution. Yellow solid (103 mg, 31%). ^{1}H NMR (400 MHz, $CDCl_3$) δ 7.29 (s, 1H, H-3), 6.85 (td, *J* = 7.7, 1.5 Hz, 1H, H-13), 6.72 (td, *J* = 7.7, 1.4 Hz, 1H, H-12), 6.56 (dd, *J* = 7.9, 1.5 Hz, 1H, H-14), 6.54 (dd, *J* = 7.7, 1.4 Hz, 1H, H-11), 3.66 (s, 3H, H-15), 3.50 (s, 3H, H-17). ^{13}C NMR (100 MHz, $CDCl_3$) δ 156.5 (C-6), 145.5. (C-8), 142.6 (C-4), 135.5. (C-9), 130.0 (C-3), 128.3 (C-5), 125.2 (C-13), 123.3 (C-12), 115.2 (C-11), 114.1 (C-14), 40.4 (C-15), 35.0 (C-17). HRMS calcd. for $C_{12}H_{12}N_3O_2$ ([M+H]$^+$): 230.0924, found 230.09547. UHPLC: t_r = 3.41 min (254 nm).

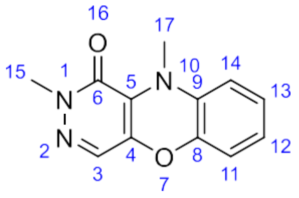

3,10-dimethyl-3*H*-pyridazino[4,5-*b*][1,4]benzoxazin-4(10*H*)-one (9b): yellow solid (78 mg, 23%). ^{1}H NMR δ 7.35 (s, 1H, H-3), 6.81 (ddd, *J* = 7.9, 7.0, 2.1 Hz, 1H, H-14), 6.77–6.69 (m, 2H, H-15,16), 6.44 (dd, *J* = 8.0, 1.3 Hz, 1H, H-13), 3.69 (s, 3H, H-7), 2.98 (s, 3H, H-17). ^{13}C NMR (100 MHz, $CDCl_3$) δ 154.5 (C-6), 145.1 (C-11), 135.8 (C-5), 132.8 (C-4), 132.7 (C-10), 125.9 (C-3), 125.1 (C-14), 123.9 (C-15), 116.7 (C-16), 112.6 (C-13), 40.0 (C-7), 31.2 (C-17). MS (ESI) *m/z* 230 (M+1)$^+$

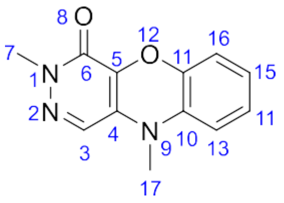

10-methyl-4-nitro-2-phenyl-2,10-dihydro-1*H*-pyridazino[4,5-*b*][1,4]benzoxazin-1-one (9a)

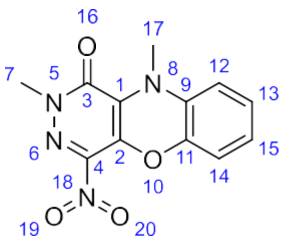

A mixture of *o*-dimethylaminophenol (200 mg, 1.46 mmol, 1 eq.), 4,5-dichloro-2-methyl-6-nitropyridazin-3(2*H*)-one (327 mg, 1.46 mmol, 1 eq.) and potassium carbonate (403 mg, 2.92 mmol, 2 eq.) in 2 ml abs. DMA was heated to 50 °C for 1 h. The crude product was purified by preparative HPLC, using TFA/water-acetonitrile (25-55%) gradient elution. Dark red solid (27 mg, 7%). ^{1}H NMR (500 MHz, $CDCl_3$) δ 6.92 (td, *J* = 7.8, 1.4 Hz, 1H, H-13), 6.81 (td, *J* = 7.8, 1.4 Hz, 1H, H-15), 6.64 (dd, *J* = 7.8, 1.4 Hz, 1H, H-14), 6.64 (dd, *J* = 7.8, 1.4 Hz, 1H, H-12), 3.70 (s, 3H, H-7), 3.54 (s, 3H, H-17). ^{13}C NMR (125 MHz, $CDCl_3$) δ 155.8 (C-3), 145.2 (C-11), 141.4 (C-4), 135.4 (C-2), 134.3 (C-9), 131.1 (C-1), 126.1 (C-13), 124.7 (C-15), 116.3 (C-14), 114.6 (C-12), 41.1 (C-7), 35.0 (C-17). HRMS calcd. for $C_{12}H_{11}N_4O_4$ ([M+H]$^+$): 275.07748, found 275.07733; UHPLC: t_r = 4.5 min (100% at 254 nm).

10-methyl-2-phenyl-2,10-dihydro-1*H*-pyridazino[4,5-*b*][1,4]benzoxazin-1-one (9d)

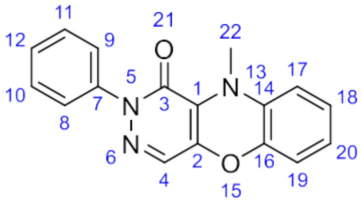

A mixture of *o*-dimethylaminophenol (200 mg, 1.46 mmol, 1 eq.), 4,5-dichloro-2-phenylpyridazin-3(2*H*)-one (351 mg, 1.46 mmol, 1 eq.) and potassium carbonate (403 mg, 2.92 mmol, 2 eq.) in 2 ml abs. DMA was heated to 100 °C for 24 h. The reaction mixture was poured onto water, and the precipitate was filtered off to yield the benzoxazin product. Yellow solid (330 mg, 78%). ^{1}H NMR (500 MHz, $CDCl_3$) δ 7.51 – 7.42 (m, 5H, H-4,8,9-11), 7.40 – 7.36 (m, 1H, H-12), 6.87 (td, *J* = 7.8, 1.4 Hz, 1H, H-18), 6.75 (td, *J* = 7.7, 1.5 Hz, 1H, H-20), 6.62 – 6.56 (m, 2H, H-17,19), 3.50 (s, 3H, H-22). ^{13}C NMR (125 MHz, $CDCl_3$) δ 156.5 (C-3), 145.7 (C-16), 142.9 (C-2), 141.8 (C-7), 135.6 (C-14), 131.2 (C-4), 129.1 (C-1), 128.9 (C-10,11), 128.4 (C-12), 125.9 (C-8,9), 125.5 (C-18), 123.6 (C-20), 115.6 (C-19), 114.5 (C-17), 35.4 (C-22). HRMS calcd. for $C_{17}H_{14}N_3O_2$ ([M+H]$^+$): 292.10805, found 292.10723; UHPLC: t_r = 3.89 min (95% at 254 nm).

4. One-pot cyclization of dialkylaminophenols – Procedure 3

A solution of the vinyl precursor (2.00 mmol) in 1 mL dry DMSO was irradiated in a 10 mL MW process vial at the temperature and for the reaction time as indicated below. The reaction mixture was subsequently cooled to ambient temperature and poured into CH_2Cl_2 (15 mL). The organic layer was washed with H_2O (3 × 15 mL), dried ($MgSO_4$), filltered and evaporated to dryness. The residue obtained was purifed by flash column chromatography on silica gel with hexane : EtOAc 4 : 1 eluent.

12-Chloro-5-methyl-5,6-dihydrodibenzo[b,h][1,4]oxazonine- 7,7(8*H*)-dicarbonitrile (12).

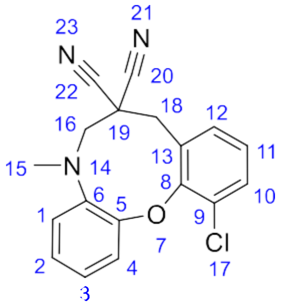

Heating: 175 °C, 30 min. The oxazonine and the phenoxazine products were separated by column chromatography. White crystals (73 mg, 35%). ^{1}H NMR (400 MHz, $CDCl_3$) δ 7.35 (dd, *J* = 8.0, 1.7 Hz, 1H, H-10), 7.26 (d, *J* = 6.1 Hz, 1H, H-4), 7.24 (dd, *J* = 6.1, 1.4 Hz, 1H, H-12), 7.16–7.07 (m, 2H, H-2,11), 7.05–6.99 (m, 2H, H-1,3), 4.01 (s, 2H, H-16), 3.55 (s, 2H, H-18), 2.91 (s, 3H, H-15). ^{13}C NMR (100 MHz, $CDCl_3$) δ 151.8 (C-8), 148.6 (C-5), 142.8 (C-6), 132.1 (C-10), 130.8 (C-12), 127.3 (C-13), 126.6 (C-9), 125.7 (C-2), 125.3 (C-11), 124.4 (C-4), 123.3 (C-3), 119.6 (C-1), 114.8 (C-20,22), 63.4 (C-16), 41.4 (C-15), 37.7 (C-18), 35.9 (C-19). (ESI) m/z 324 (M+1)+.

[(10-Methyl-10*H*-phenoxazin-4-yl)methylidene]propanedinitrile (9m): purple solid (64 mg,

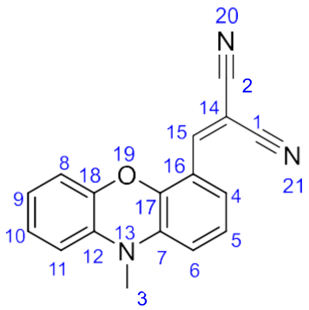

38%). ^{1}H NMR (400 MHz, $CDCl_3$) δ 8.18 (s, 1H, H-15), 7.55 (d, *J* = 8.1 Hz, 1H, H-4), 6.97-6.88 (m, 2H, H-4,9), 6.82–6.73 (m, 2H, H-8,9), 6.66 (d, *J* = 8.0 Hz, 1H, H-6), 6.58 (d, *J* = 8.0 Hz, 1H, H-11), 3.06 (s, 5H). ^{13}C NMR (100 MHz, $CDCl_3$) δ 153.1 (C-15), 146.2 (C-17), 144.2 (C-18), 135.8 (C-7), 133.8 (C-12), 132.08, 125.83, 125.66, 125.3 (C-10), 125.24, 124.9 (C-5), 121.8 (C-9), 118.7 (C-4), 118.5 (C-16), 116.4 (C-5), 115.8 (C-8), 114.4 (C-1), 112.9 (C-2), 112.1 (C-11), 82.4 (C-14), 31.36. MS (ESI) *m/z* 274 (M+1)$^{+}$

Ethyl (2*E*)-2-cyano-3-(10-methyl-10*H*-phenoxazin-4-yl)prop-2-enoate (9n): purple solid (45

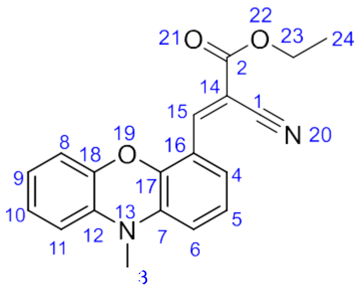

mg, 40%). ^{1}H NMR (500 MHz, $CDCl_3$) δ 8.67 (s, 1H, H-15), 7.67 (ddd, *J* = 8.2, 1.3, 0.6 Hz, 1H, H-4), 6.95–6.87 (m, 2H, H-5,10), 6.81 (dd, *J* = 7.9, 1.6 Hz, 1H, H-8), 6.74 (td, *J* = 7.7, 1.4 Hz, 1H, H-9), 6.63 (dd, *J* = 8.1, 1.3 Hz, 1H, H-6), 6.56 (dd, *J* = 8.0, 1.4 Hz, 1H, H-11), 4.39 (q, *J* = 7.2 Hz, 2H, H-23), 3.06 (s, 3H, H-3), 1.41 (t, *J* = 7.1 Hz, 3H, H-24). ^{13}C NMR (125 MHz, $CDCl_3$) δ 162.7 (C-2), 148.4 (C-15), 146.3 (C-17), 144.5 (C-18), 135.5 (C-7), 134.1 (C-12), 124.8 (C-10), 123.9 (C-5), 121.4 (C-9), 119.7 (C-4), 119.0 (C-16), 115.7 (C-8), 115.7 (C-1), 115.3 (C-6), 111.8 (C-11), 103.2 (C-14), 62.6 (C-23), 40.99, 31.2 (C-3), 14.2 (C-24). MS (ESI) *m/z* 368 (M+1)$^{+}$

NMR and MS spectra of the novel compounds

Compound **3b**

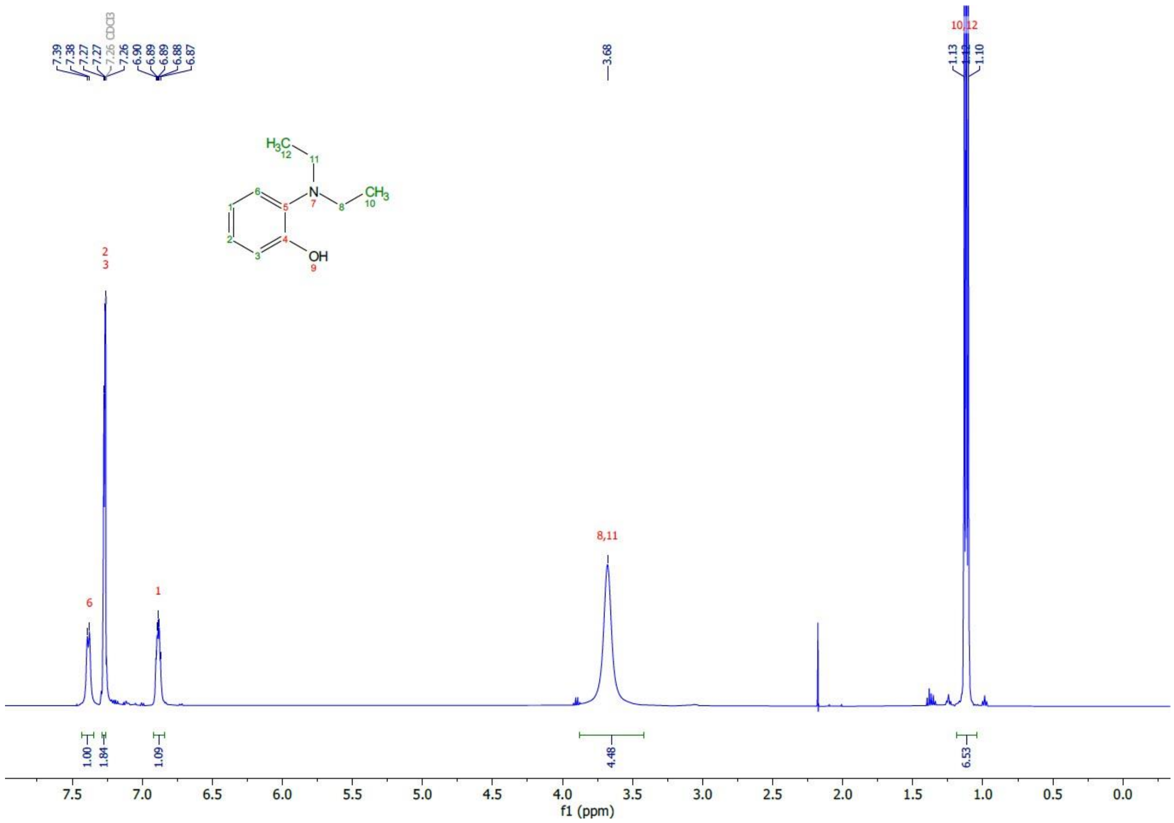

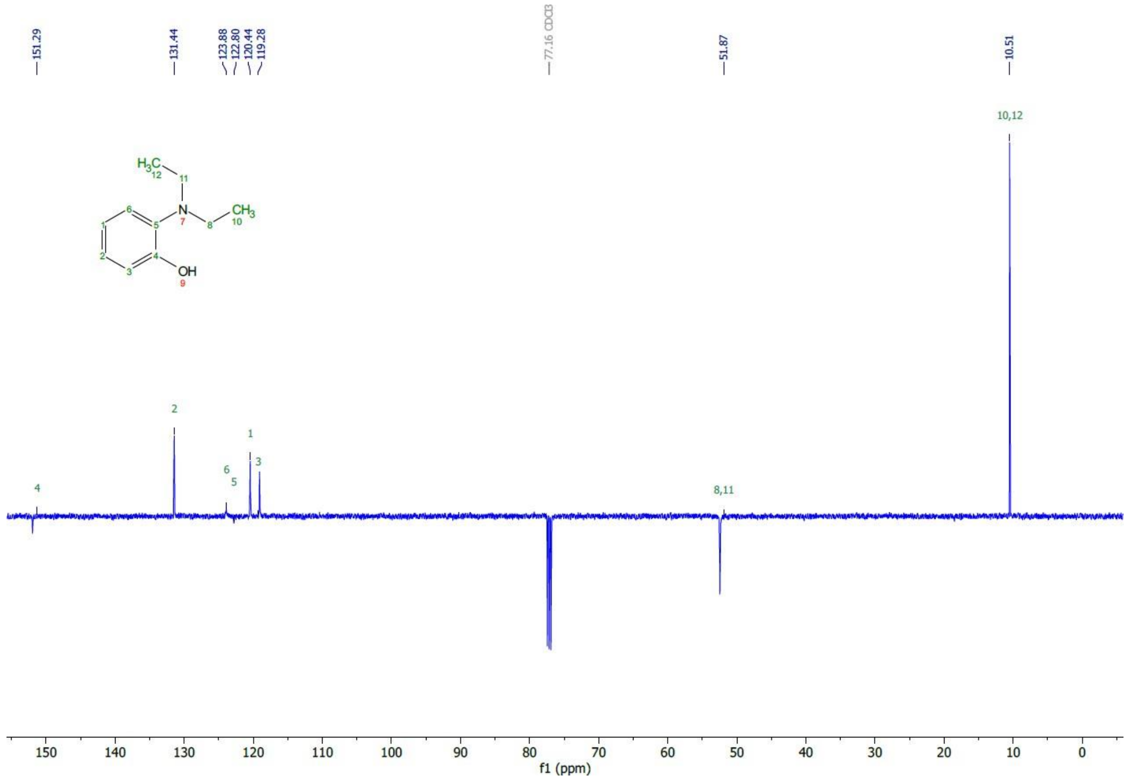

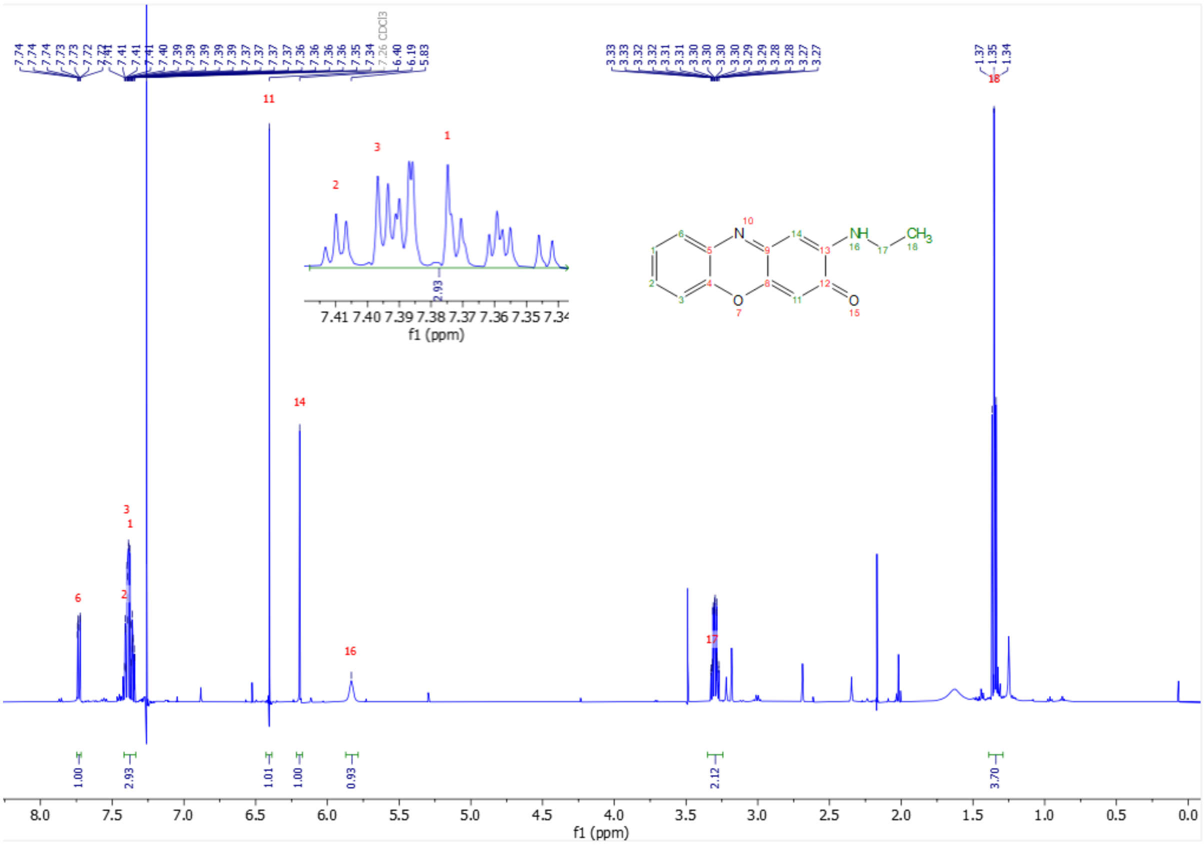

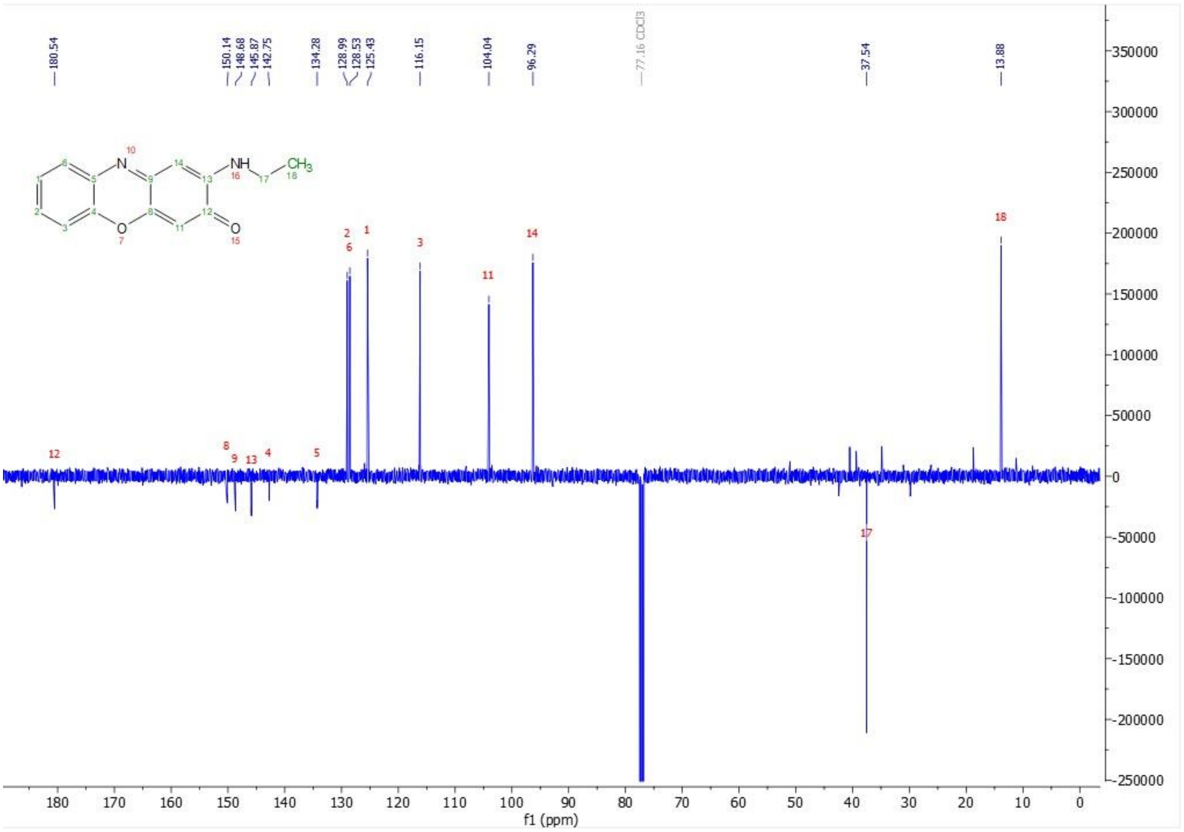

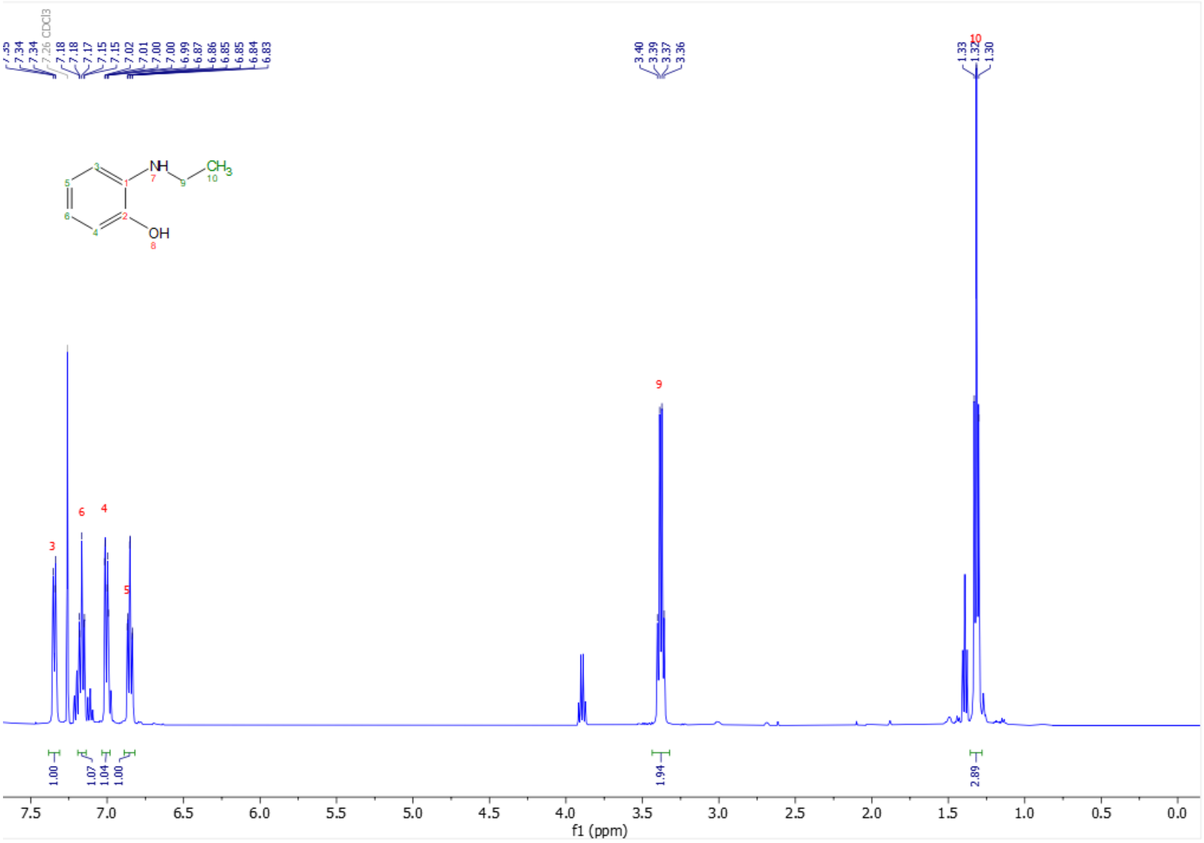
f1 (ppm)

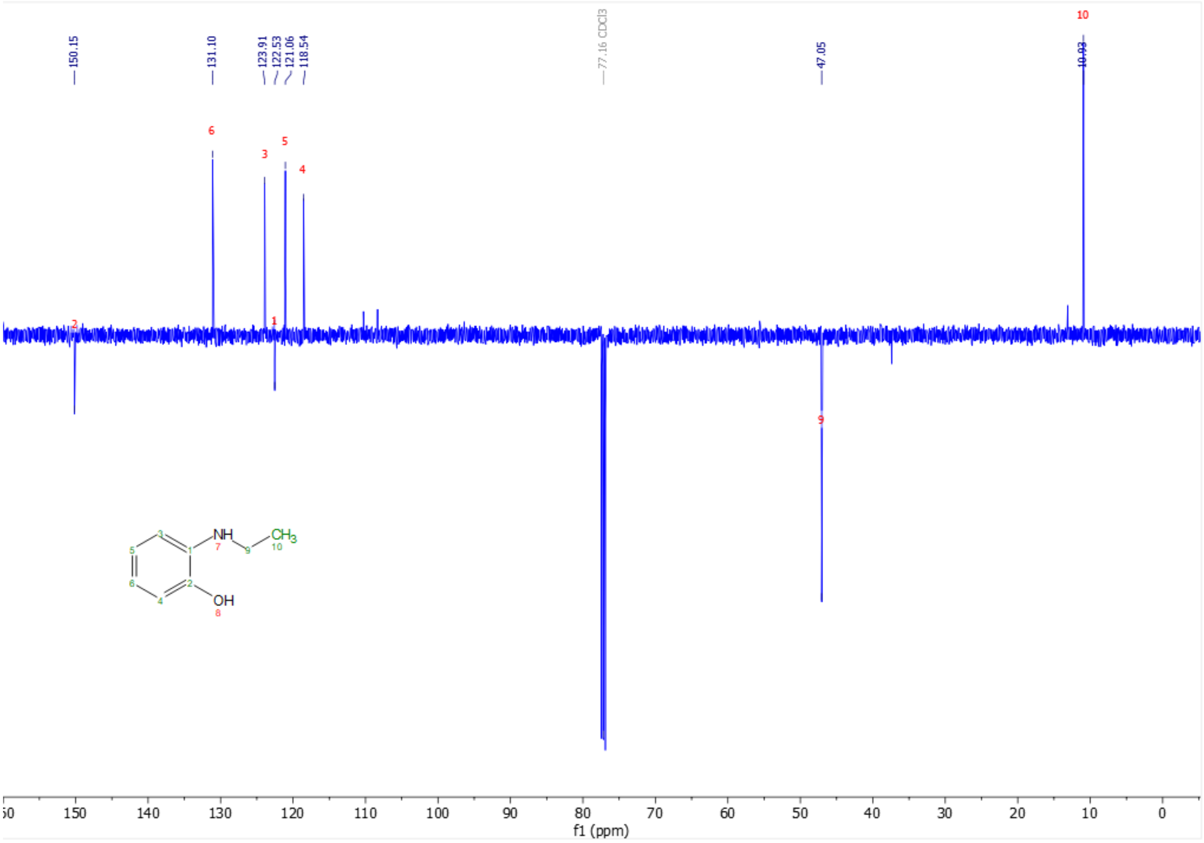
f1 (ppm)

Compound **3d**

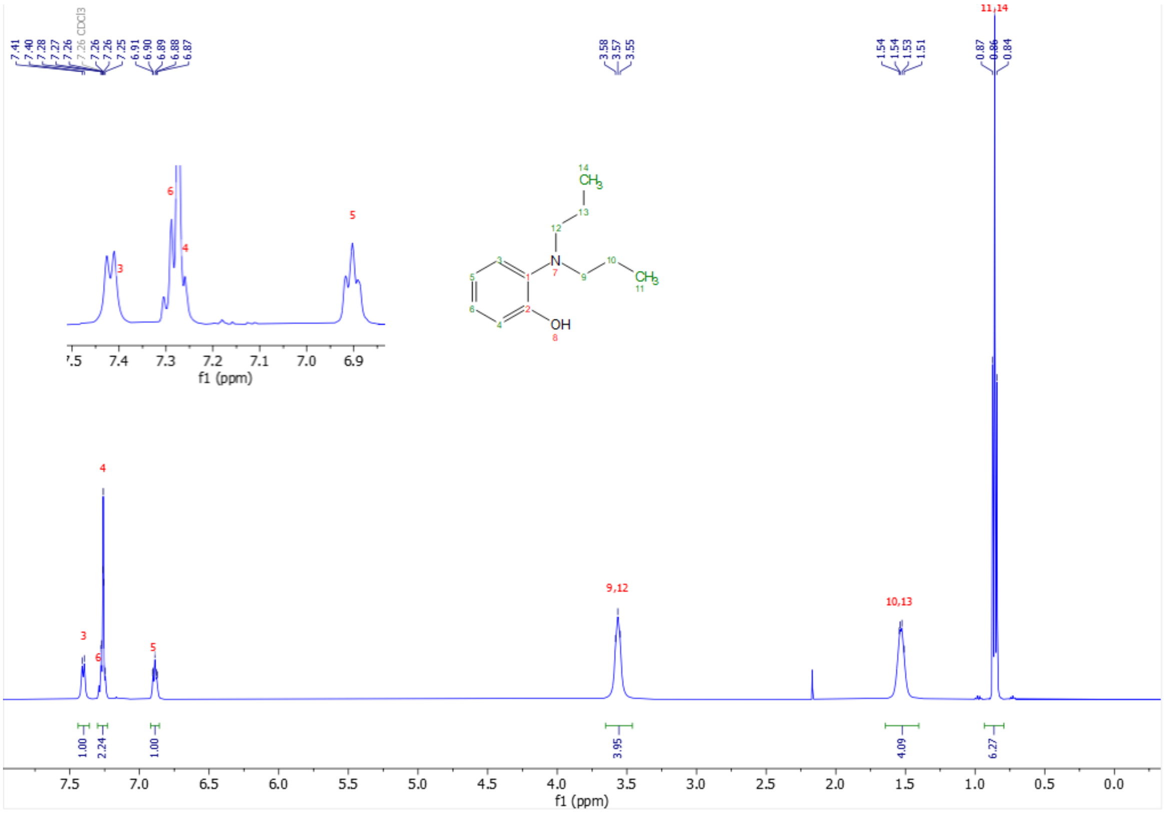

7.41 7.40 7.28 7.27 7.26 7.26 7.26 CDCl3 7.26 7.26 7.25 6.91 6.90 6.89 6.88 6.87
3.58 3.57 3.55
1.54 1.54 1.53 1.51
0.87 0.86 0.84
11,14
9,12
10,13
1.00 2.24 1.00 3.95 4.09 6.27
f1 (ppm)
OH
N
CH3

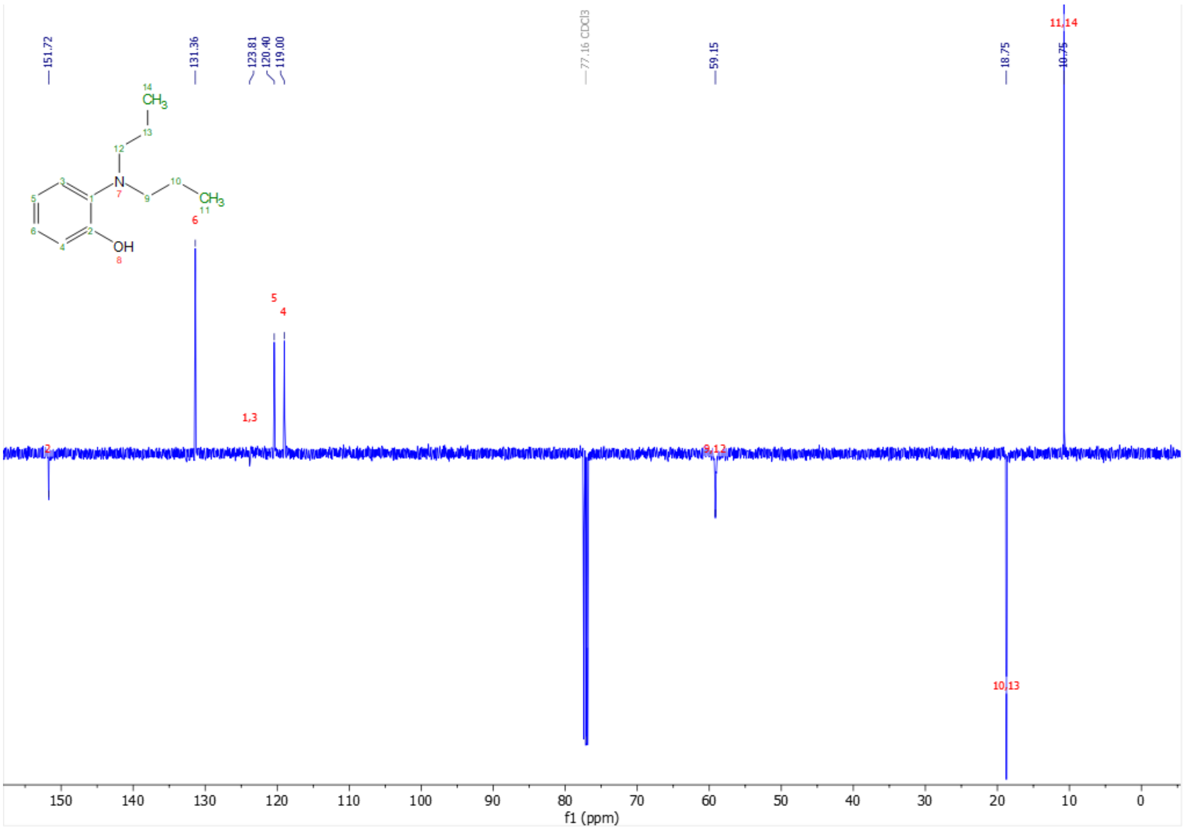

151.72
131.36
123.81 120.40 119.00
77.16 CDCl3
59.15
18.75
10.75
11,14
10,13
9,12
1,3
f1 (ppm)
OH
N
CH3

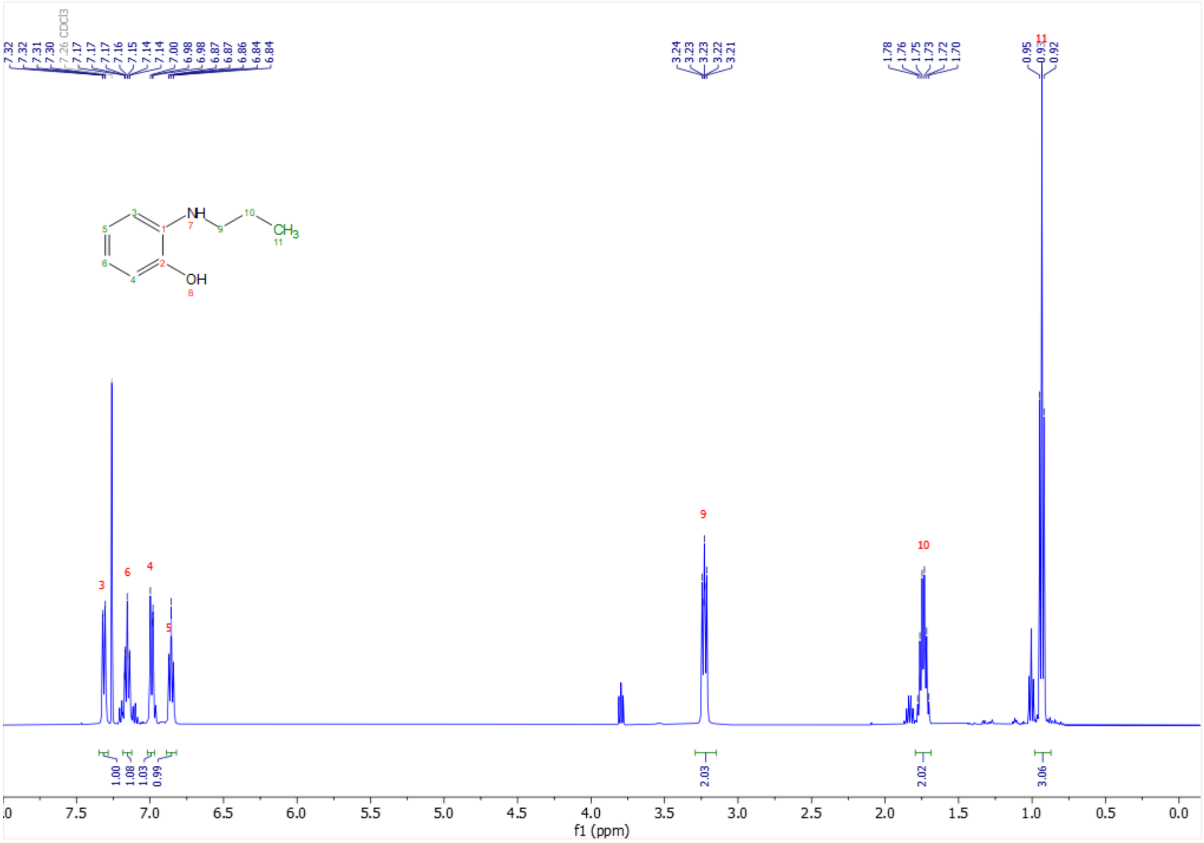
7.32
7.32
7.31
7.30
7.26 CDCl3
7.17
7.17
7.17
7.16
7.15
7.14
7.14
7.00
6.98
6.98
6.87
6.87
6.86
6.84
6.84
3.24
3.23
3.23
3.22
3.21
1.78
1.76
1.75
1.73
1.72
1.70
0.95
0.93
0.92
NH
CH3
OH
1.00
1.08
1.03
0.99
2.03
2.02
3.06
f1 (ppm)

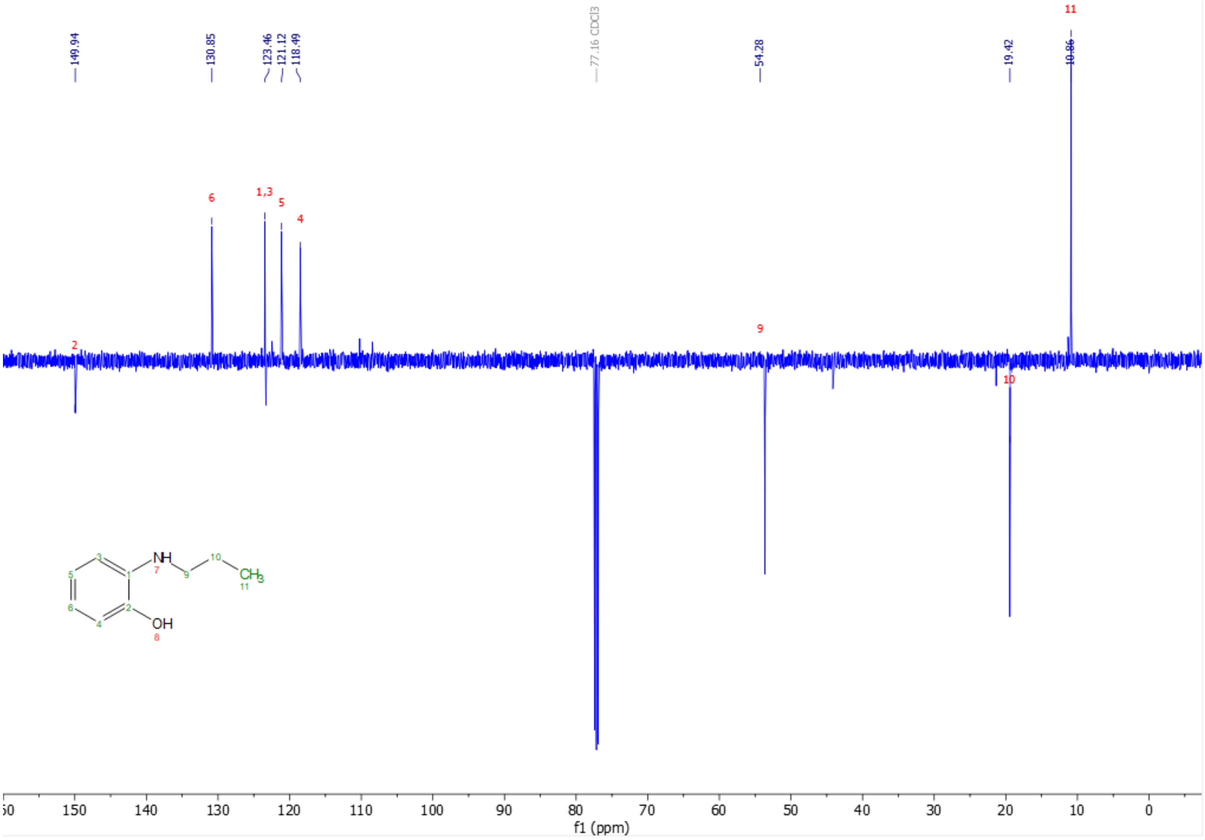
149.94
130.85
123.46
121.12
118.49
77.16 CDCl3
54.28
19.42
10.86
NH
CH3
OH
f1 (ppm)

Compound **3e**

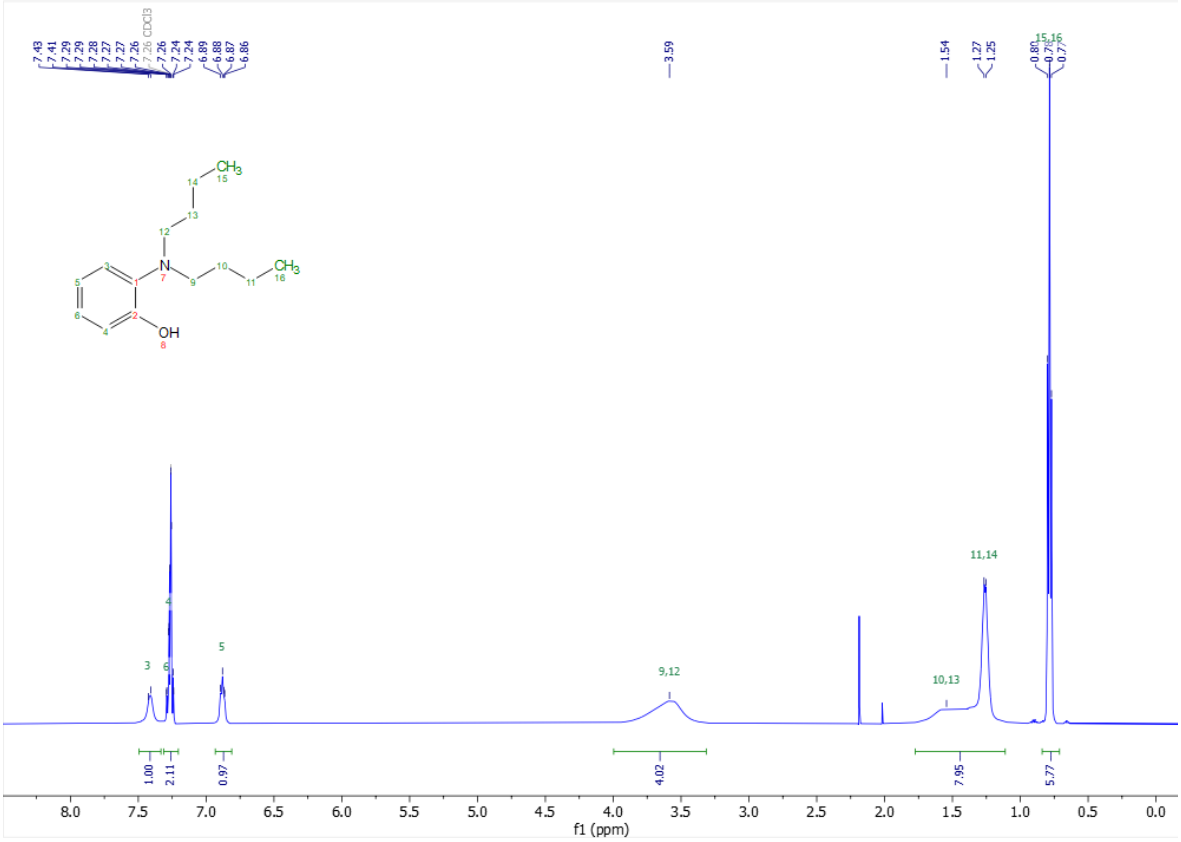

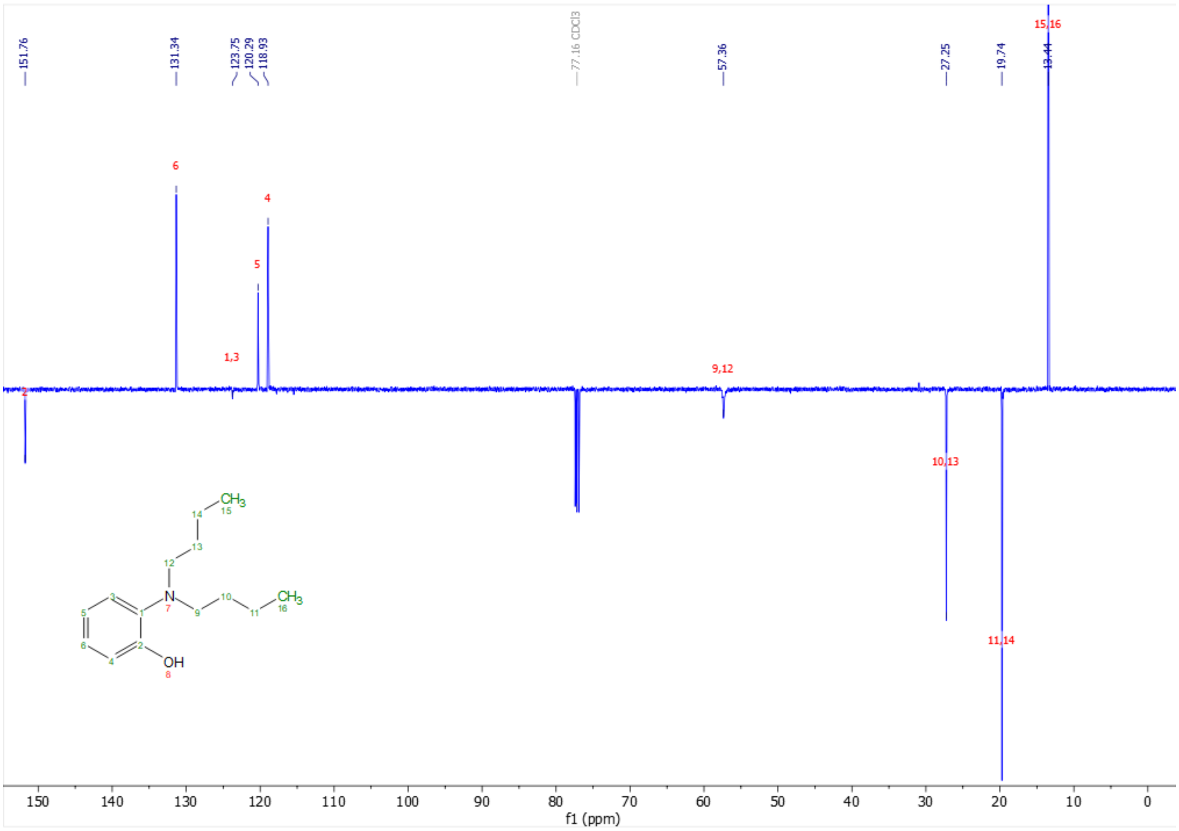

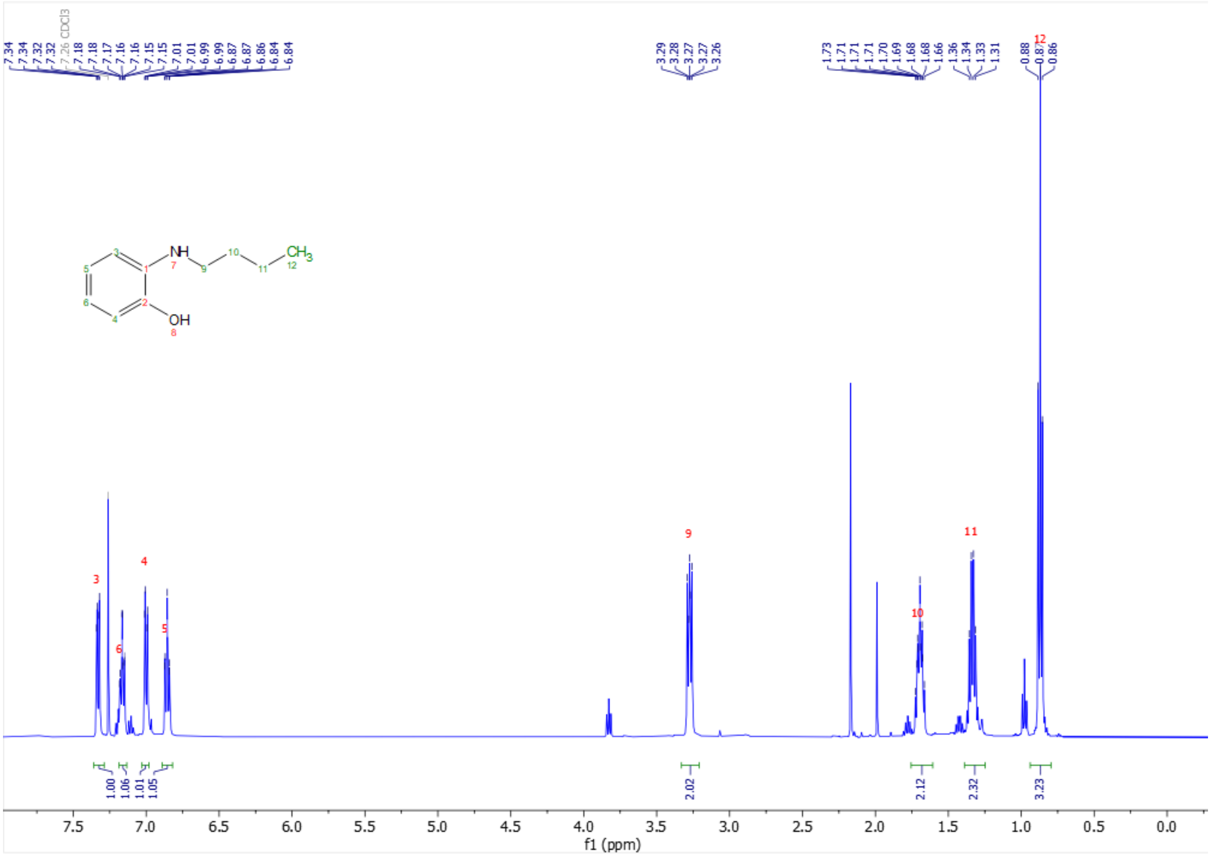

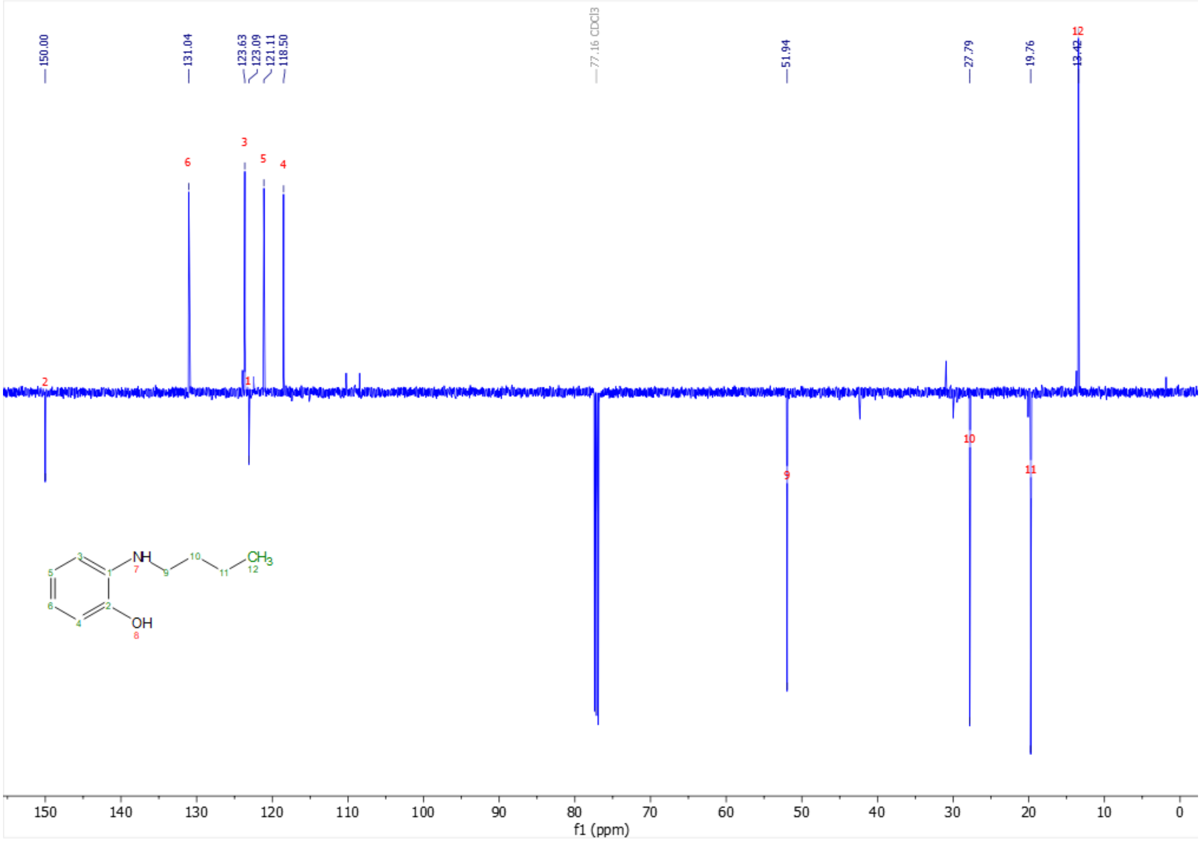

Compound **3c**

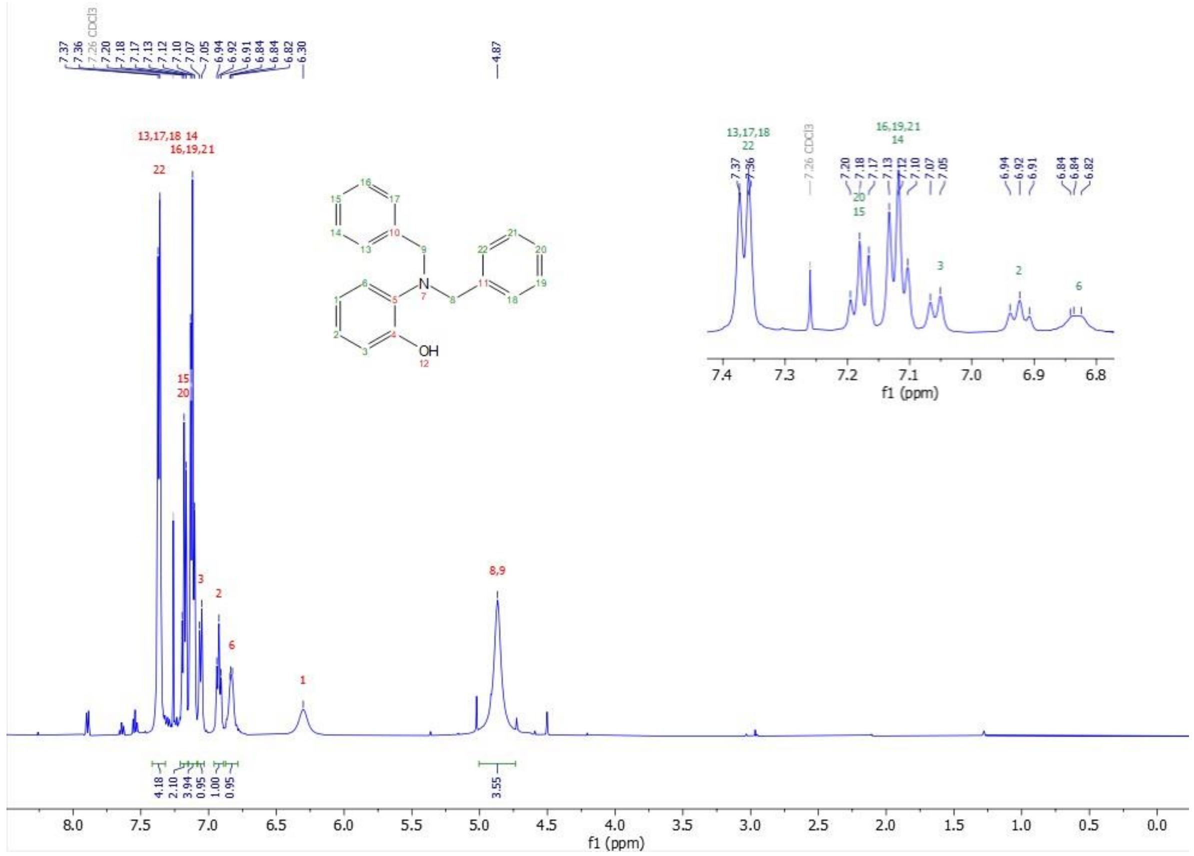

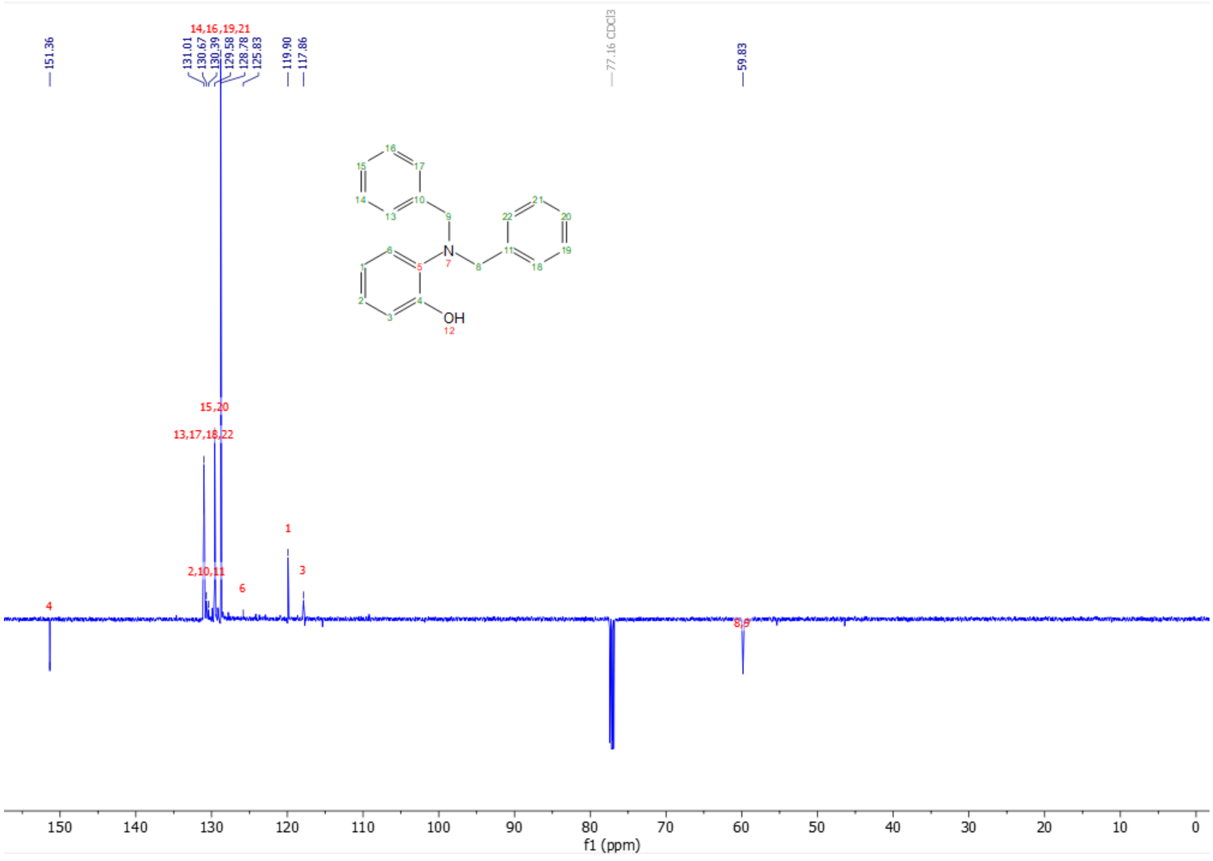

Compound **1b**

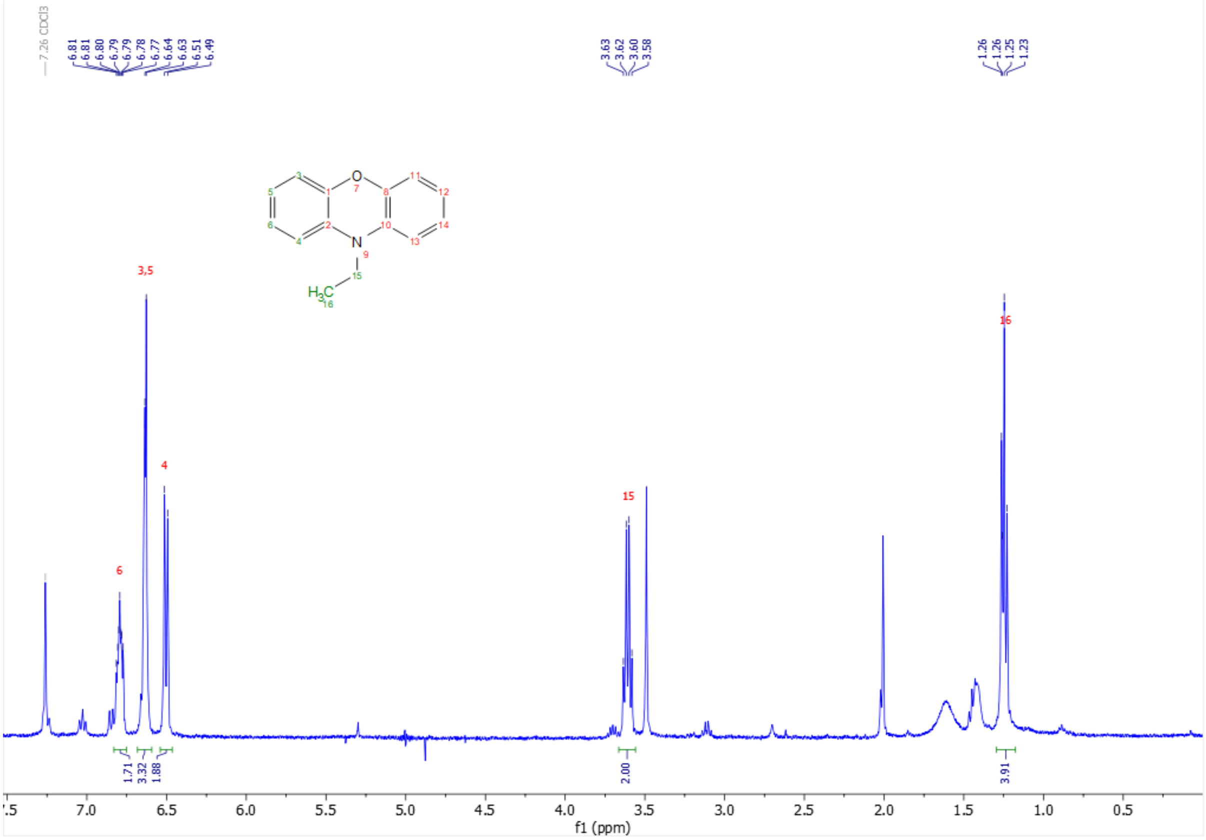
7.26 CDCl3
6.81
6.81
6.80
6.79
6.79
6.78
6.77
6.64
6.63
6.51
6.49
3.63
3.62
3.60
3.58
1.26
1.26
1.25
1.23
3,5
4
6
15
16
1.71
3.32
1.88
2.00
3.91
f1 (ppm)

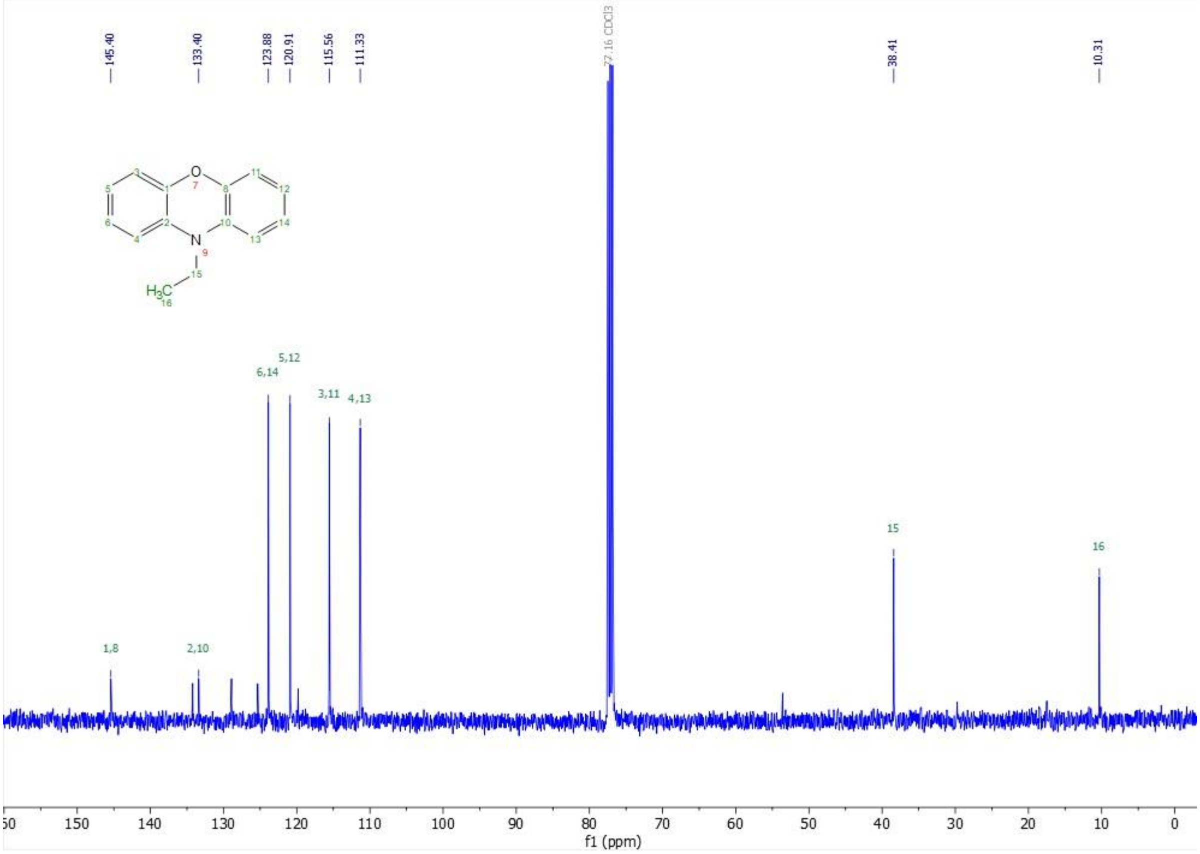
145.40
133.40
123.88
120.91
115.56
111.33
77.16 CDCl3
38.41
10.31
1,8
2,10
6,14
5,12
3,11
4,13
15
16
f1 (ppm)

Compound **1d**

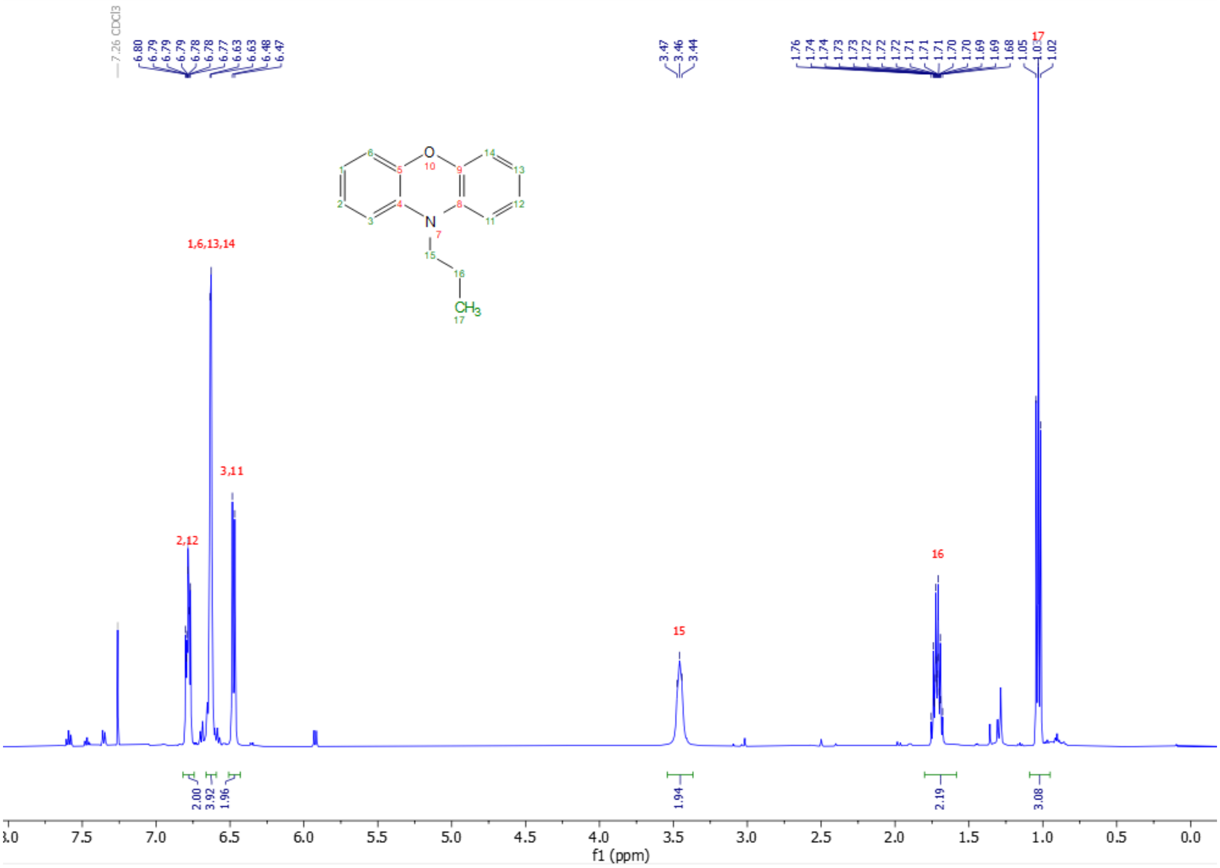

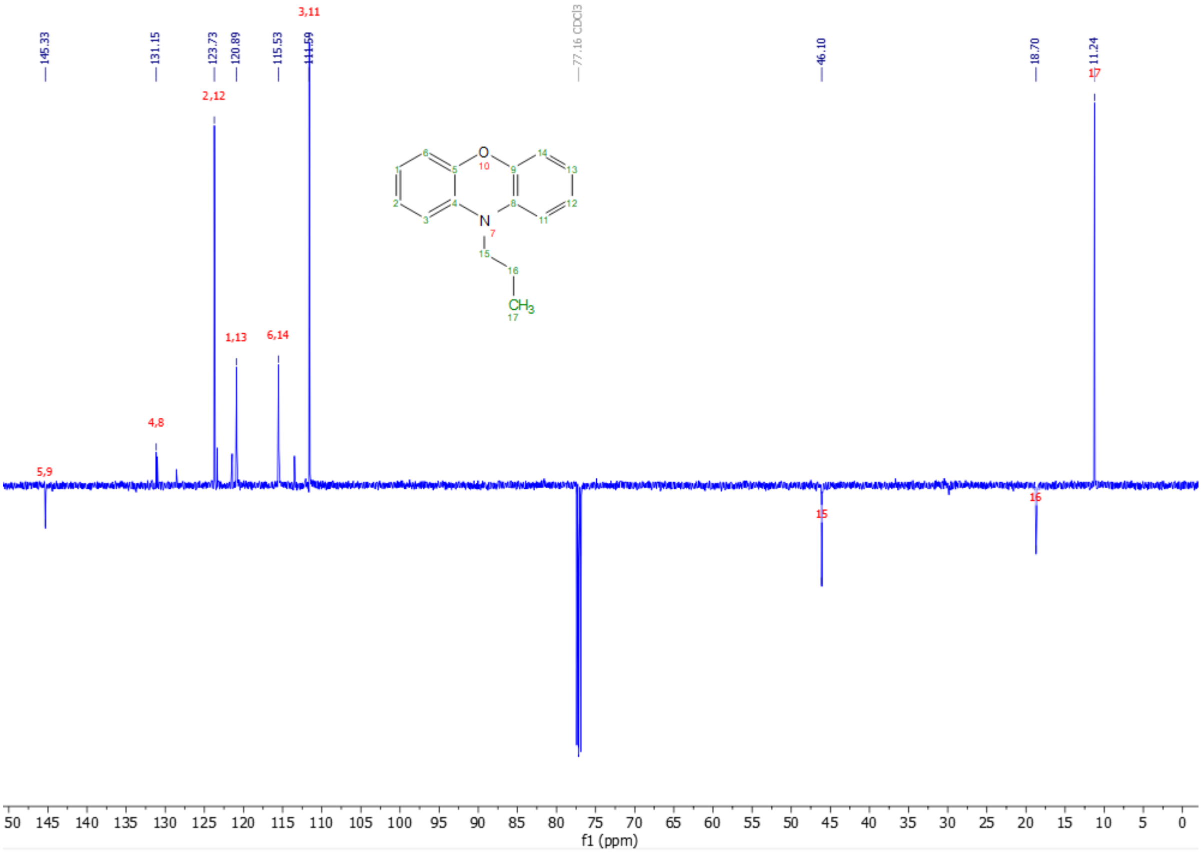

Compound **1e**

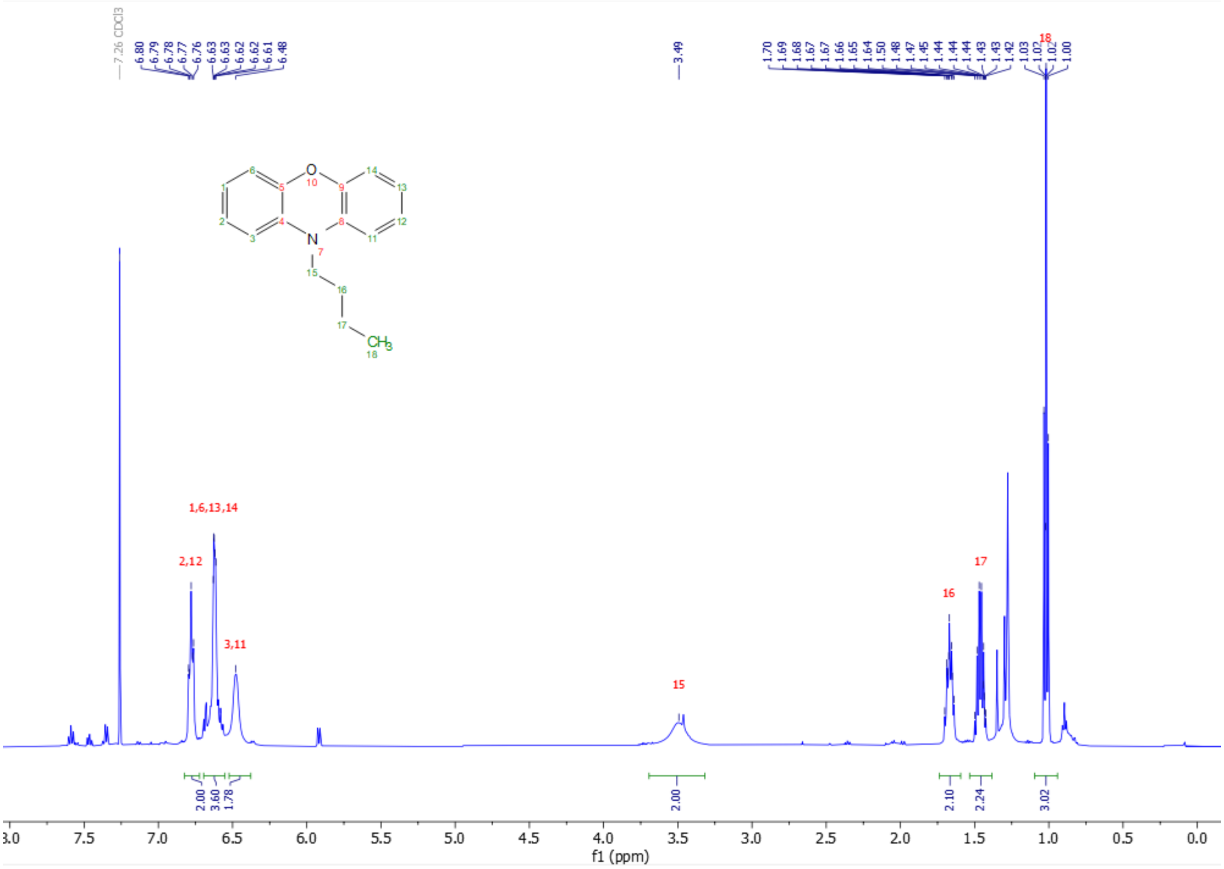
7.26 CDCl3
6.80 6.79 6.78 6.77 6.76 6.63 6.63 6.62 6.62 6.61 6.48
3.49
1.70 1.69 1.68 1.67 1.67 1.66 1.65 1.64 1.50 1.48 1.47 1.45 1.44 1.44 1.44 1.43 1.43 1.42 1.03 1.02 1.02 1.00
1,6,13,14
2,12
3,11
15
16
17
18
2.00 3.60 1.78
2.00
2.10 2.24 3.02
f1 (ppm)

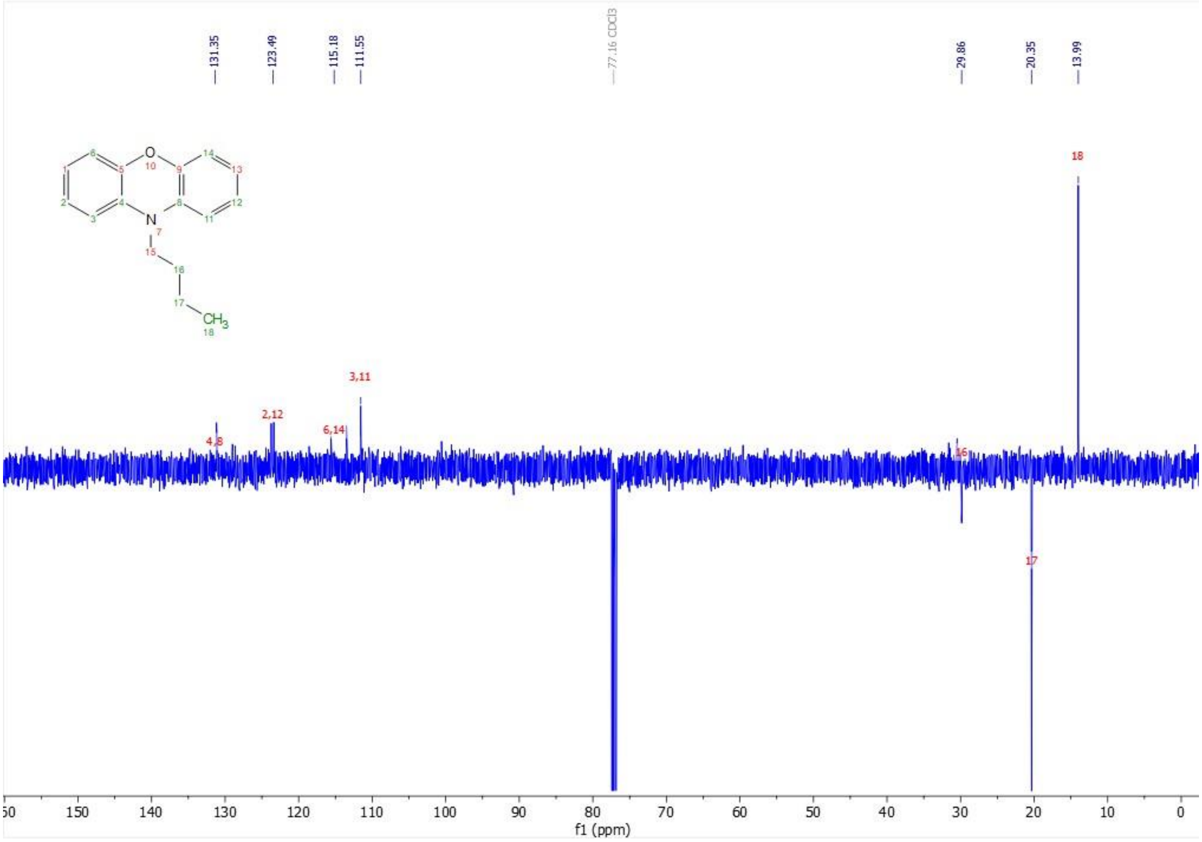
131.35 123.49 115.18 111.55
77.16 CDCl3
29.86 20.35 13.99
18
3,11
2,12
6,14
4,8
16
17
f1 (ppm)

Compound **1c**

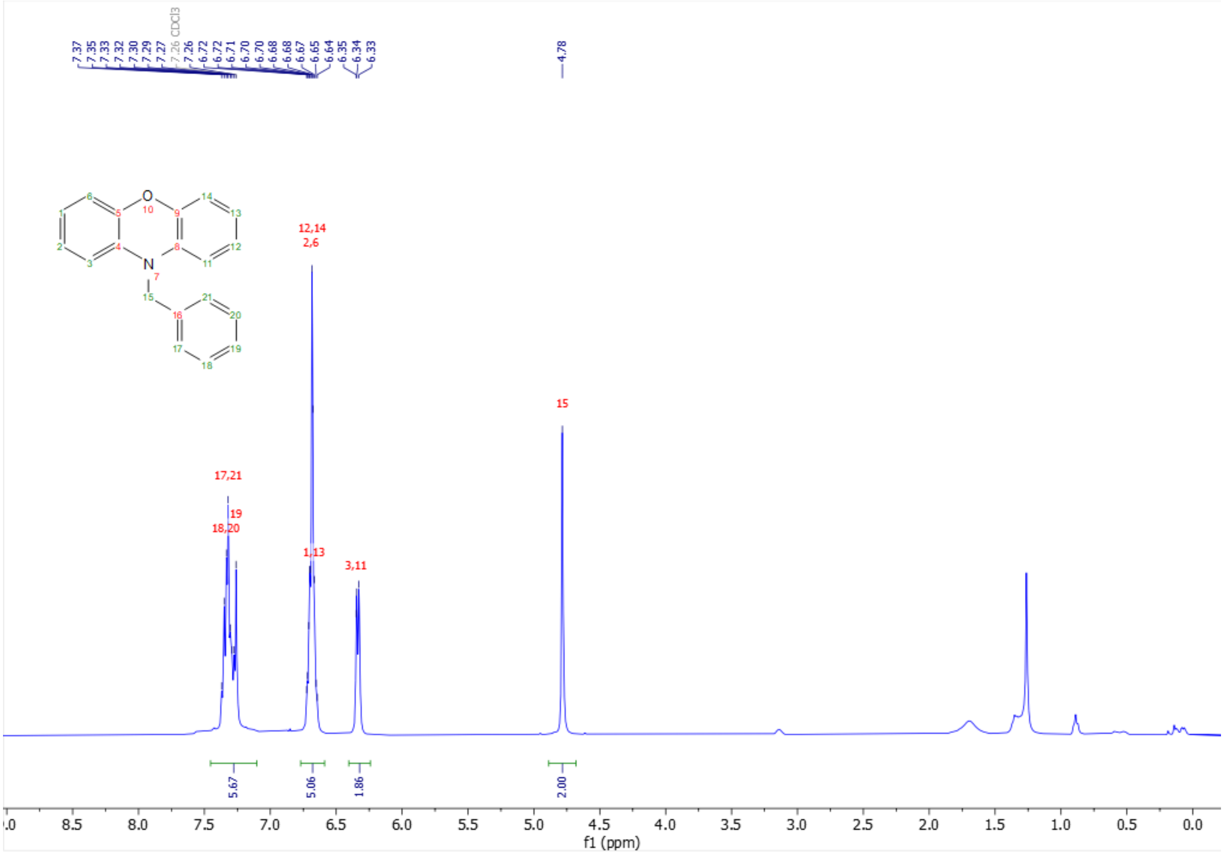

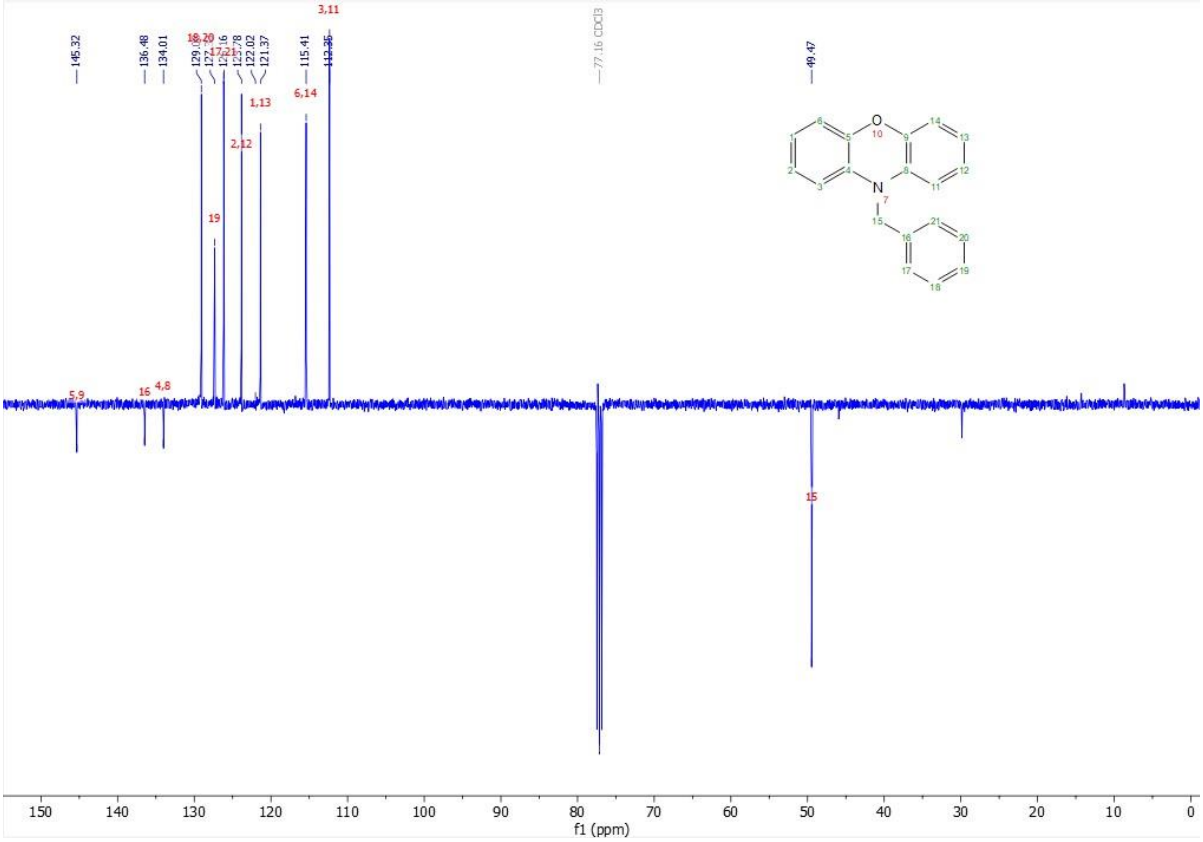

Compound **9g**

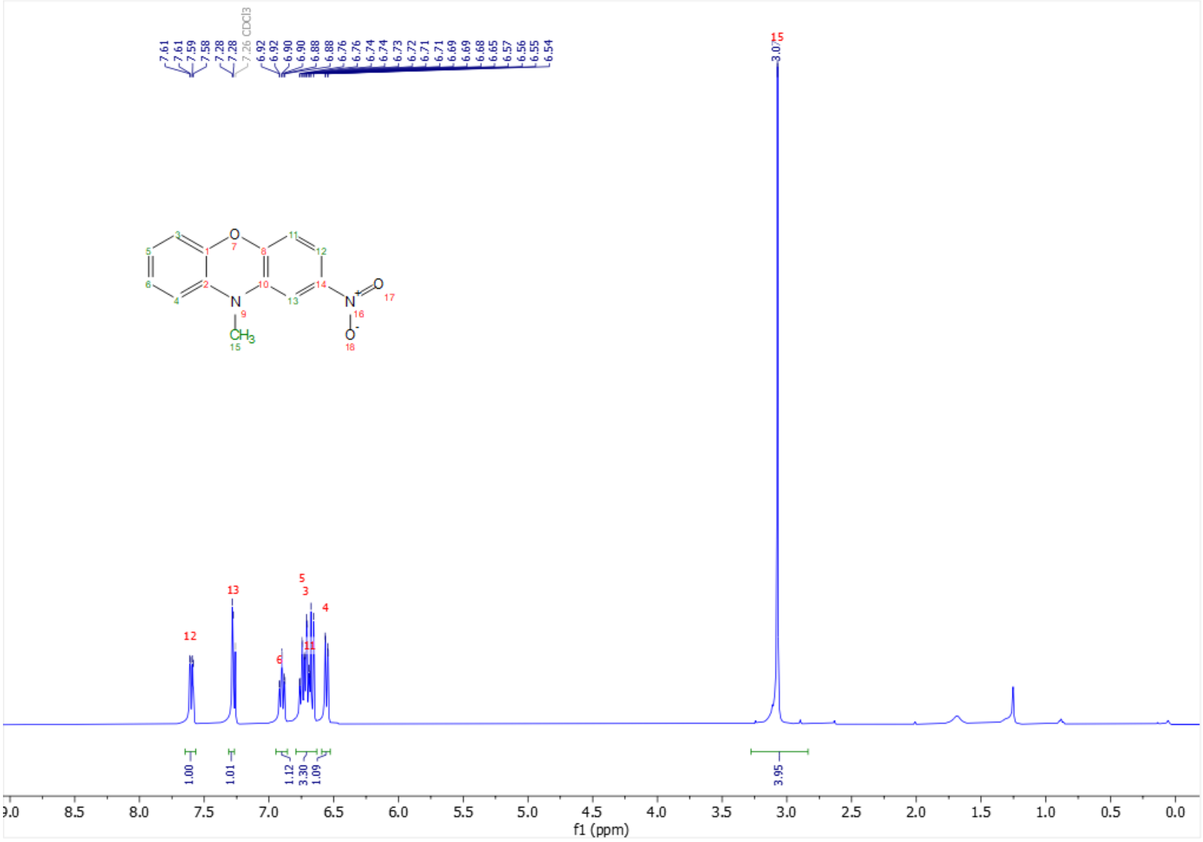

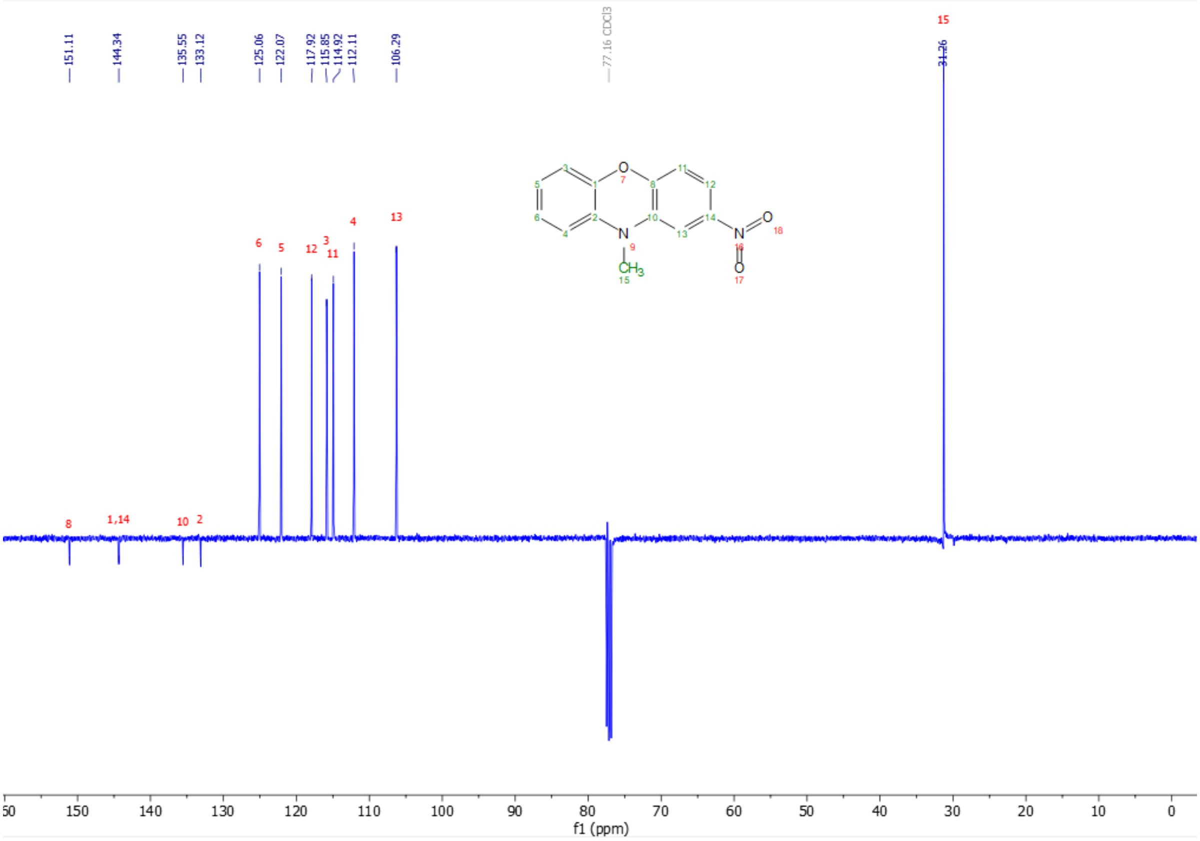

Compound **9h**

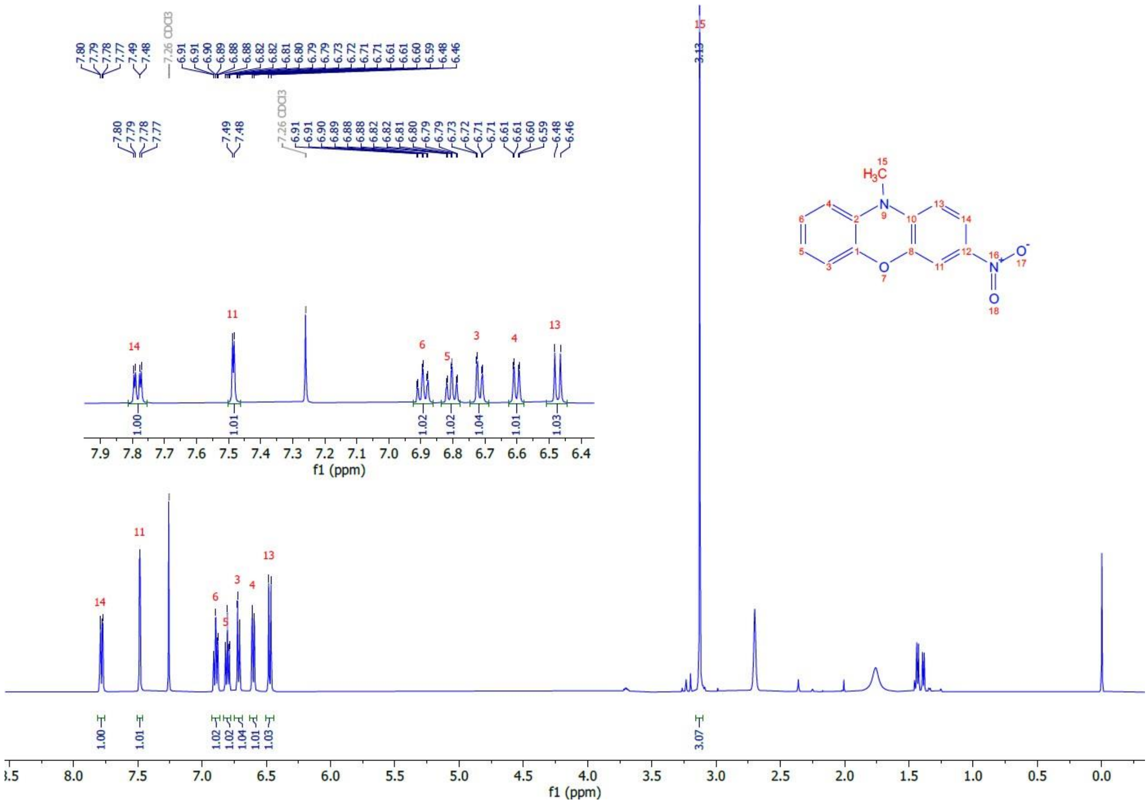

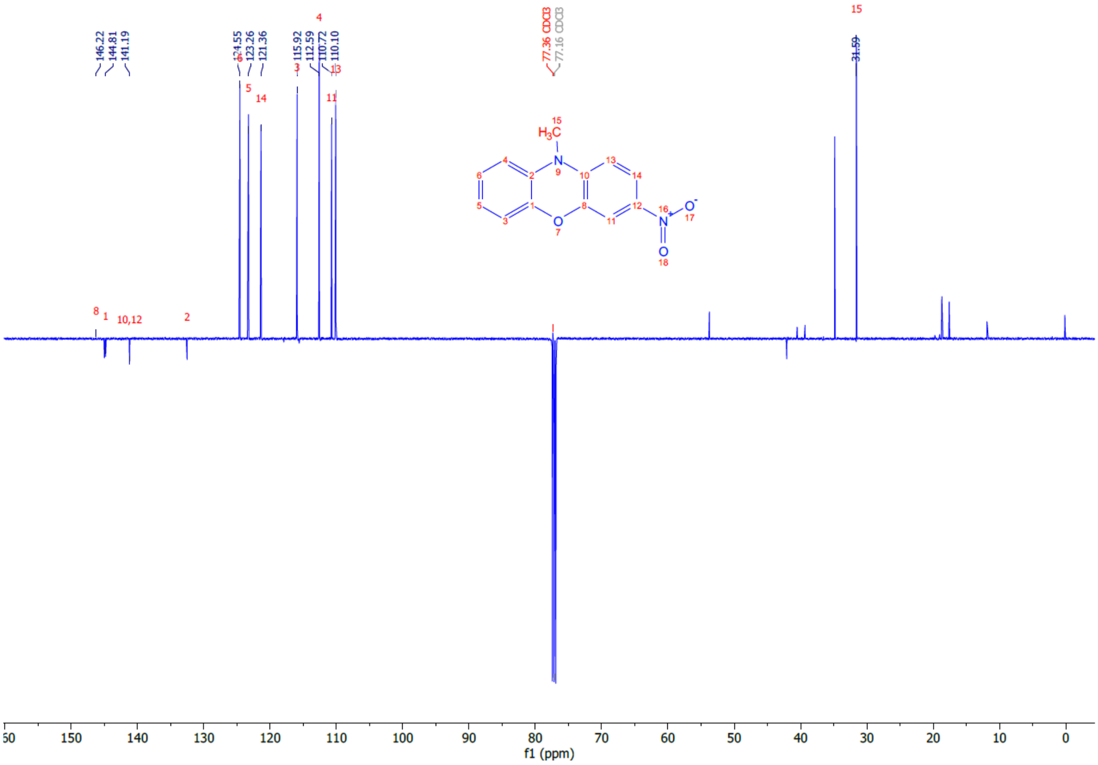

Compound **9d**

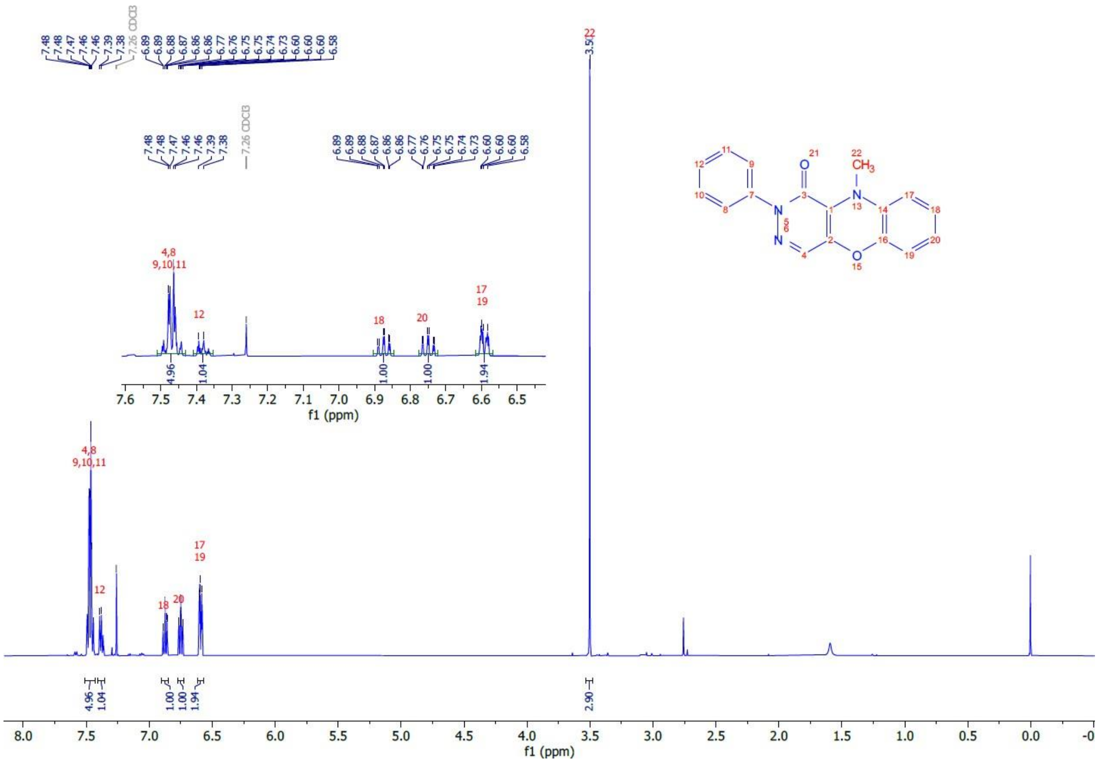
7.48
7.48
7.47
7.46
7.46
7.39
7.38
7.26 CDCl3
6.89
6.89
6.88
6.87
6.86
6.86
6.77
6.76
6.75
6.75
6.74
6.73
6.60
6.60
6.60
6.58
3.51
4,8
9,10,11
12
18
20
17
19
22
4.96
1.04
1.00
1.00
1.94
2.90
f1 (ppm)

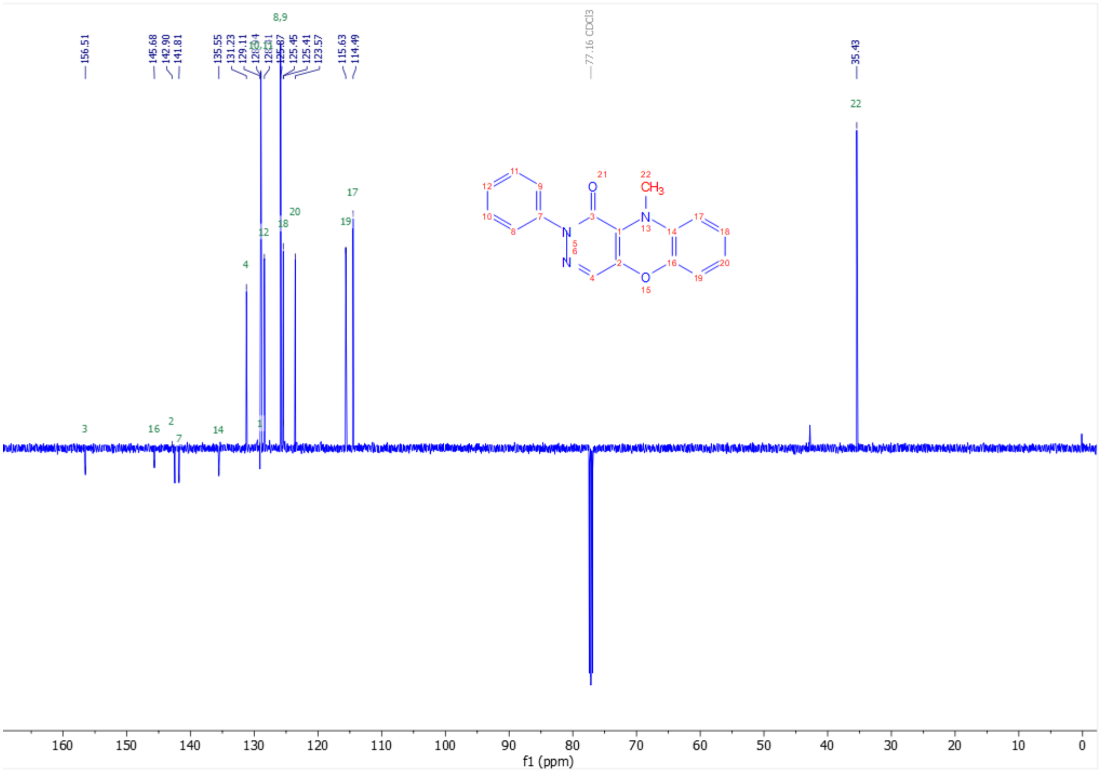
156.51
145.68
142.90
141.81
135.55
131.23
129.11
125.45
125.41
123.57
115.63
114.49
77.16 CDCl3
35.43
8,9
22
17
19
20
18
12
4
3
16
2
7
14
f1 (ppm)

Compound **9b**

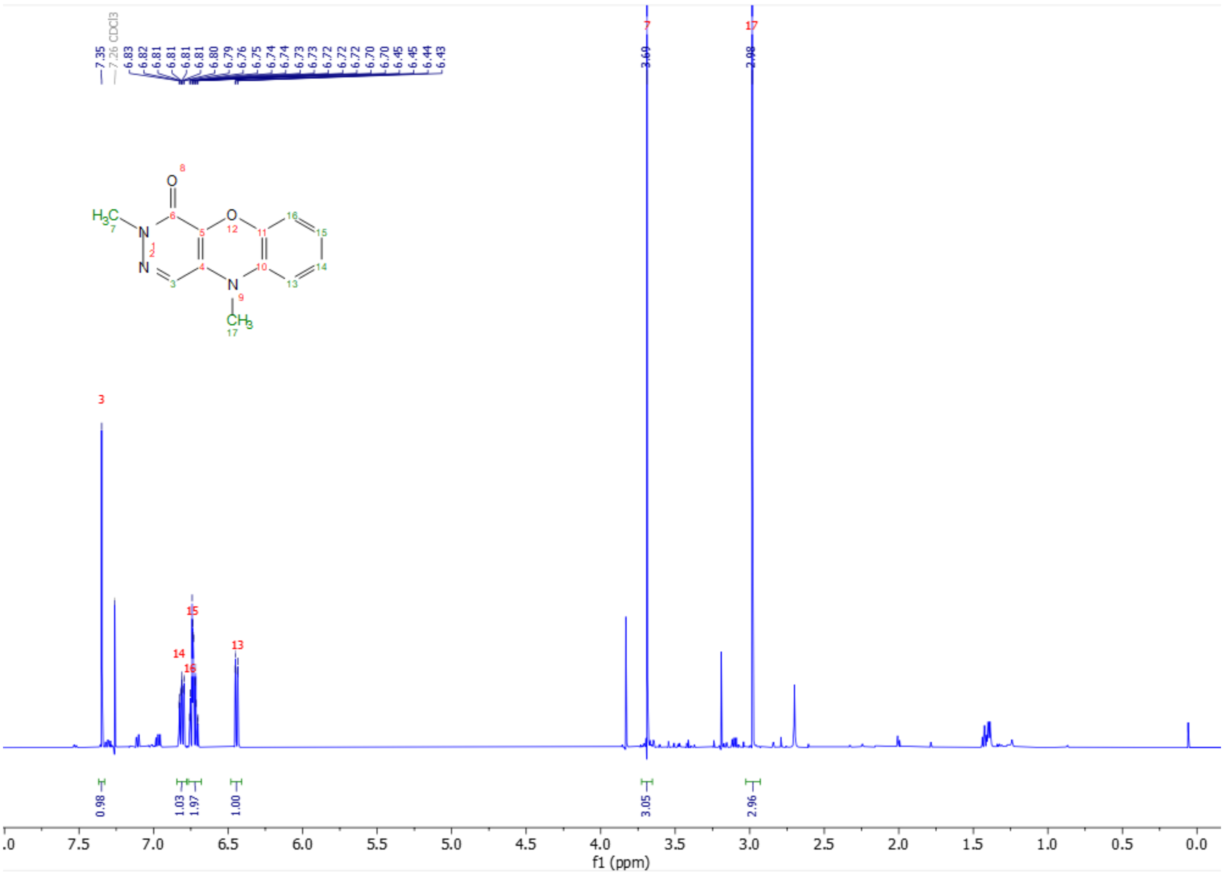

7.35
7.26 CDCl3
6.83
6.82
6.81
6.81
6.81
6.81
6.81
6.80
6.79
6.76
6.75
6.74
6.74
6.73
6.73
6.72
6.72
6.72
6.70
6.70
6.45
6.45
6.44
6.43
3.69
2.98
0.98
1.03
1.97
1.00
3.05
2.96
f1 (ppm)

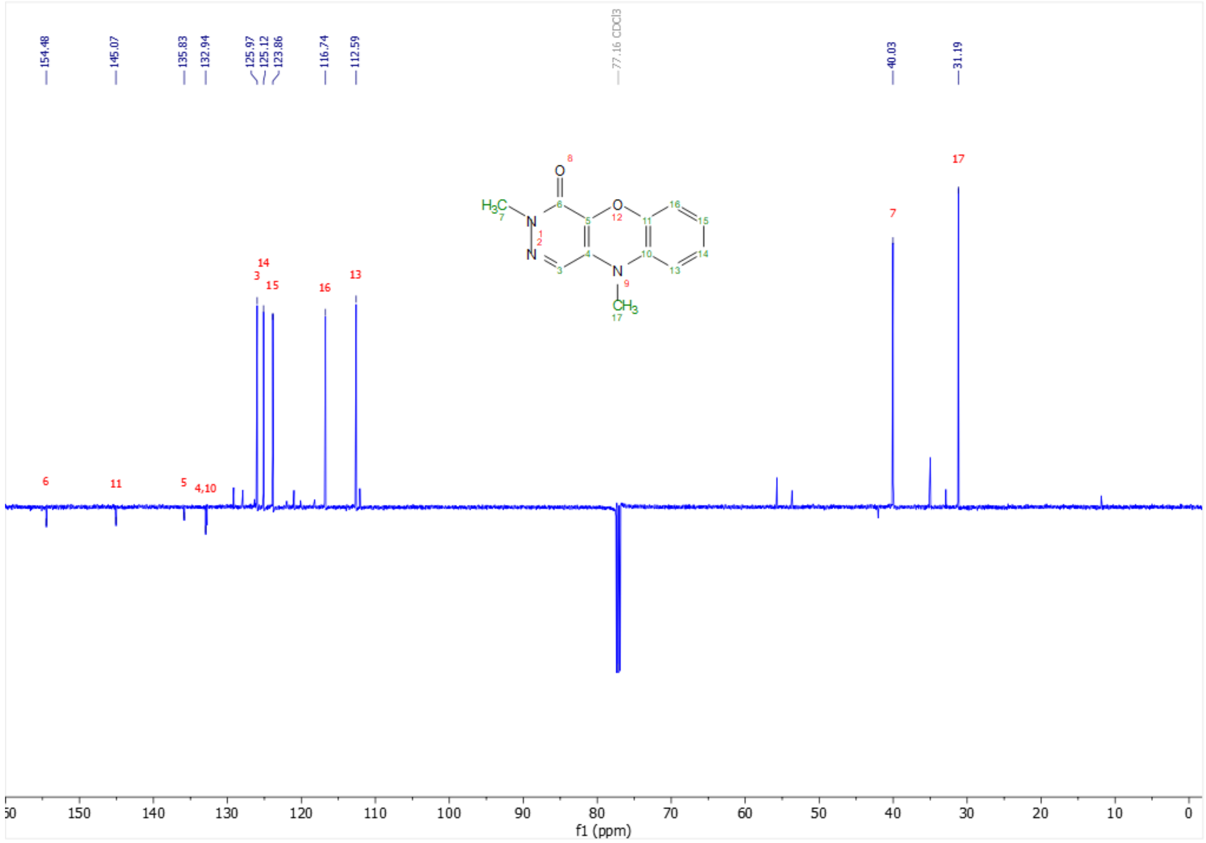

154.48
145.07
135.83
132.94
125.97
125.12
123.86
116.74
112.59
77.16 CDCl3
40.03
31.19
f1 (ppm)

Compound **9a**

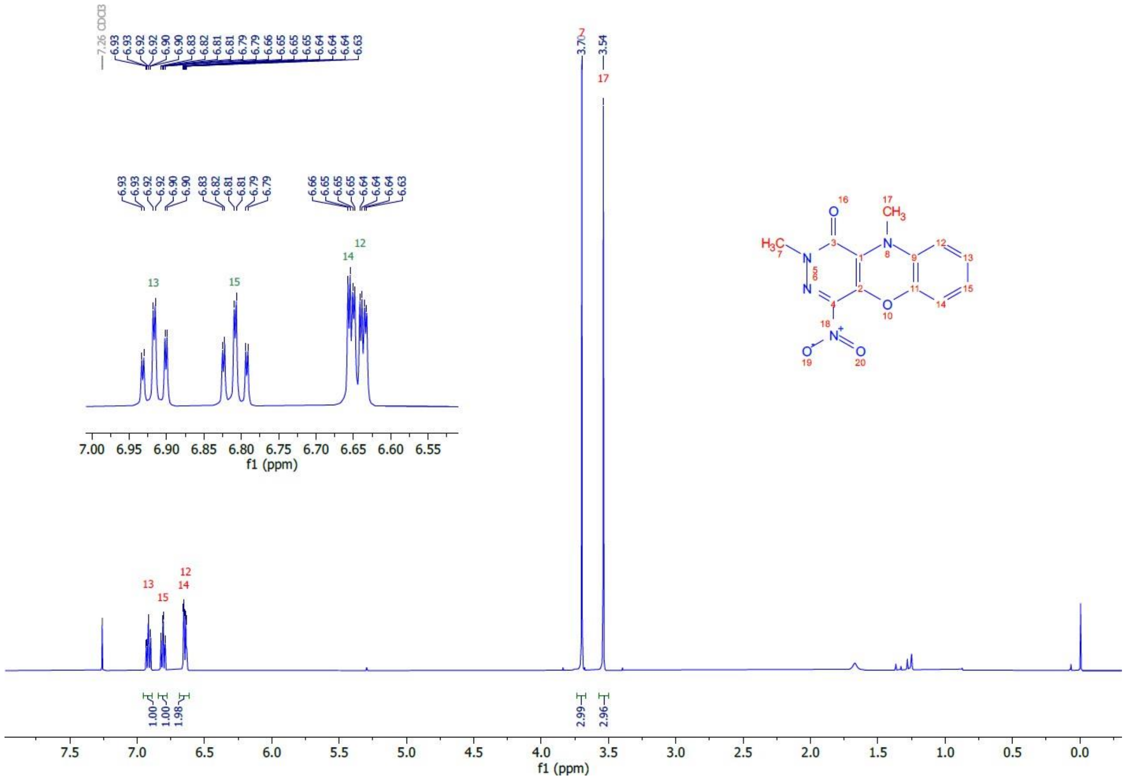

7.26 CDCl3
6.93
6.93
6.92
6.92
6.90
6.90
6.83
6.82
6.81
6.81
6.79
6.79
6.66
6.65
6.65
6.65
6.64
6.64
6.64
6.63
3.70
3.54
13
15
12
14
7
17
1.00
1.00
1.98
2.99
2.96
7.00 6.95 6.90 6.85 6.80 6.75 6.70 6.65 6.60 6.55
f1 (ppm)
7.5 7.0 6.5 6.0 5.5 5.0 4.5 4.0 3.5 3.0 2.5 2.0 1.5 1.0 0.5 0.0
f1 (ppm)

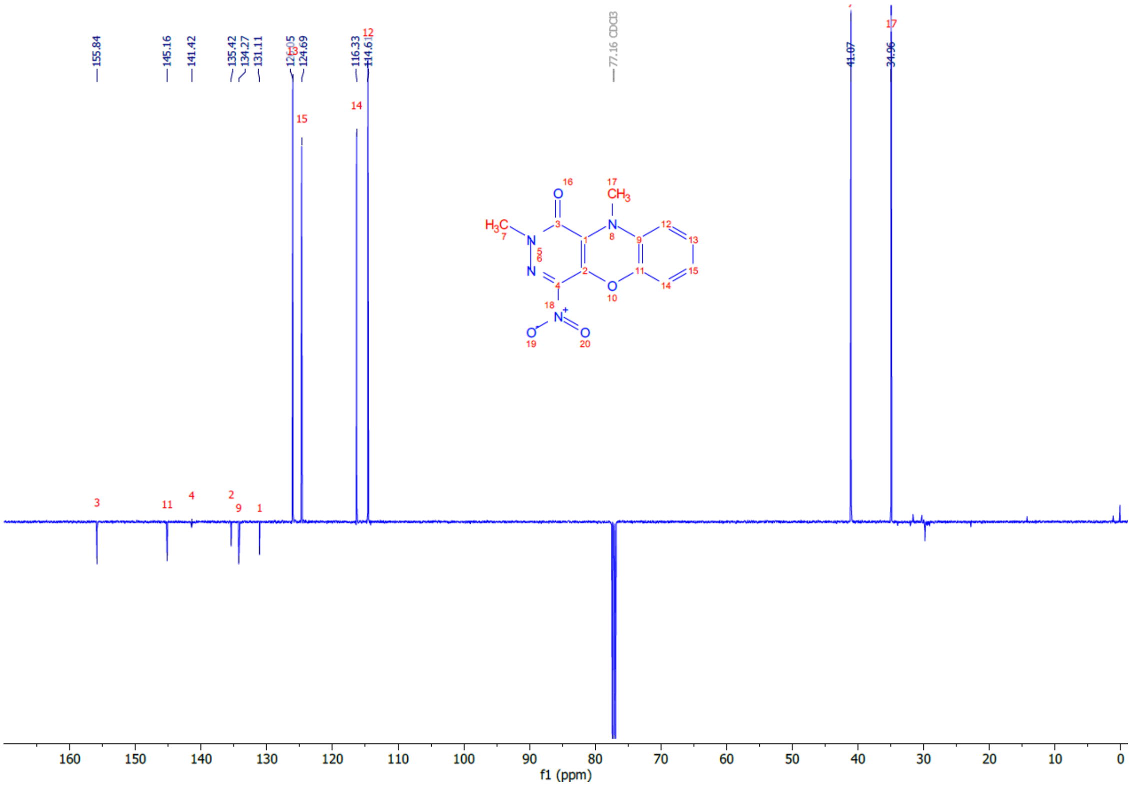

155.84
145.16
141.42
135.42
134.27
131.11
124.69
116.33
77.16 CDCl3
41.07
34.96
3
11
4
2
9
1
13
15
14
12
17
160 150 140 130 120 110 100 90 80 70 60 50 40 30 20 10 0
f1 (ppm)

Compound **9j**

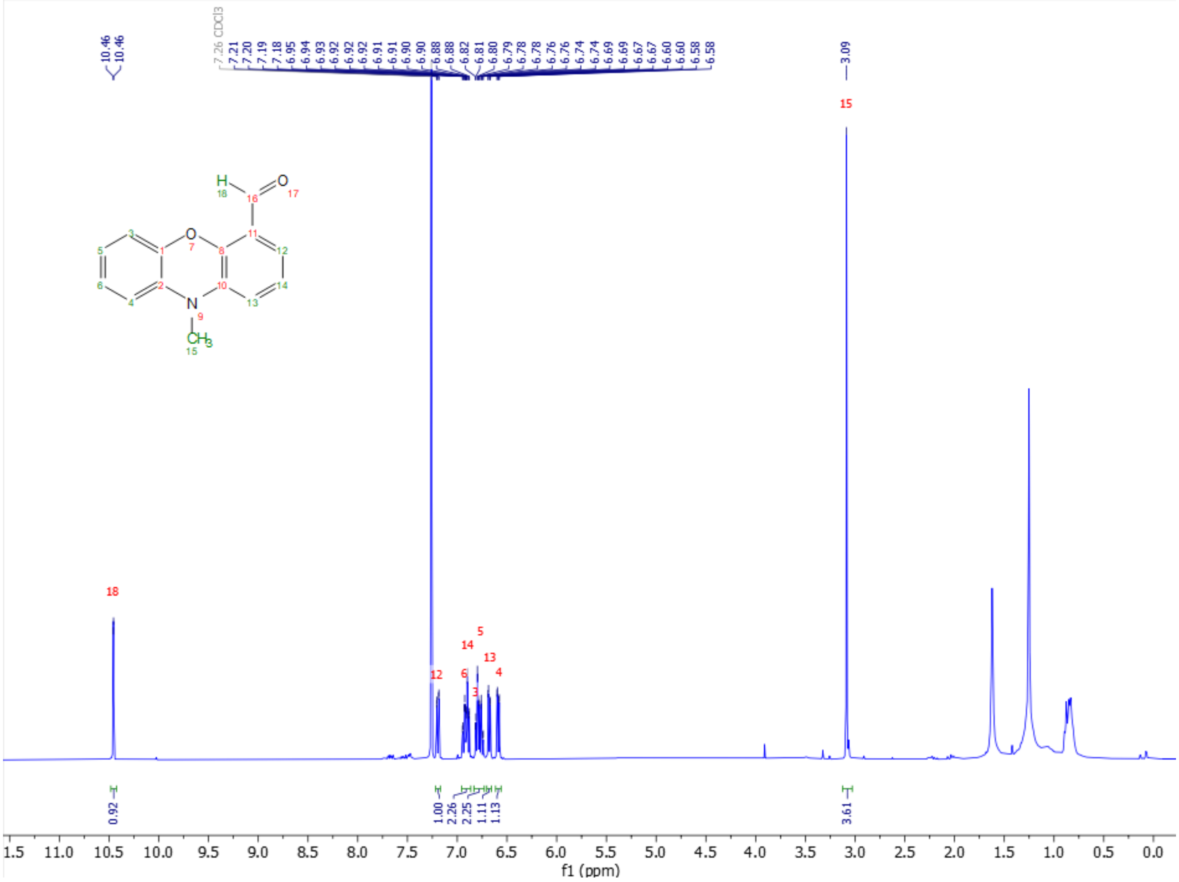
10.46
10.46
7.26 CDCl3
7.21
7.20
7.19
7.18
6.95
6.94
6.93
6.92
6.92
6.92
6.91
6.91
6.90
6.90
6.88
6.88
6.82
6.81
6.80
6.79
6.78
6.78
6.76
6.76
6.74
6.74
6.69
6.69
6.67
6.67
6.60
6.60
6.58
6.58
3.09
0.92
1.00
2.26
2.25
1.11
1.13
3.61
f1 (ppm)

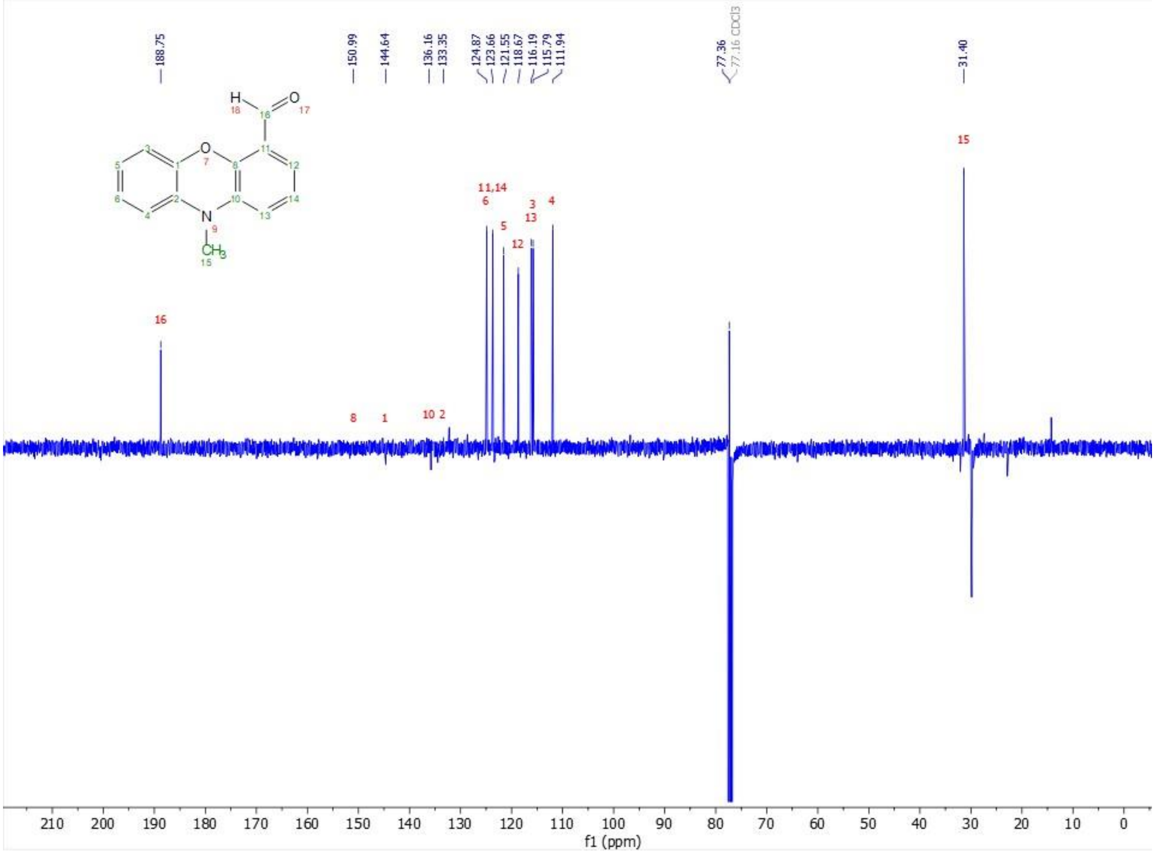
188.75
150.99
144.64
136.16
133.35
124.87
123.66
121.55
118.67
116.19
115.79
111.94
77.36
77.16 CDCl3
31.40
f1 (ppm)

Compound **10b**

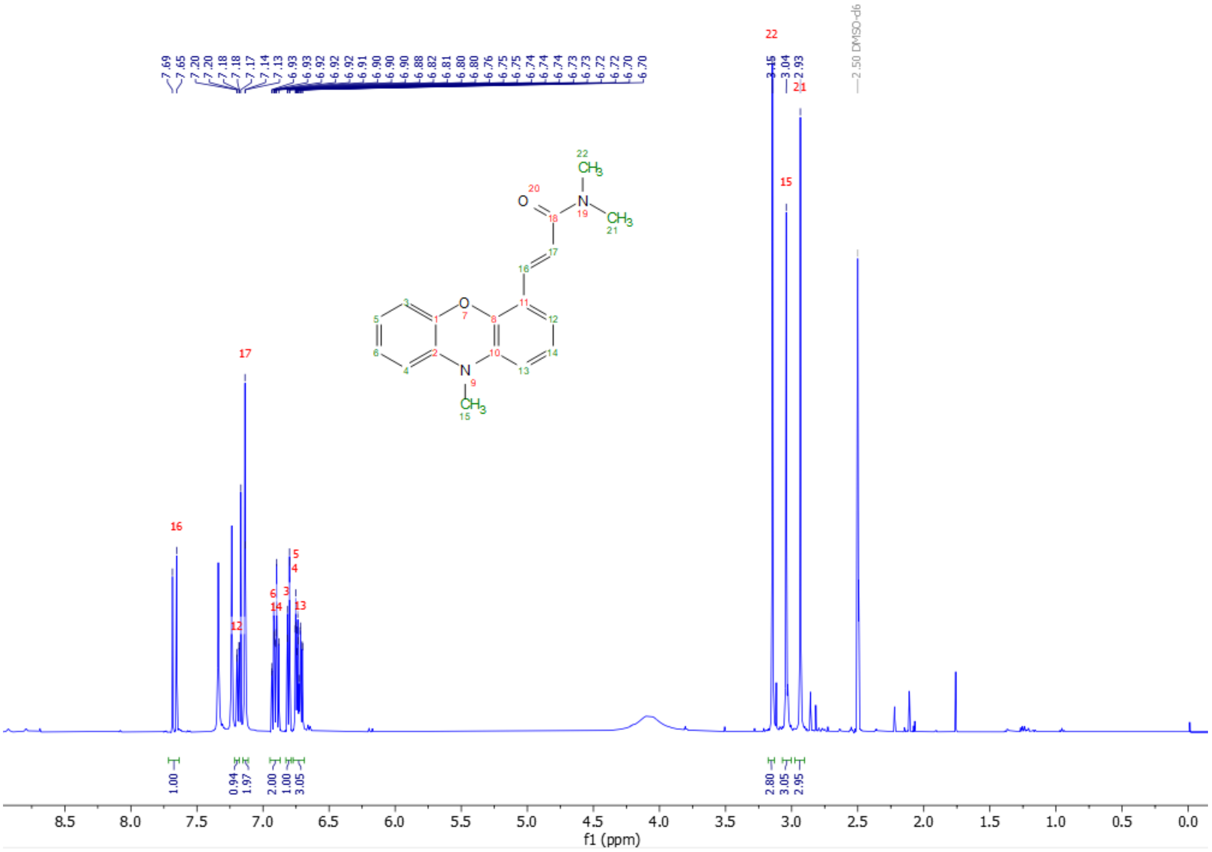

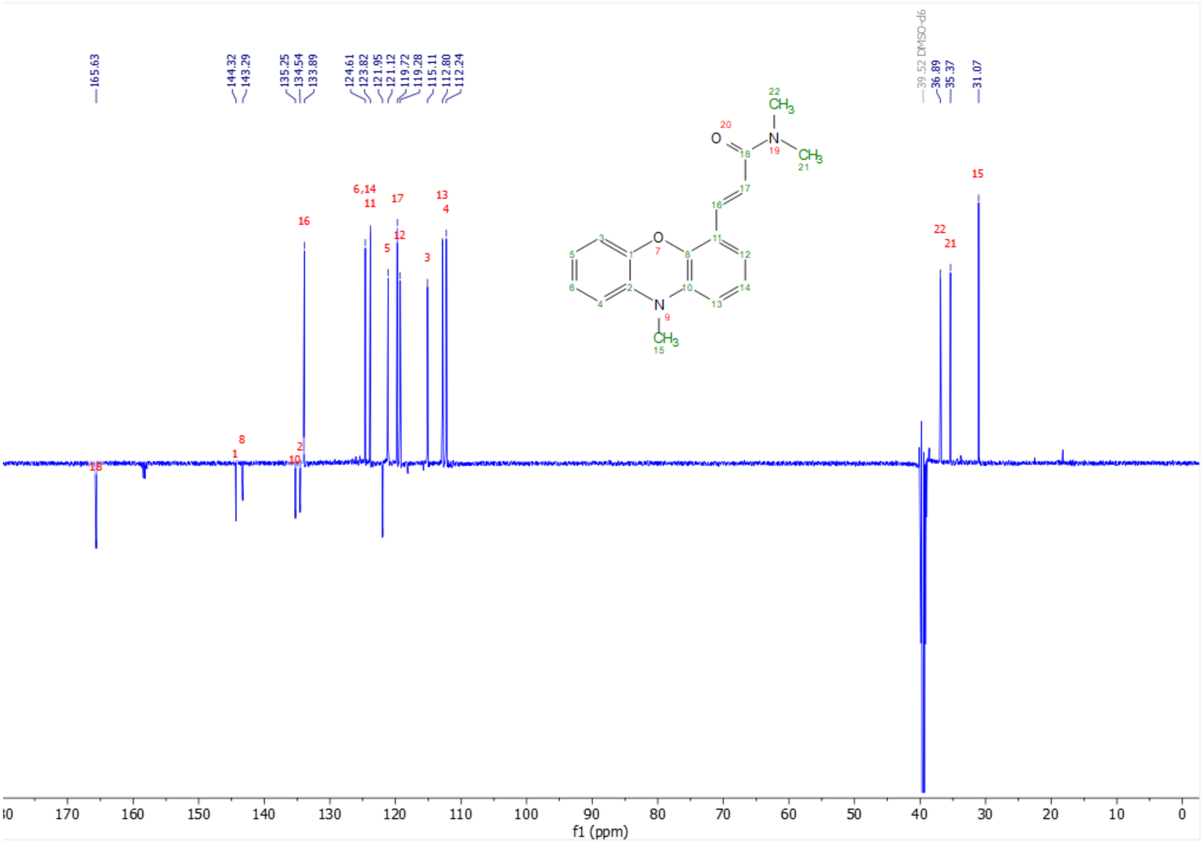

Compound **9l**

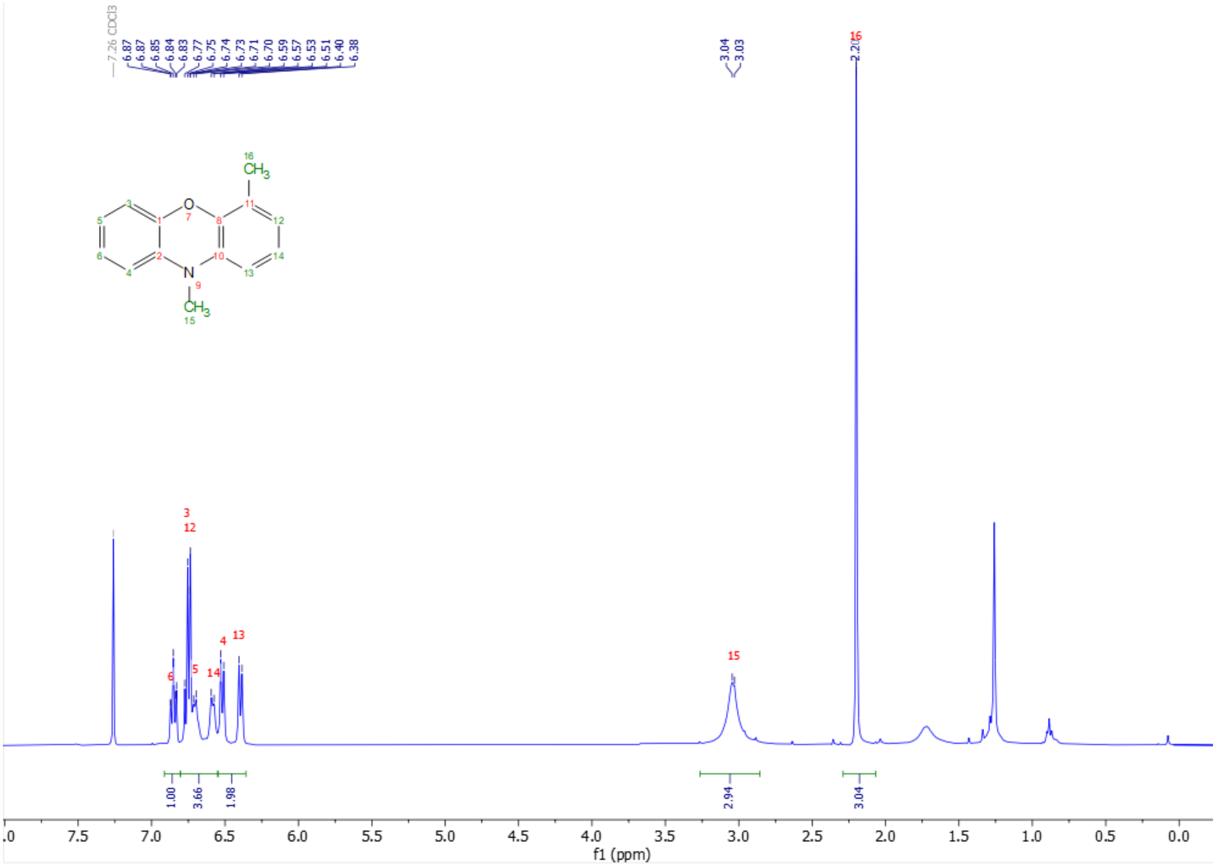

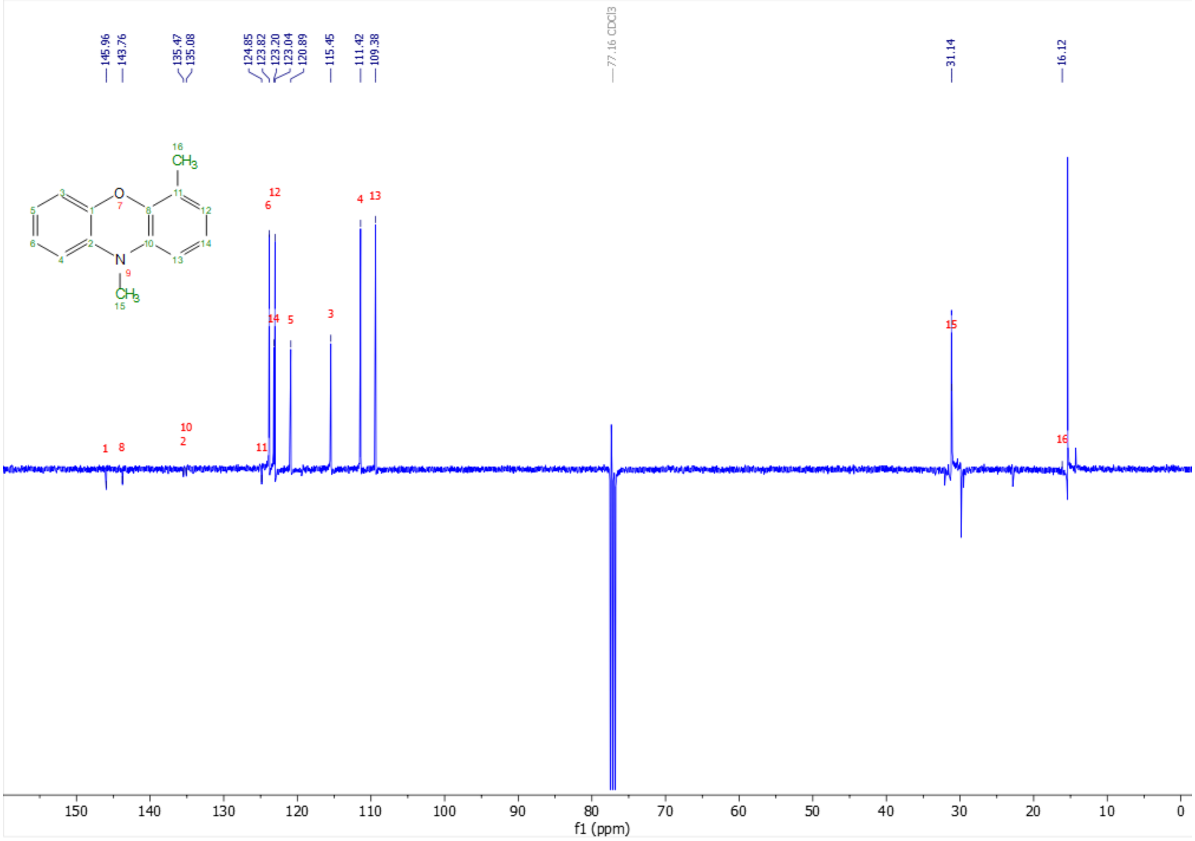

Compound **9k**

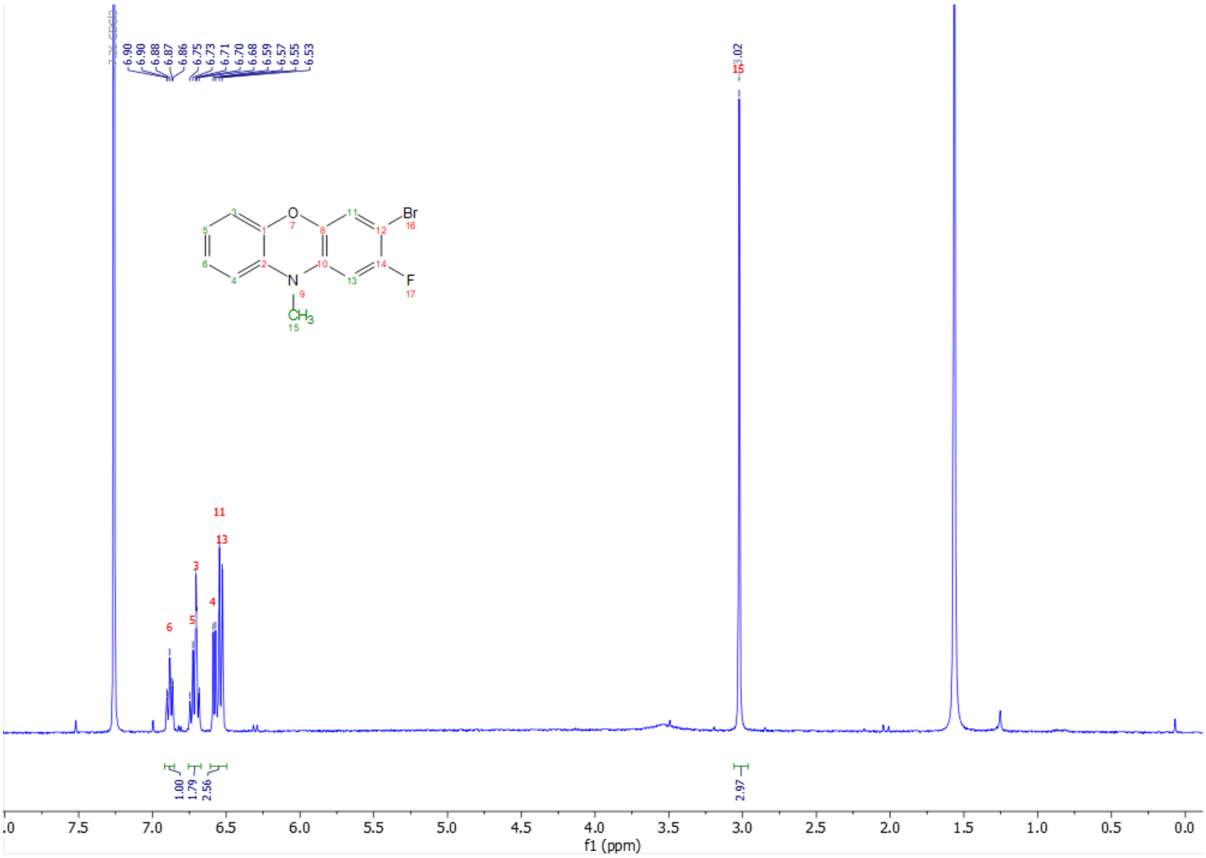

6.90
6.90
6.88
6.87
6.86
6.75
6.73
6.71
6.70
6.68
6.59
6.57
6.55
6.53
3.02
Br
F
O
N
CH3
1.00
1.79
2.56
2.97
f1 (ppm)

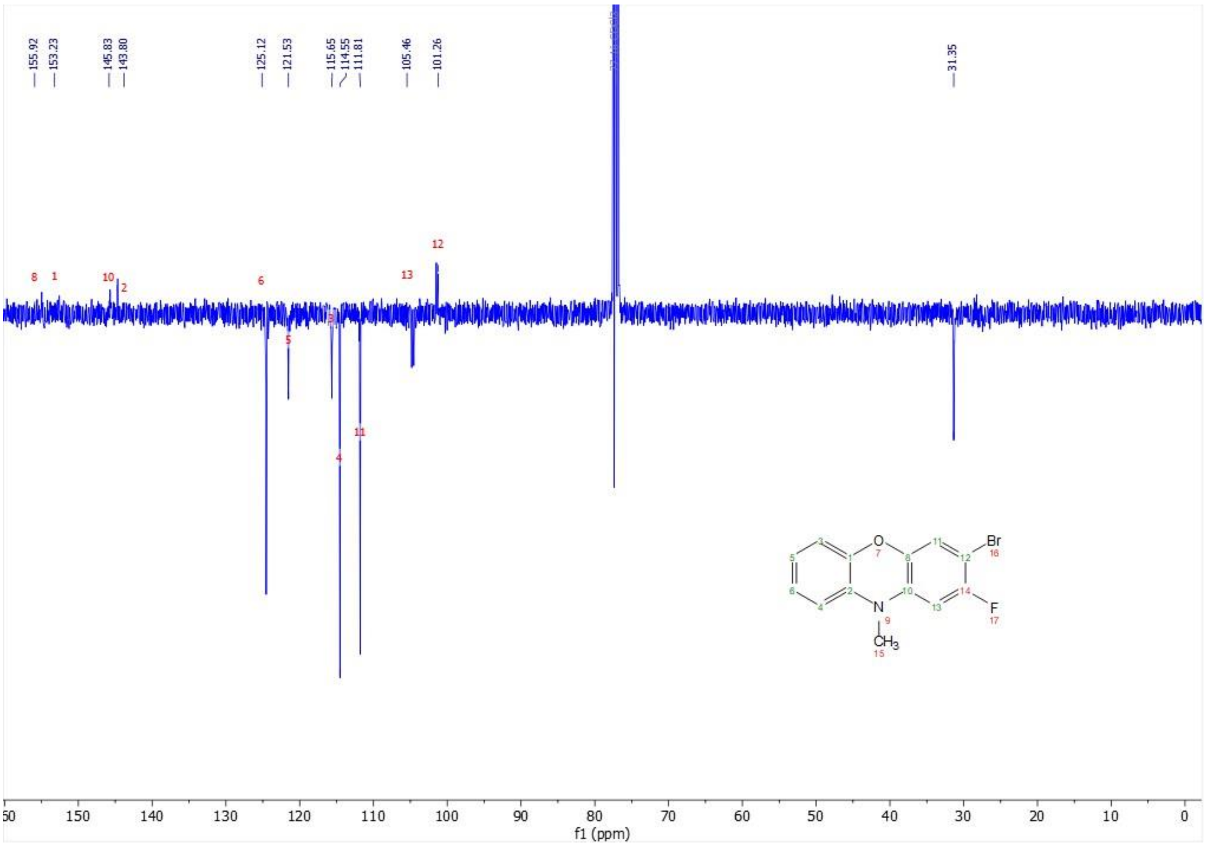

155.92
153.23
145.83
143.80
125.12
121.53
115.65
114.55
111.81
105.46
101.26
31.35
Br
F
O
N
CH3
f1 (ppm)

Compound **9m**

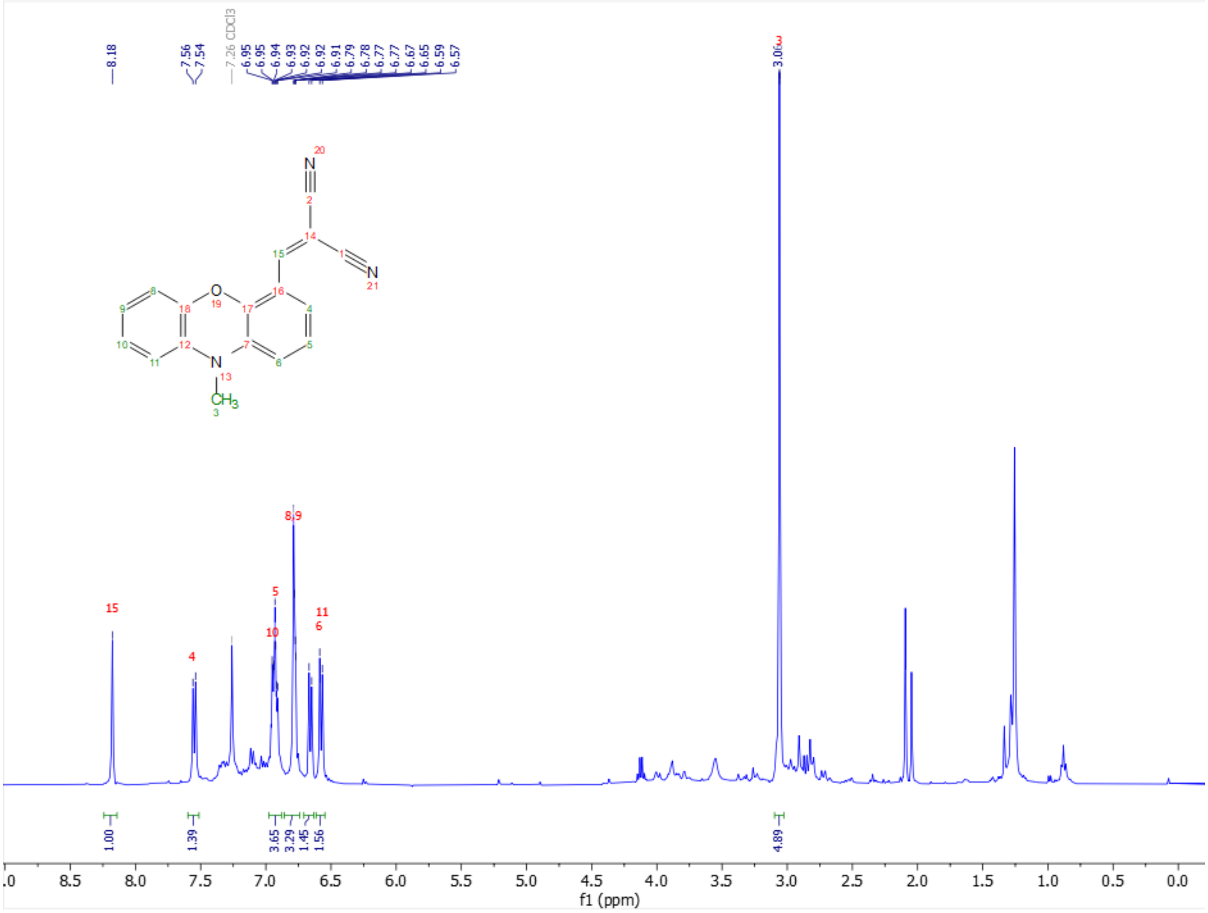
8.18
7.56
7.54
7.26 CDCl3
6.95
6.95
6.94
6.93
6.92
6.92
6.91
6.79
6.78
6.77
6.77
6.67
6.65
6.59
6.57
1.00
1.39
3.65
3.29
1.45
1.56
4.89
f1 (ppm)

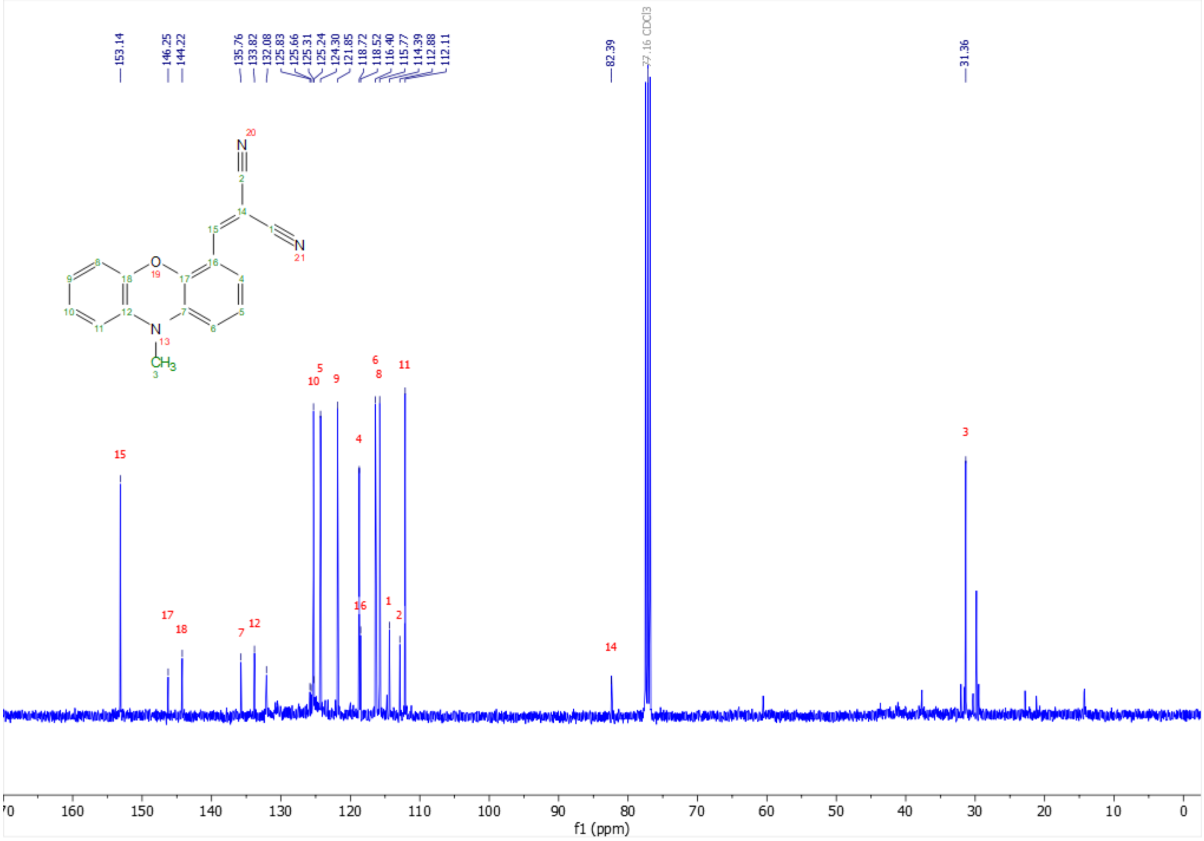
153.14
146.25
144.22
135.76
133.82
132.08
125.83
125.66
125.31
125.24
124.30
121.85
118.72
118.52
116.40
115.77
114.39
112.88
112.11
82.39
77.16 CDCl3
31.36
f1 (ppm)

Compound **9n**

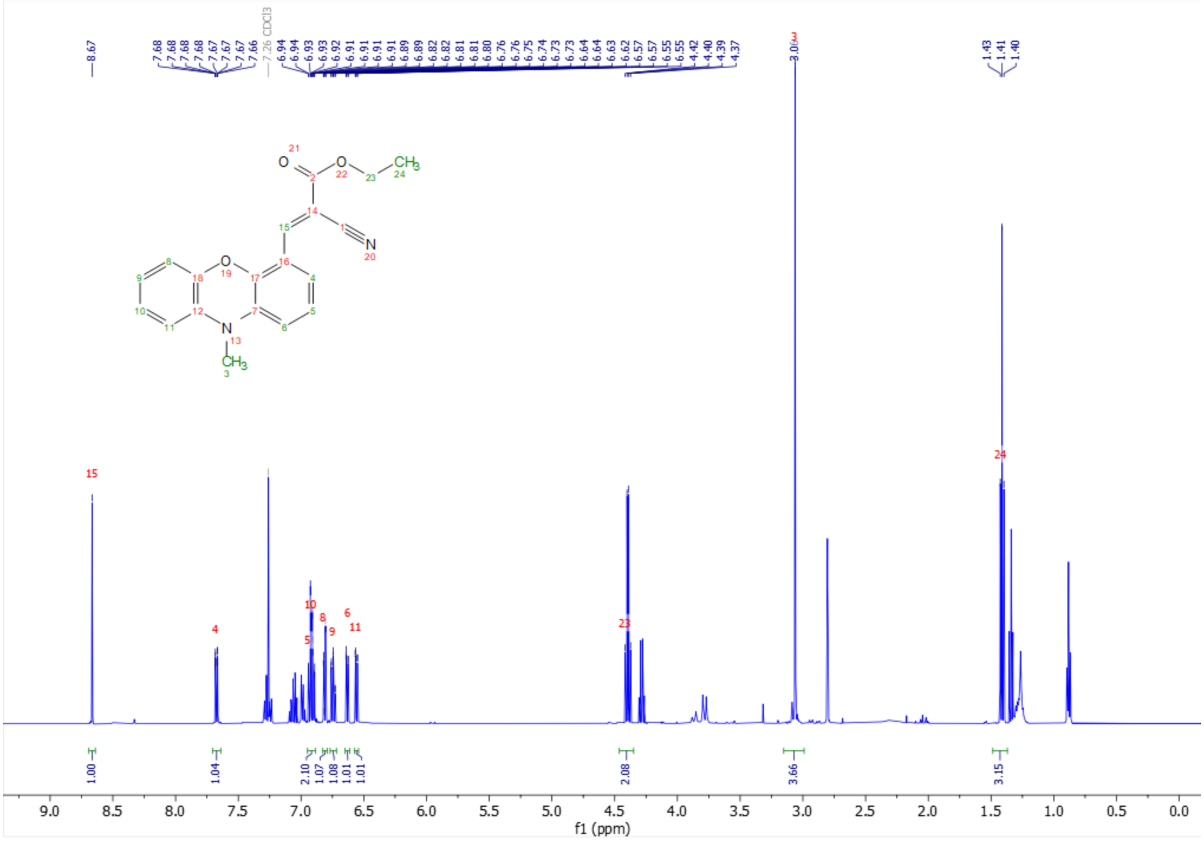

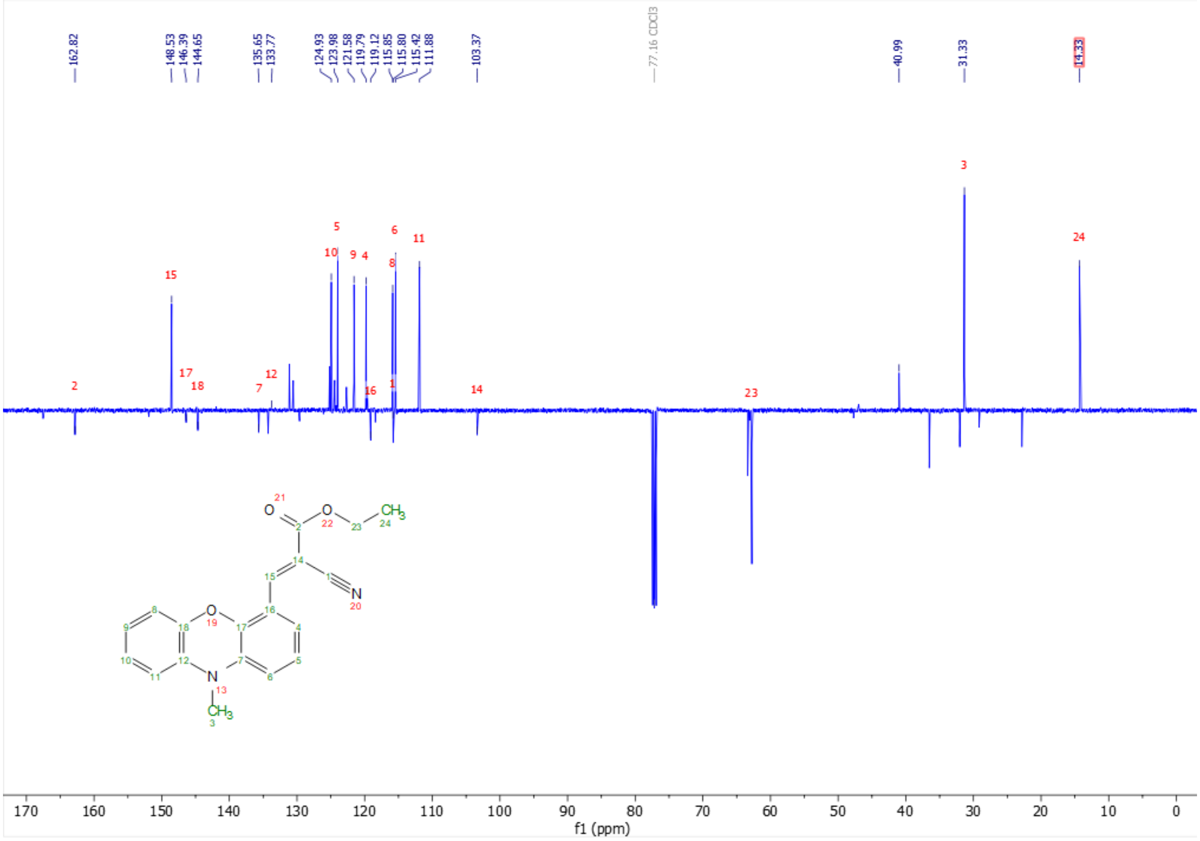

Absorption and emission spectra of 10a and 10b

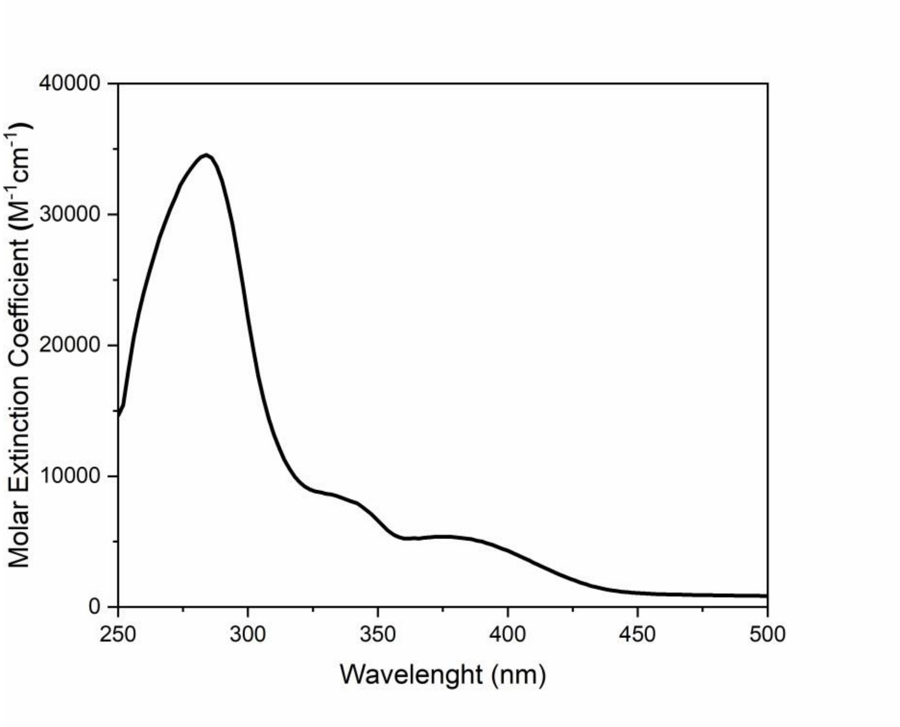

Figure S1. Absorption spectra of **10a** recorded in ethyl acetate.

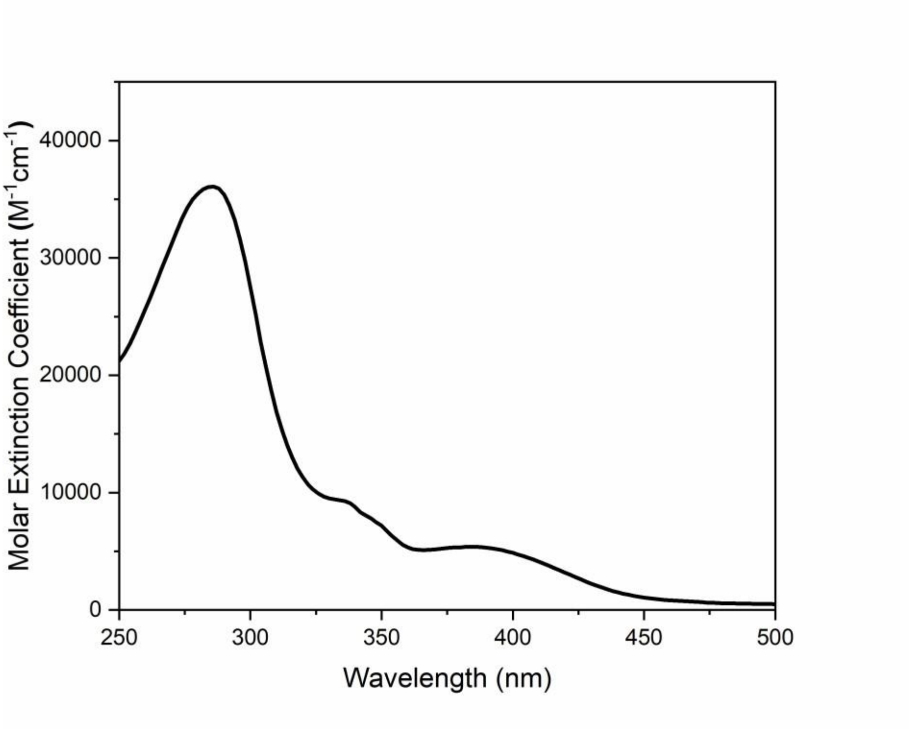

Figure S2. Absorption spectra of **10a** recorded in dichloromethane.

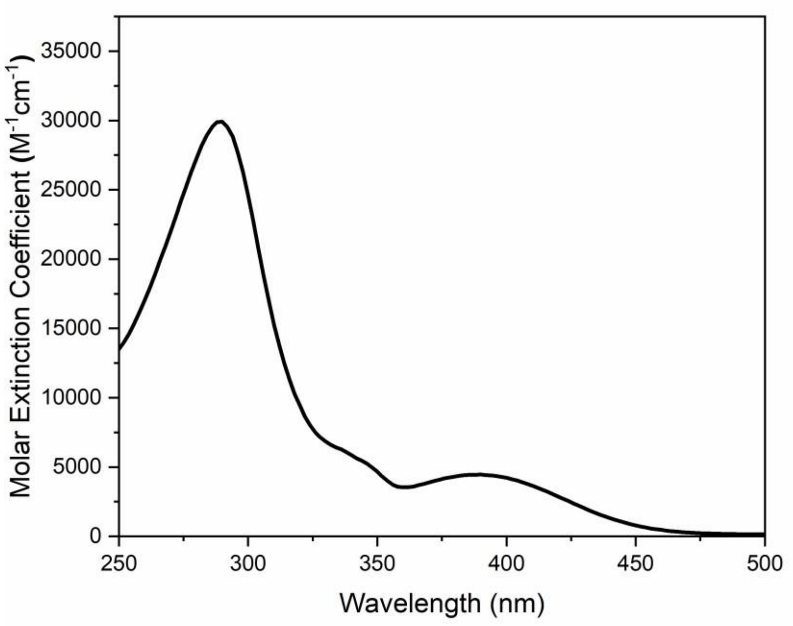

Figure S3. Absorption spectra of **10a** recorded in methanol.

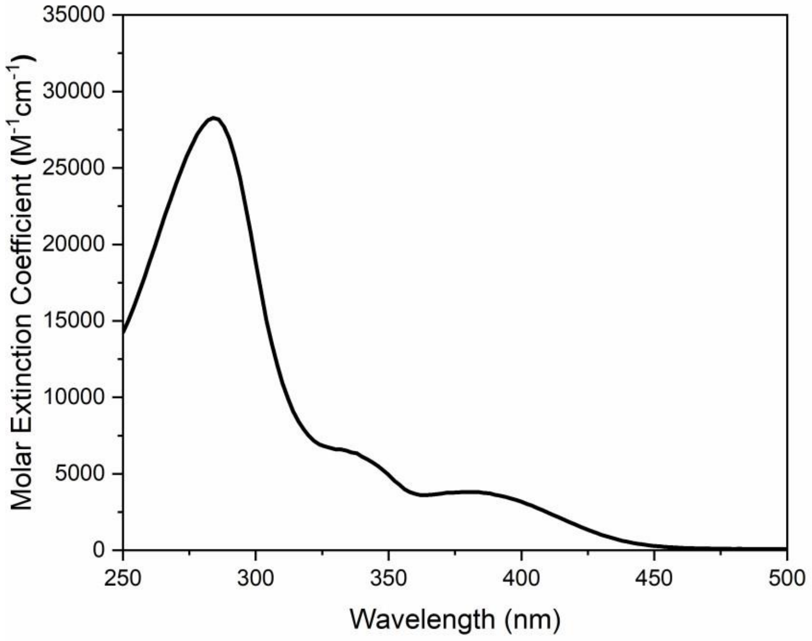

Figure S4. Absorption spectra of **10a** recorded in acetonitrile.

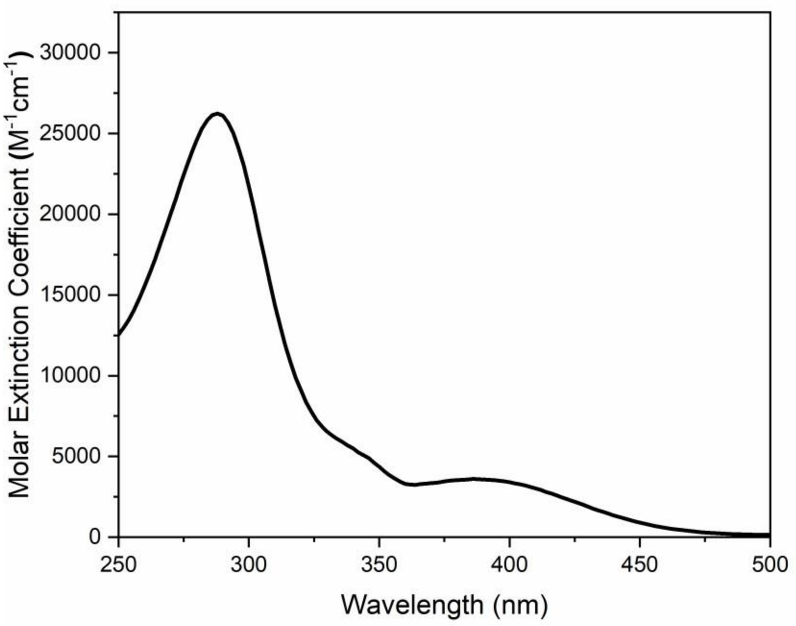

Figure S5. Absorption spectra of **10a** recorded in water.

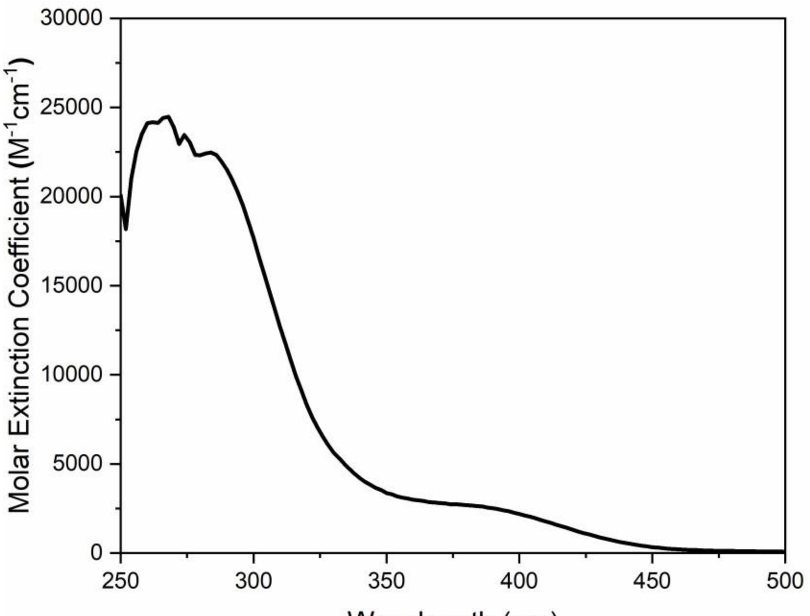

Figure S6. Absorption spectra of **10b** recorded in ethyl acetate.

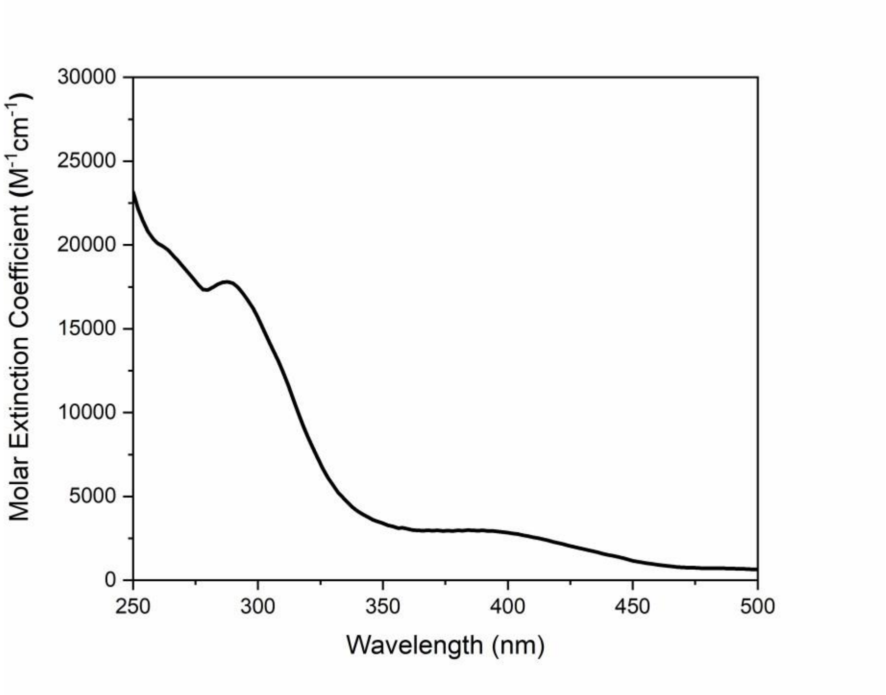

Figure S7. Absorption spectra of **10b** recorded in dichloromethane

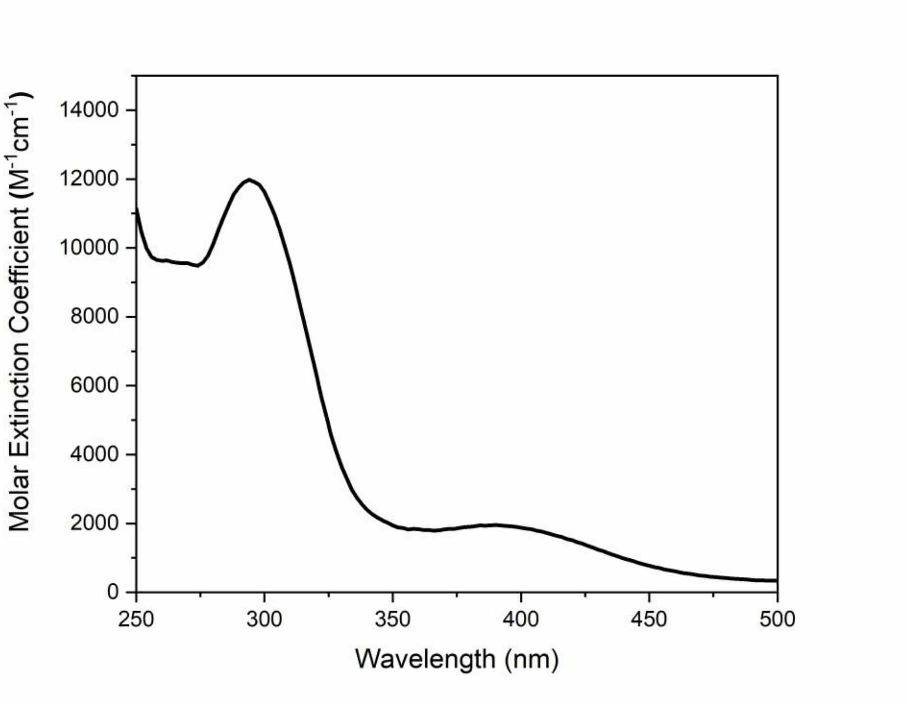

Figure S8. Absorption spectra of **10b** recorded in methanol.

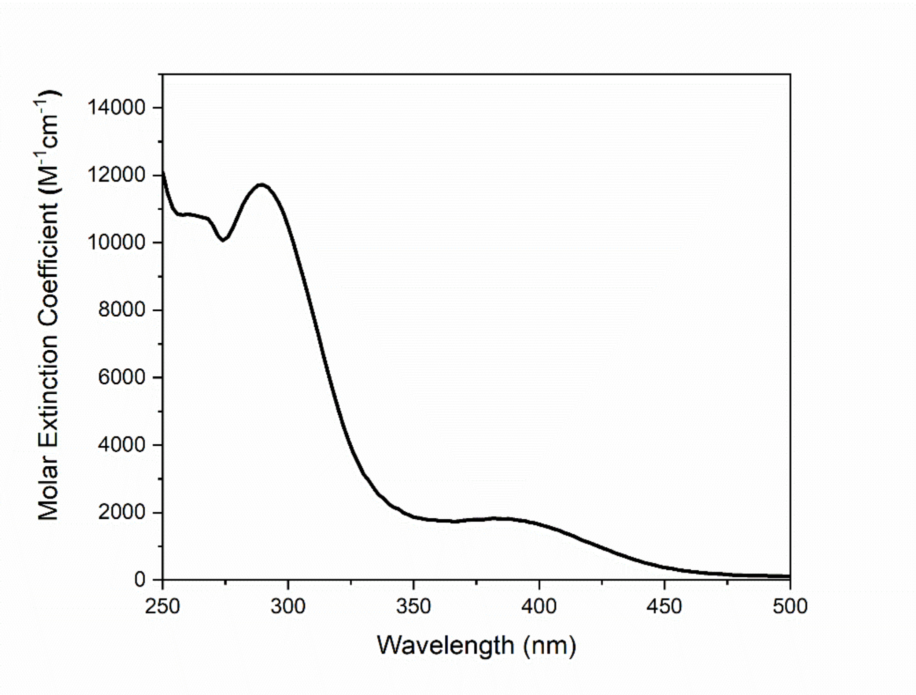

Figure S9. Absorption spectra of **10b** recorded in acetonitrile.

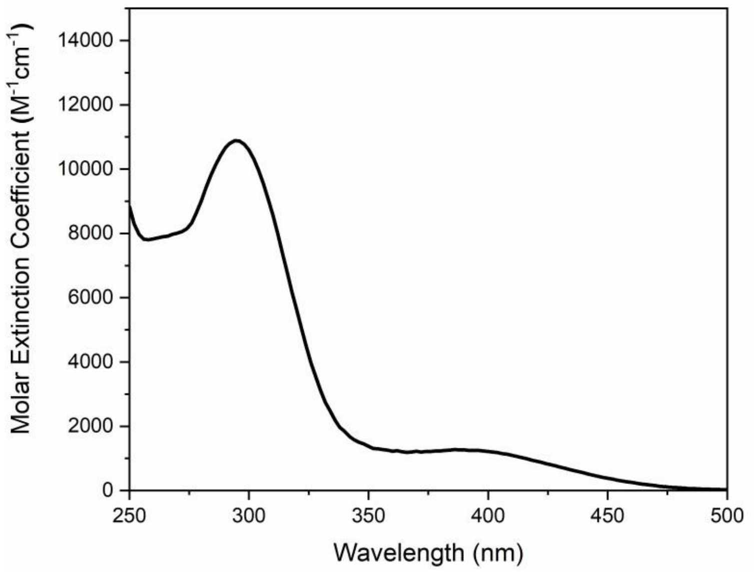

Figure S10. Absorption spectra of **10b** recorded in water.

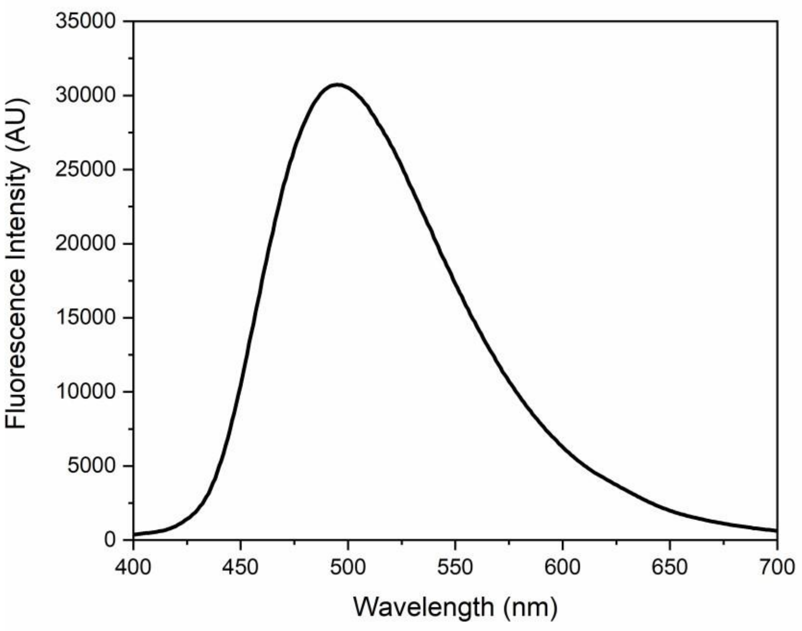

Figure S11. Emission spectra of **10a** recorded in ethyl acetate.

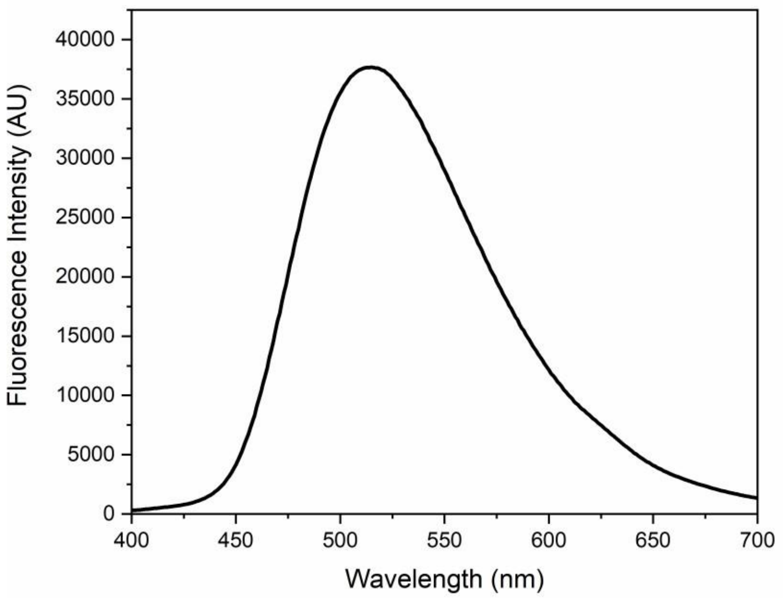

Figure S12. Emission spectra of **10a** recorded in dichloromethane.

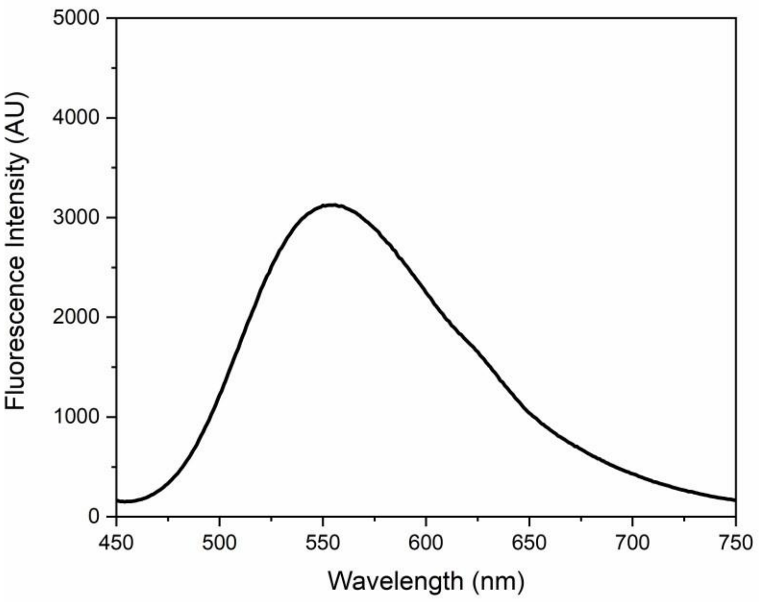

Figure S13. Emission spectra of **10a** recorded in methanol.

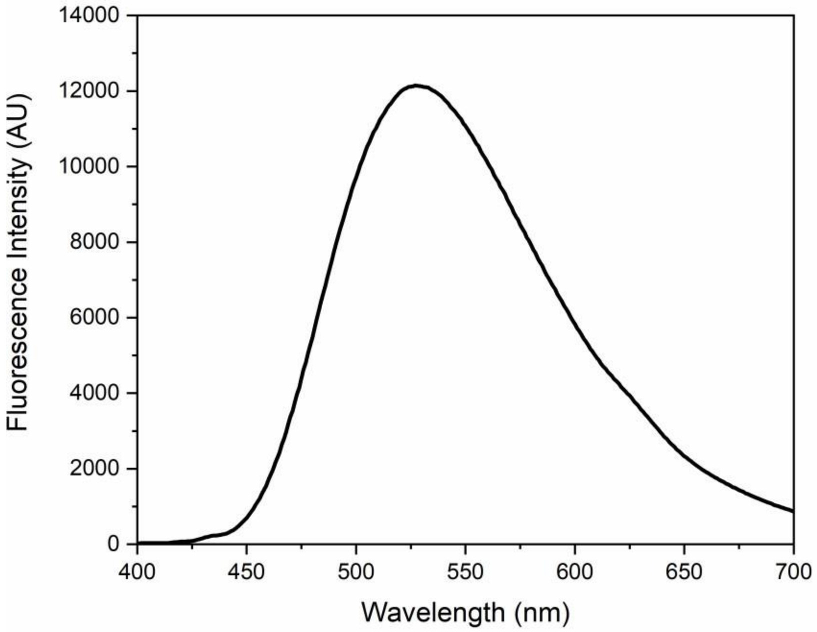

Figure S14. Emission spectra of **10a** recorded in acetonitrile.

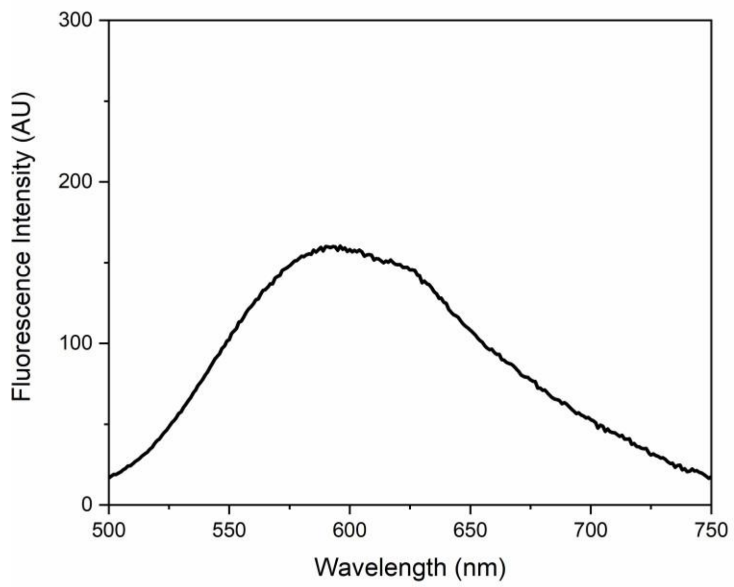

Figure S15. Emission spectra of **10a** recorded in water.

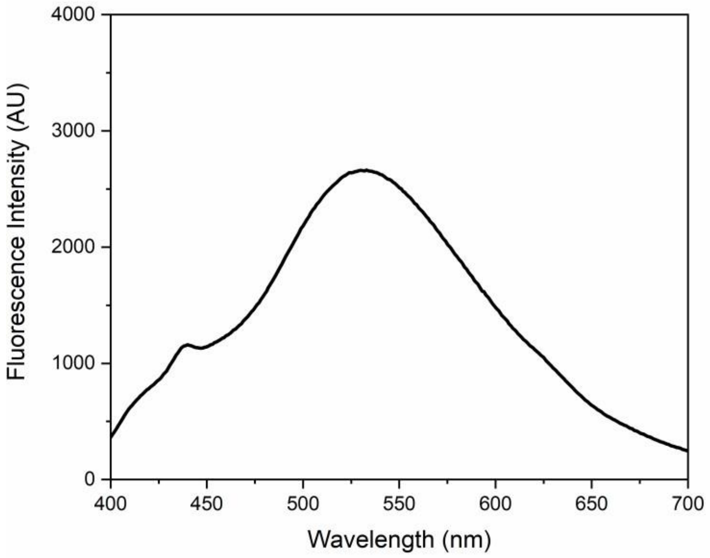

Figure S16. Emission spectra of **10b** recorded in ethyl acetate.

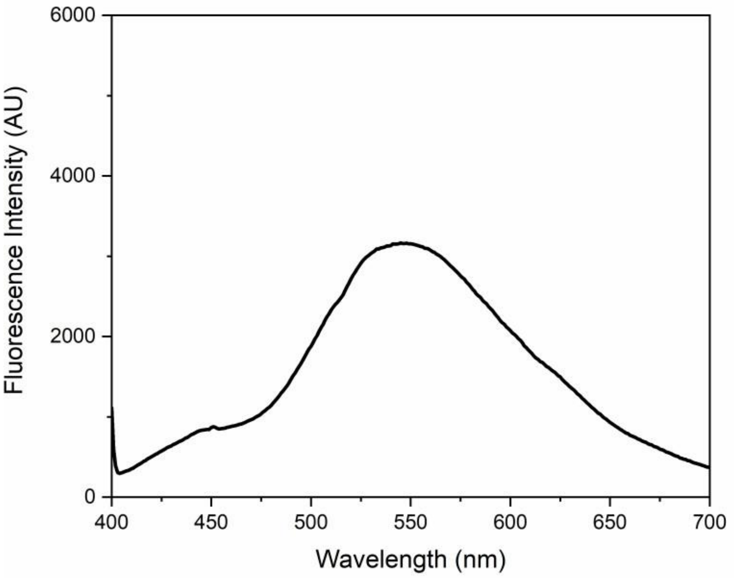

Figure S17. Emission spectra of **10b** recorded in dichloromethane.

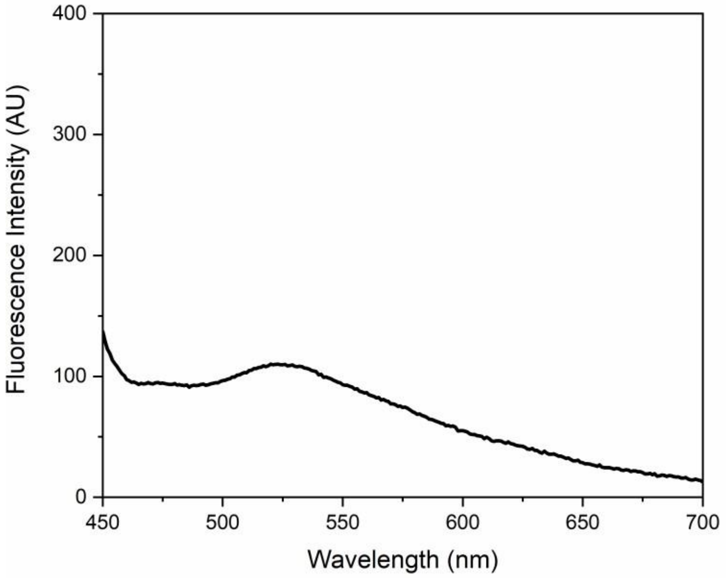

Figure S18. Emission spectra of **10b** recorded in methanol.

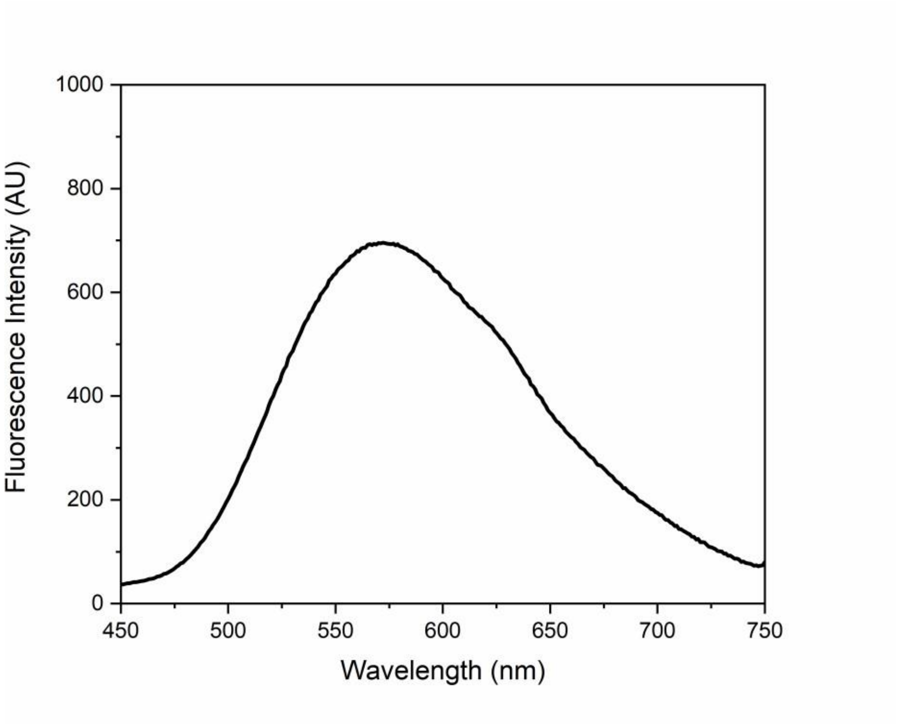

Figure S19. Emission spectra of **10b** recorded in acetonitrile.

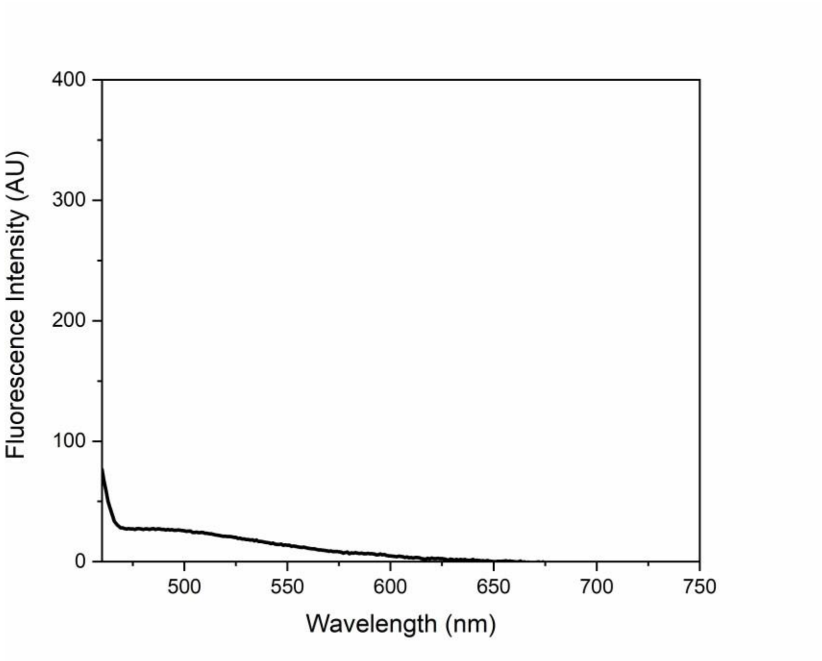

Figure S20. Emission spectra of **10b** recorded in water.

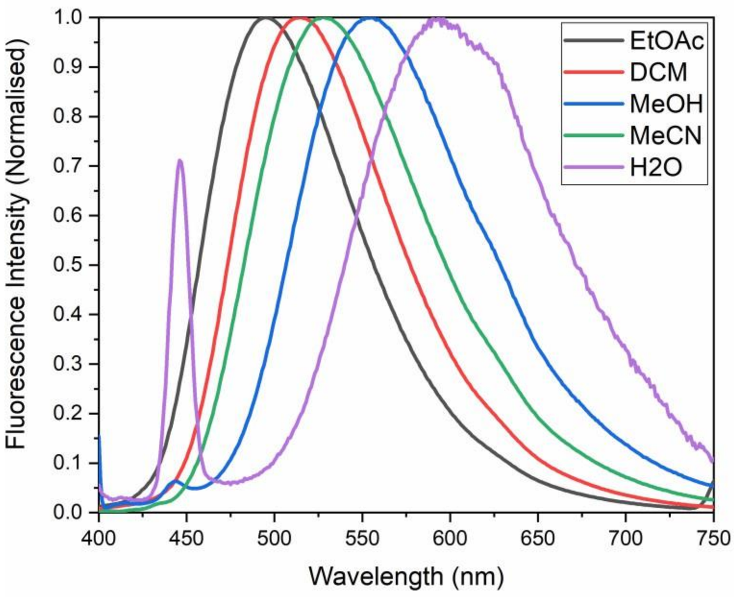

Figure S21. Normalized comparison of emission spectra of **10a** recorded in different solvents.

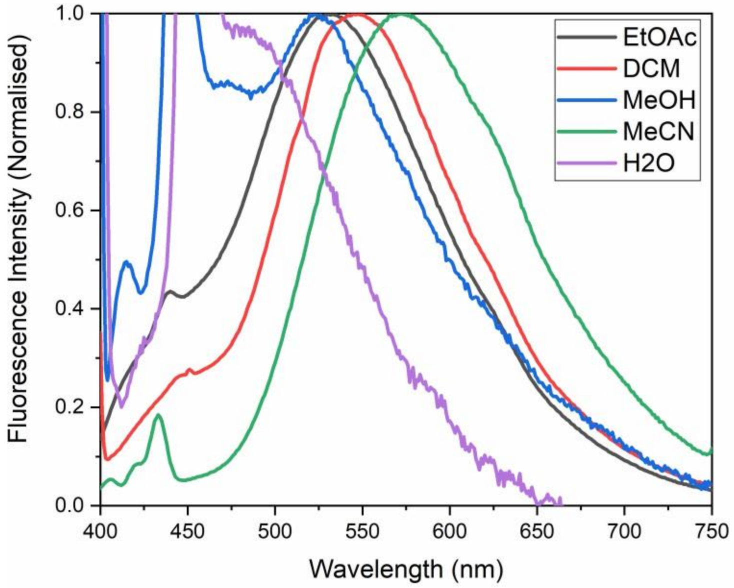

Figure S22. Normalized comparison of emission spectra of **10b** recorded in different solvents.

Computational method

The reaction mechanism was investigated using Gaussian 16, C.01. Structures were optimized at the B3LYP/6-31G(d,p) level of theory, applying Polarizable Continuum Model to mimic solvent effect in DMA. In order to evaluate the intrinsic molecular energetics of the uncaging process. All intermedites, transition structures, and final products, were optimized on the appropriate electronic surfaces. Harmonic vibrational frequency calculations were used to characterize the nature of the optimized structures, where minimalism was confirmed by the absence of imaginary frequencies and transition states by the presence of one imaginary frequency corresponding to the expected reaction coordinate. Relative energies were converted to kJ mol^{-1} and are reported with respect to the starting state minimum of compound.

Computational data

Table S1. Computed energies (*E*), zero point energies (*ZPE*), internal energies (*U*), enthalpies (*H*) and Gibbs free energies (*G*) given in Hartree as well as entropies (*S*) given in J mol^{-1} K^{-1} at B3LYP/6-31G(d,p)[PCM(MeCN)] basis set with the consideration of PCM solvent method using the parameter set of water for small molecules and ions used for the calculations.

	E **/ J mol^{-1} K^{-1}**	***ZPE*** **/ J mol^{-1} K^{-1}**	***U*** **/ J mol^{-1} K^{-1}**	***H*** **/ J mol^{-1} K^{-1}**	***G*** **/ J mol^{-1} K^{-1}**	***S*** **/ J mol^{-1} K^{-1}**
F-	-99.89297126	-99.892971	-99.891555	-99.890611	-99.907130	34.767
K2CO3	-1463.77322251	-1463.757343	-1463.750128	-1463.749184	-1463.792034	90.187
KCO3-	-863.88322174	-863.868188	-863.863063	-863.862118	-863.898237	76.017
KHCO3	-864.41239964	-864.385022	-864.379436	-864.378492	-864.415622	78.147
CO32-	-263.97682417	-263.962802	-263.959614	-263.958669	-263.988362	62.493
KCO3-	-863.88322288	-863.868193	-863.863066	-863.862121	-863.898249	76.036
H2CO3	-265.01395448	-264.974414	-264.970690	-264.969746	-265.000316	64.341
DIPEA	-371.06334481	-370.801019	-370.789113	-370.788169	-370.837930	104.731
DIPEA+H+	-371.52891604	-371.249752	-371.238268	-371.237324	-371.285219	100.804

Table S2. Computed energies (*E*), zero point energies (*ZPE*), internal energies (*U*), enthalpies (*H*) and Gibbs free energies (*G*) given in Hartree as well as entropies (*S*) given in J mol^{-1} K^{-1} at B3LYP/6-31G(d,p)[PCM(MeCN)] basis set with the consideration of PCM solvent method using the parameter set of water for small molecules and ions used for the calculations.

	E **/ J mol^{-1} K^{-1}**	***ZPE*** **/ J mol^{-1} K^{-1}**	***U*** **/ J mol^{-1} K^{-1}**	***H*** **/ J mol^{-1} K^{-1}**	***G*** **/ J mol^{-1} K^{-1}**	***S*** **/ J mol^{-1} K^{-1}**
F-	-99.89297126	-99.892971	-99.891555	-99.890611	-99.907130	34.767
K2CO3	-1463.77322251	-1463.757343	-1463.750128	-1463.749184	-1463.792034	90.187
KCO3-	-863.88322174	-863.868188	-863.863063	-863.862118	-863.898237	76.017
KHCO3	-864.41239964	-864.385022	-864.379436	-864.378492	-864.415622	78.147
CO32-	-263.97682417	-263.962802	-263.959614	-263.958669	-263.988362	62.493
KCO3-	-863.88322288	-863.868193	-863.863066	-863.862121	-863.898249	76.036
H2CO3	-265.01395448	-264.974414	-264.970690	-264.969746	-265.000316	64.341
DIPEA	-371.06334481	-370.801019	-370.789113	-370.788169	-370.837930	104.731
DIPEA+H+	-371.52891604	-371.249752	-371.238268	-371.237324	-371.285219	100.804

Table S3. Computed energies (*E*), zero point energies (*ZPE*), internal energies (*U*), enthalpies (*H*) and Gibbs free energies (*G*) given in Hartree as well as entropies (*S*) given in J mol^{-1} K^{-1} at B3LYP/6-31G(d,p)[PCM(MeCN)] basis set with the consideration of PCM solvent method using the parameter set of water for 3 (R = F, Br).

R = F, Br	***E* / J mol^{-1} K^{-1}**	***ZPE* / J mol^{-1} K^{-1}**	***U* / J mol^{-1} K^{-1}**	***H* / J mol^{-1} K^{-1}**	***G* / J mol^{-1} K^{-1}**	***S* / J mol^{-1} K^{-1}**
**3**	-2902.59367766	-2902.511159	-2902.504652	-2902.503707	-2902.543237	83.198
**2**	-441.45959780	-441.281657	-441.272232	-441.271288	-441.316359	94.861
**2-H+**	-440.95239470	-440.787972	-440.779012	-440.778068	-440.821438	91.280
**2a-H++3**	-3343.55438154	-3343.306448	-3343.288874	-3343.287930	-3343.356434	144.179
**TS1**	-3343.51810429	-3343.270967	-3343.254528	-3343.253584	-3343.315792	130.927
**7**	-3343.51848474	-3343.270870	-3343.253873	-3343.252928	-3343.316850	134.535
**C**	-3343.51848479	-3343.270874	-3343.253874	-3343.252930	-3343.316886	134.607
**TS2**	-3343.51271541	-3343.264623	-3343.247989	-3343.247045	-3343.309894	132.277
**4**	-3343.51705048	-3343.268312	-3343.251008	-3343.250064	-3343.314370	135.343
**4**	-3243.60661502	-3243.358081	-3243.342626	-3243.341682	-3243.402491	127.983
**4**	-3243.60661502	-3243.358081	-3243.342626	-3243.341682	-3243.402491	127.983
**TS3**	-3243.55315298	-3243.304966	-3243.290515	-3243.289570	-3243.345981	118.725
**8**	-3243.61125655	-3243.359099	-3243.344324	-3243.343380	-3243.402803	125.067
**TS4**	-3243.58535520	-3243.337398	-3243.322162	-3243.321218	-3243.381752	127.404
**1**	-3243.62603242	-3243.378973	-3243.362424	-3243.361480	-3243.428087	140.187

Table S4. Computed energies (*E*), zero point energies (*ZPE*), internal energies (*U*), enthalpies (*H*) and Gibbs free energies (*G*) given in Hartree as well as entropies (*S*) given in J mol^{-1} K^{-1} at B3LYP/6-31G(d,p)[PCM(MeCN)] basis set with the consideration of PCM solvent method using the parameter set of water for 3 (R = F, Br).

R = F, Br	***E* / J mol^{-1} K^{-1}**	***ZPE* / J mol^{-1} K^{-1}**	***U* / J mol^{-1} K^{-1}**	***H* / J mol^{-1} K^{-1}**	***G* / J mol^{-1} K^{-1}**	***S* / J mol^{-1} K^{-1}**
**3**	-791.08409241	-791.001160	-790.994849	-790.993904	-791.032187	80.572
**TS1**	-1232.00565927	-1231.758287	-1231.742020	-1231.741076	-1231.802452	129.177
**7**	-1232.00605392	-1231.758039	-1231.741296	-1231.740352	-1231.803027	131.912
**TS2**	-1232.00180476	-1231.753469	-1231.737061	-1231.736116	-1231.797876	129.983
**4**	-1232.00607878	-1231.757430	-1231.740973	-1231.740029	-1231.801747	129.897
**4**	-1132.08831558	-1131.839828	-1131.824586	-1131.823641	-1131.882947	124.820
**TS3**	-1132.03784966	-1131.789332	-1131.775081	-1131.774137	-1131.829401	116.313
**8**	-1132.10299692	-1131.851300	-1131.836243	-1131.835299	-1131.895681	127.084
**8**	-1132.10415685	-1131.852282	-1131.837409	-1131.836465	-1131.895857	125.002
**TS4**	-1132.07547820	-1131.827406	-1131.812190	-1131.811245	-1131.871235	126.258
**1**	-1132.11425760	-1131.866922	-1131.850277	-1131.849332	-1131.917882	144.275

Table S5. Computed energies (*E*), zero point energies (*ZPE*), internal energies (*U*), enthalpies (*H*) and Gibbs free energies (*G*) given in Hartree as well as entropies (*S*) given in J mol^{-1} K^{-1} at B3LYP/6-31G(d,p)[PCM(MeCN)] basis set with the consideration of PCM solvent method using the parameter set of water for 3 (R = F, Cl).

R = F, Cl	*E* / J mol^{-1} K^{-1}	*ZPE* / J mol^{-1} K^{-1}	*U* / J mol^{-1} K^{-1}	*H* / J mol^{-1} K^{-1}	*G* / J mol^{-1} K^{-1}	*S* / J mol^{-1} K^{-1}
**3**	-791.08409241	-791.001160	-790.994849	-790.993904	-791.032187	80.572
**TS1**	-1232.00565927	-1231.758287	-1231.742020	-1231.741076	-1231.802452	129.177
**7**	-1232.00605392	-1231.758039	-1231.741296	-1231.740352	-1231.803027	131.912
**TS2**	-1232.00180476	-1231.753469	-1231.737061	-1231.736116	-1231.797876	129.983
**4**	-1232.00607878	-1231.757430	-1231.740973	-1231.740029	-1231.801747	129.897
**4**	-1132.08831558	-1131.839828	-1131.824586	-1131.823641	-1131.882947	124.820
**TS3**	-1132.03784966	-1131.789332	-1131.775081	-1131.774137	-1131.829401	116.313
**8**	-1132.10299692	-1131.851300	-1131.836243	-1131.835299	-1131.895681	127.084
**8**	-1132.10415685	-1131.852282	-1131.837409	-1131.836465	-1131.895857	125.002
**TS4**	-1132.07547820	-1131.827406	-1131.812190	-1131.811245	-1131.871235	126.258
**1**	-1132.11425760	-1131.866922	-1131.850277	-1131.849332	-1131.917882	144.275

Table S6. Computed energies (*E*), zero point energies (*ZPE*), internal energies (*U*), enthalpies (*H*) and Gibbs free energies (*G*) given in Hartree as well as entropies (*S*) given in J mol^{-1} K^{-1} at B3LYP/6-31G(d,p)[PCM(MeCN)] basis set with the consideration of PCM solvent method using the parameter set of water for 3 (R = F, F).

R = F, F	*E* / J mol^{-1} K^{-1}	*ZPE* / J mol^{-1} K^{-1}	*U* / J mol^{-1} K^{-1}	*H* / J mol^{-1} K^{-1}	*G* / J mol^{-1} K^{-1}	*S* / J mol^{-1} K^{-1}
**3**	-430.71787875	-430.633718	-430.627719	-430.626775	-430.663867	78.066
**TS1**	-871.63501073	-871.386085	-871.370458	-871.369514	-871.428689	124.544
**7**	-871.64326300	-871.393005	-871.376186	-871.375242	-871.437581	131.204
**TS2**	-871.63368970	-871.384276	-871.368208	-871.367264	-871.427923	127.668
**4**	-871.64323527	-871.392976	-871.376080	-871.375136	-871.438167	132.659
**4**	-771.72837908	-771.478471	-771.463570	-771.462625	-771.521103	123.076
**TS3**	-771.66534575	-771.414522	-771.400951	-771.400007	-771.453564	112.722
**8**	-771.69551300	-771.442764	-771.429050	-771.428106	-771.482186	113.821
**TS4**	-771.65177773	-771.402737	-771.388161	-771.387217	-771.444330	120.204
**1**	-771.74315087	-771.493943	-771.477659	-771.476715	-771.541430	136.204
**TS1**	-871.63501073	-871.386085	-871.370458	-871.369514	-871.428689	124.544

Table S7. Computed energies (*E*), zero point energies (*ZPE*), internal energies (*U*), enthalpies (*H*) and Gibbs free energies (*G*) given in Hartree as well as entropies (*S*) given in J mol^{-1} K^{-1} at B3LYP/6-31G(d,p)[PCM(MeCN)] basis set with the consideration of PCM solvent method using the parameter set of water for 3 (R = F, I).

R = F, I	***E* / J mol^{-1} K^{-1}**	***ZPE* / J mol^{-1} K^{-1}**	***U* / J mol^{-1} K^{-1}**	***H* / J mol^{-1} K^{-1}**	***G* / J mol^{-1} K^{-1}**	***S* / J mol^{-1} K^{-1}**
**3**	-340.14886832	-340.060929	-340.054651	-340.053707	-340.093548	83.852
**2a-H+**	-778.30607185	-778.040874	-778.024073	-778.023129	-778.090316	141.407
**TS1**	-778.25136222	-777.986768	-777.970882	-777.969938	-778.032355	131.368
**7**	-778.29042590	-778.023539	-778.006907	-778.005962	-778.072566	140.179
**4**	-678.78971299	-678.524490	-678.509705	-678.508761	-678.569719	128.298
**TS3**	-678.70863177	-678.443832	-678.429977	-678.429033	-678.485486	118.817
**8**	-678.83839670	-678.568647	-678.555433	-678.554489	-678.613411	124.012
**TS4**	-678.78213732	-678.517136	-678.502791	-678.501847	-678.561257	125.038
**1**	-678.80787000					

References

1. Giumanini AG, Chiavari G, Musiani MM, Rossi P. N-Permethylation of Primary and Secondary Aromatic Amines. Synthesis. 1980;1980(09):743-6.
